# Transcriptome-wide survey of gene expression changes and alternative splicing in *Trichophyton rubrum* in response to undecanoic acid

**DOI:** 10.1038/s41598-018-20738-x

**Published:** 2018-02-06

**Authors:** Niege S. Mendes, Tamires A. Bitencourt, Pablo R. Sanches, Rafael Silva-Rocha, Nilce M. Martinez-Rossi, Antonio Rossi

**Affiliations:** 1 Department of Genetics, Ribeirão Preto Medical School, University of São Paulo, 14049-900 Ribeirão Preto, SP Brazil; 2 Department of Molecular and Cellular Biology, Ribeirão Preto Medical School, University of São Paulo, 14049-900 Ribeirão Preto, SP Brazil

## Abstract

While fatty acids are known to be toxic to dermatophytes, key physiological aspects of the *Trichophyton rubrum* response to undecanoic acid (UDA), a medium chain saturated fatty acid (C_11:0_), are not well understood. Thus, we analysed RNA-seq data from *T. rubrum* exposed to sub-lethal doses of UDA for 3 and 12 h. Three putative pathways were primarily involved in UDA detoxification: lipid metabolism and cellular membrane composition, oxidative stress, and pathogenesis. Biochemical assays showed cell membrane impairment, reductions in ergosterol content, and an increase in keratinolytic activity following UDA exposure. Moreover, we assessed differential exon usage and intron retention following UDA exposure. A key enzyme supplying guanine nucleotides to cells, inosine monophosphate dehydrogenase (IMPDH), showed high levels of intron 2 retention. Additionally, phosphoglucomutase (PGM), which is involved in the glycogen synthesis and degradation as well as cell wall biosynthesis, exhibited a significant difference in exon 4 usage following UDA exposure. Owing to the roles of these enzymes in fungal cells, both have emerged as promising antifungal targets. We showed that intron 2 retention in *impdh* and exon 4 skipping in *pgm* might be related to an adaptive strategy to combat fatty acid toxicity. Thus, the general effect of UDA fungal toxicity involves changes to fungal metabolism and mechanisms for regulating pre-mRNA processing events.

## Introduction

Dermatophytes are filamentous fungi that are able to infect keratinized tissues, such as the nails, hair, and skin^1^. They are highly pathogenic, causing most cases of superficial mycosis worldwide^2,3^. The anthropophilic species *Trichophyton rubrum* is a cosmopolitan dermatophyte that is the most common etiologic agent isolated from clinical cases of superficial mycosis worldwide. While fungal infections do not cause pandemics, dermatophytoses are endemic in many parts of the world, causing significant suffering^4^. In addition, dermatophytoses are an important public health issue, as they may cause deep infections in at-risk patients under medical care, including immunocompromised patients^1,5^. The clinical treatment of these cutaneous infections is challenging because only a few antifungal drugs are commercially available and because treatment is lengthy and costly. Most commercially available antifungal medicines, which fall under the azole and allylamine/thiocarbamate classes, target ergosterol biosynthetic enzymes. Ergosterol, a cholesterol analogue, is the principal sterol of the fungal plasma membrane and contributes to a variety of cell functions, such as fluidity and integrity, which are crucial for cell growth and division^6^. However, several cases of resistance to antifungals have been reported, indicating a need to identify new therapeutic targets and new antifungal drugs^7^.

In this context, the antimycotic activity of fatty acids has long been known. Their presence in human skin and sweat acts as a barrier against cutaneous infections^8^. Undecanoic acid (UDA), a medium-chain fatty acid (C_11:0_), is the most fungitoxic compound in the C_7:0_–C_18:0_ series and has been used in the treatment of superficial mycoses in humans^8,9^. UDA inhibits vegetative growth, conidial germination, cellular respiration^10^, carbohydrate metabolism^11^, phosphate uptake^12^, and phospholipid metabolism^8^. Its effects on diverse cellular processes that are seemingly unrelated suggest non-specific interactions with fungal cell proteins and enzymes, as well as with fatty acid metabolism in *T. rubrum*. This reduces the chance for resistance and strengthens its clinical utility. Furthermore, UDA suppresses the secretion of lipases and keratinases and stimulates the secretion of phospholipase A^8,13,14^.

During the infection process, dermatophyte–host interactions trigger the transcription of genes that allow for the adherence and penetration of the pathogen into the host tissue, enabling it to scavenge nutrients and overcome host defence mechanisms. This genetic adaptive machinery leads to cell wall remodelling and the secretion of a battery of endo- and exoproteases that degrade keratinized structures, using them as nutrients^15–17^. A key part of this strategy is the pathogen’s ability to alter the expression of its genetic machinery, including alternative splicing (AS), in response to natural host defences, and antifungal therapies. The AS is a regulated molecular mechanism that increases protein diversity and therefore plays an important role in post-transcriptional events. Fungi responds to nutrient signalling, drug exposure, and environmental pH changes with AS^18,19^.

Recently, the sequencing of seven dermatophyte genomes, including that of *T. rubrum*, provided a basis for future genome-supported studies on the biology, pathogenicity, and host specificity of this important group of pathogens, promoting the identification of molecular targets^20,21^. In this study, we assessed the transcriptome of the pathogenic fungus *T*. *rubrum* through RNA-sequencing (RNA-seq) analysis to identify transcriptome-wide changes in gene expression after challenge with UDA. Here, we identified changes in specific functional categories, indicating that a significant number of genes respond to the stress induced by UDA, including those that determine fungal virulence. Moreover, the data generated provide evidence that UDA induces AS of several genes involved in diverse metabolic pathways.

## Results

### Transcriptional profile of *T. rubrum* in response to UDA

High-throughput RNA-seq was performed to quantify changes in the transcriptome of *T. rubrum* after treatment with sub-inhibitory doses of UDA. More than 340 million reads were sequenced, corresponding to nine libraries and consisting of 50 or 100 base pairs (bp) each among paired-end or single-end sequences, respectively. Among the libraries, about 50% to 85% of the total high-quality reads aligned to the reference genome of *T. rubrum*, available from the Broad Institute, using the Bowtie tool^22^ (Supplementary Table 1). We reached coverage of 99.9% of the 8,616 annotated genes in the *T. rubrum* genome by considering the genes with at least one count read.

Expression levels of genes were analysed at different time points via pairwise comparisons of the log_2_ ratio of the read count at each time point versus the normalized median read count value for each gene. These data were submitted to an independent filter^23^ and were subsequently assessed based on their false discovery rates (FDRs). From this analysis, 5,168 genes were determined to be modulated in response to UDA at the 3 h time point, among which 2,852 genes were up-regulated and 2,316 genes were down-regulated compared to their expression levels at 0 h (control). In addition, 5,169 genes were modulated in response to UDA at the 12 h time point, with 2,573 genes up-regulated and 2,596 genes down-regulated compared to levels at 0 h (control). The distribution of these differentially expressed genes is shown in Supplementary Figure S1.

**Figure 1:**
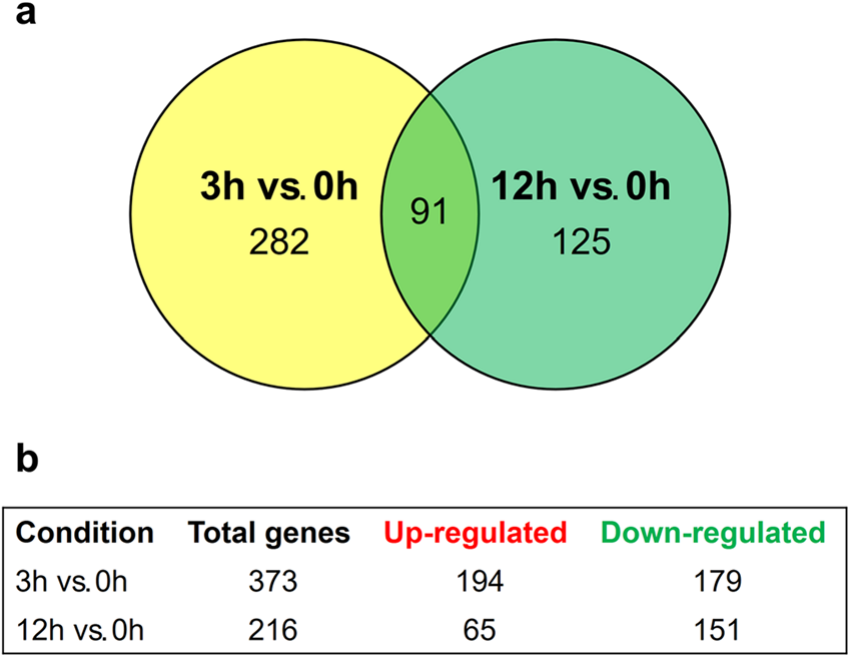
Distribution of genes modulated in response to UDA. (**a**) Venn diagram showing the number of transcripts modulated in *T. rubrum* after exposure to UDA for 3 h or 12 h compared to levels at 0 h (**b**) Number of genes up-regulated and down-regulated at each time point.

Using a cut-off threshold of at least 2.8-fold difference and a statistical significance threshold of *P* < 0.05 yielded a subset of 498 genes that were modulated in response to UDA and distributed as shown in Fig. 1. This procedure minimized the occurrence of false positives due to genes with low expression. Among the 373 genes modulated in response to UDA at 3 h compared to 0 h, 194 and 179 genes were up- and down-regulated, respectively, whereas among the 216 genes modulated at 12 h compared to 0 h, 65 and 151 genes were up- and down-regulated, respectively. We also observed that 282 and 125 genes were exclusively modulated at the 3 h or at the 12 h time point, respectively, compared to the 0 h time point. Furthermore, 91 genes responded to UDA in a time-independent manner, represented by the intersection of the Venn diagram (Fig. 1). For each condition analysed, was generated a list of differentially expressed genes, which is presented in Supplementary Table S2.

**Figure 2:**
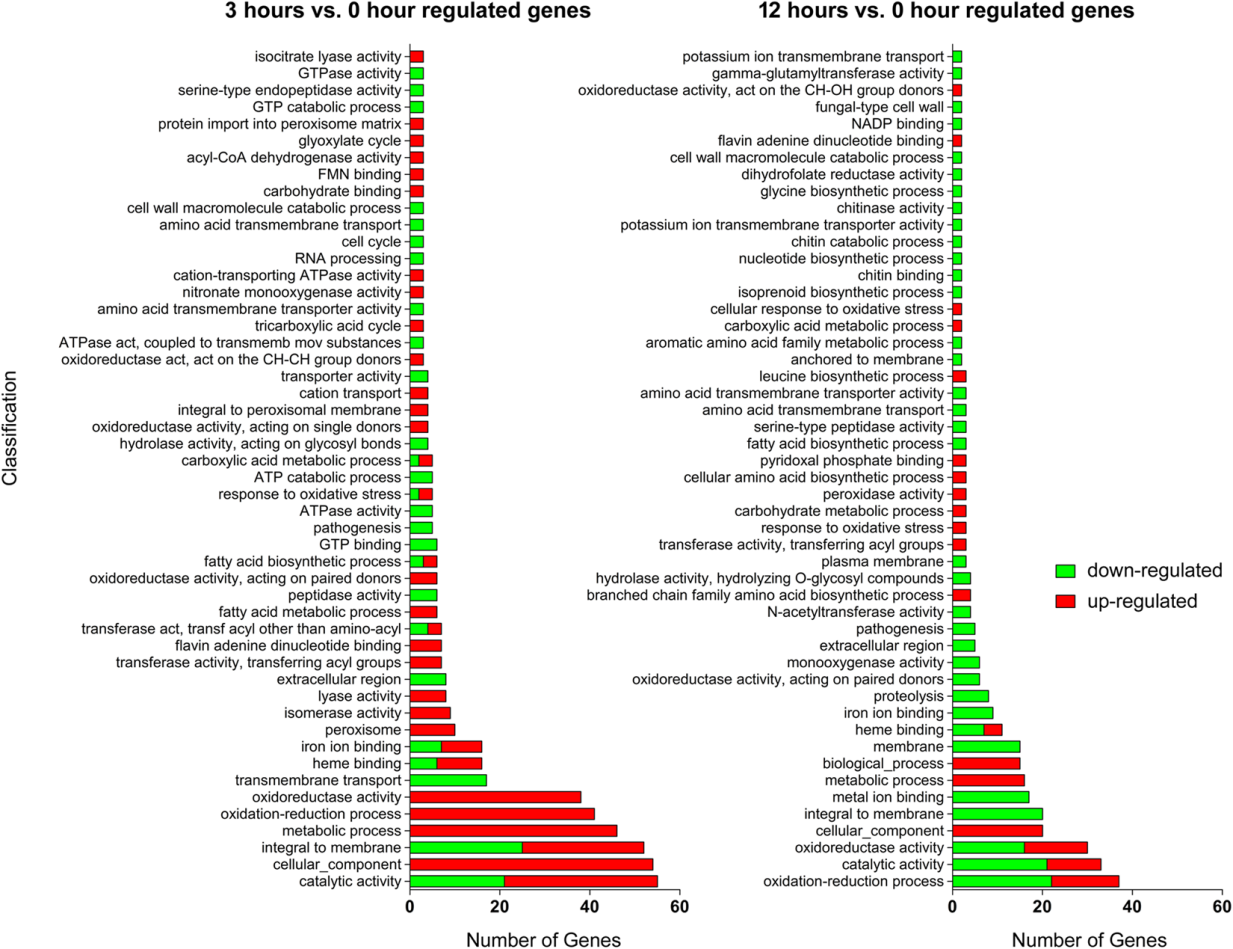
Functional annotation of the most representative genes modulated by *T*. *rubrum* in response to UDA based on Gene Ontology. Green and red bars indicate down- and up-regulated genes, respectively. The categories with more associated terms are displayed in the bottom position of the figure, and are mainly related to metabolic processes, oxidative stress status, fatty acid metabolism, and proteolysis.

**Figure 3:**
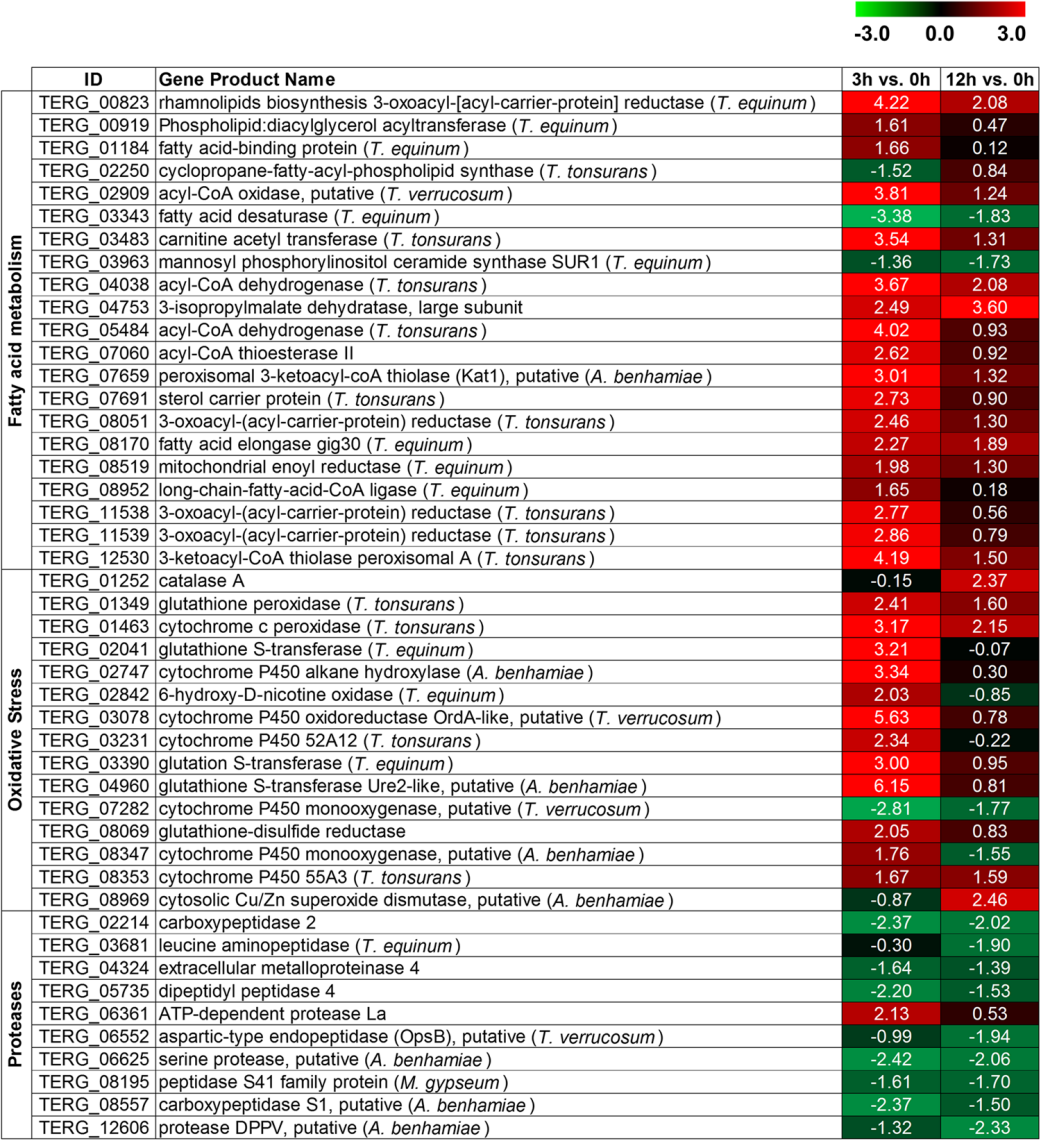
Set of genes belonging to main representative categories modulated after exposure of *T. rubrum* to UDA (log_2_-fold change). Values are colored from green (down-regulated) to red (up-regulated) according to the colour scale.

The functional categorization of differentially expressed genes helps to place them into a physiological context, which improves our understanding of the molecular mechanisms responsive to UDA. Considering the physiological context, we examined the functional distributions of the modulated genes using the Blast2GO and BayGO software programs. The categories of genes that were differentially expressed at 3 h vs. 0 h, and 12 h vs. 0 h are shown in Fig. 2, with only the over-represented genes shown. This analysis indicated that genes modulated by UDA are involved in several cellular processes, such as oxidative stress, transmembrane and peroxisome transport, secondary metabolism, amino acid and lipid metabolism, proteolysis, and pathogenicity, among others. At 3 h (vs. 0 h), a large number of genes related to oxidation-reduction and fatty acid metabolism, oxidoreductase activity, cellular component, and peroxisome transport were up-regulated. At 12 h (vs. 0 h), a large number of genes related to membrane structure, metal ion binding, proteolysis, iron ion binding, pathogenesis, and extracellular events, among others, were down-regulated. This functional enrichment analysis highlighted, in general, three relevant categories involved in UDA response: proteases, fatty acid metabolism, and oxidative stress. Figure 3 provides a list of the genes belonging to these three categories.

**Figure 4:**
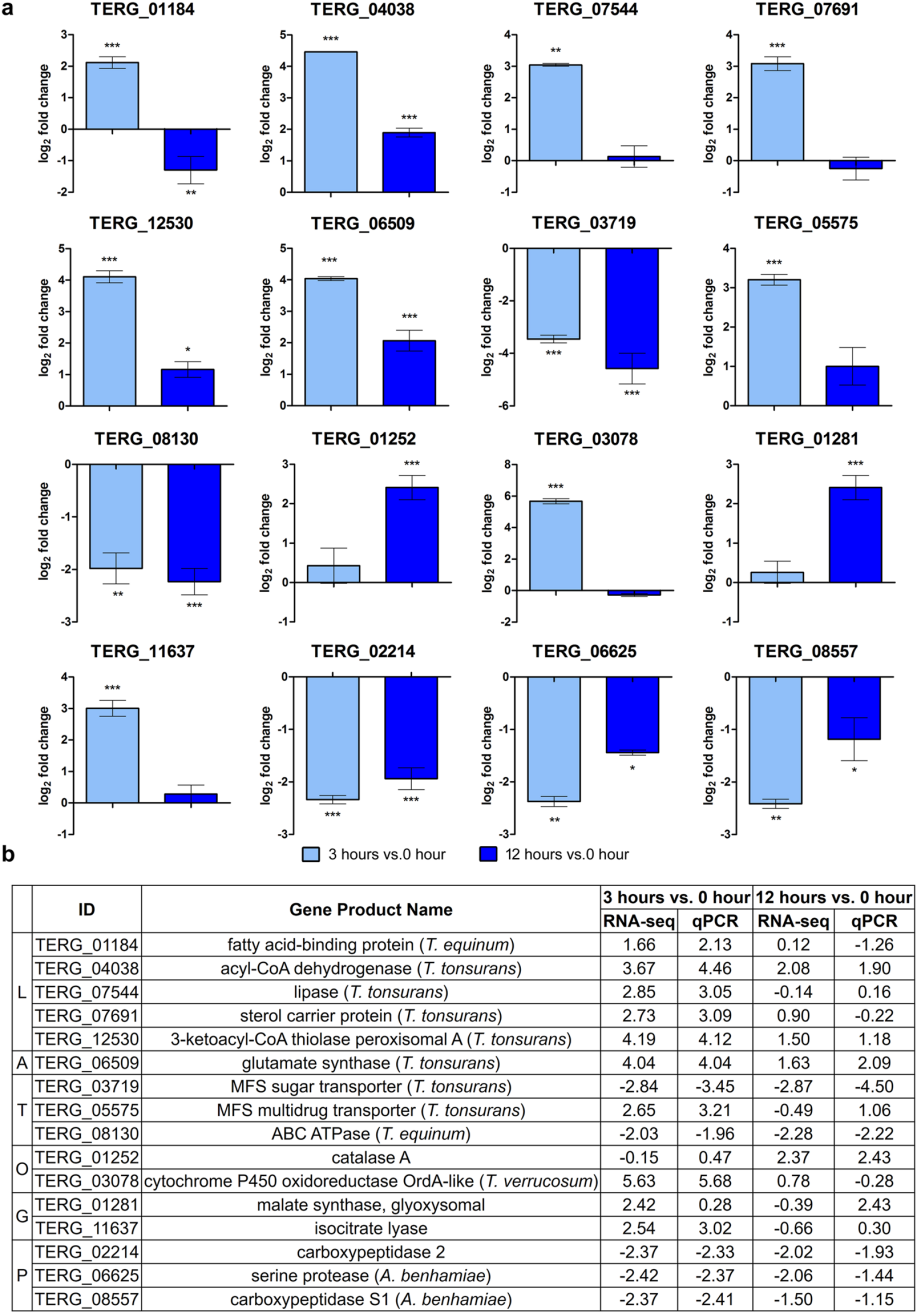
Validation of differentially expressed genes using qRT-PCR. Sixteen genes modulated in response to UDA were amplified in *T. rubrum* mycelia after exposure to UDA for 3 and 12 h. (**a**) Gene expression levels are represented as log_2_-fold change at each time point relative to the control (0 h), as determined using ANOVA followed by Tukey’s *post hoc* test (*P < 0.05; **P < 0.01; ***P < 0.001). (**b**) Comparison of the gene expression levels assessed by RNA sequencing and qRT-PCR. The genes are grouped by categories, in which letters correspond to: (L) lipid metabolism, (A) amino acid metabolism, (T) transport, (O) response to oxidative stress, (G) glyoxylate cycle, and (P) proteolysis and pathogenesis.

To validate the RNA-seq results, we performed qRT-PCR experiments for 16 genes involved in lipid metabolism, amino acid metabolism, transport, response to oxidative stress, glyoxylate cycle, proteolysis and pathogenesis. These results are shown in Fig. 4. Pearson’s correlation analysis indicated that the correlation between the RNA-Seq and qRT-PCR results was high and statistically significant (Pearson’s correlation, *r* > 0.93, *P* < 0.001).

### Plasma membrane damage and cell wall impairment following UDA exposure

The RNA-seq data showed that UDA exposure leads to changes in fatty acid and ergosterol metabolism. For this reason, we investigated the effect of UDA on cellular membrane damage, and consequently cell wall impairment was estimated with a protoplast regeneration assay (Table 1) and determination of ergosterol content. Antifungal compounds that act on the ergosterol biosynthetic pathway, such as ketoconazole (KTC) and terbinafine (TRB), were used as positive controls for both assays. Notably, compounds that act on the cellular membrane impair cell wall regeneration^24^.

**Table 1:** Percent reduction in the number of regenerated protoplasts in the presence of sub-inhibitory doses of antifungals assessed in *T. rubrum*. (*) Values followed by different superscript letters differ significantly (*P* < 0.05) between antifungal treatments. MIC: minimum inhibitory concentration.

Drugs concentration	Reduction of regenerated colonies (%)
0.5 MIC of terbinafine	100^a^
0.25 MIC of terbinafine	100^a^
0.5 MIC of ketoconazole	17.98 ± 1.01^c^
0.25 MIC of ketoconazole	7.56 ± 4.68^d^
0.5 MIC of undecanoic acid	98.56 ± 0.73^a^
0.25 MIC of undecanoic acid	43.86 ± 3.84^b^

Compared to the number of regenerated colonies in the control samples, UDA and TRB resulted in marked inhibition of protoplast regeneration when tested at half the minimum inhibitory concentration (MIC). A decrease in the UDA concentration led to an increase in protoplast regeneration, showing a dose-dependent effect. There was no difference in the number of regenerated colonies under KTC treatment compared to the control, although the growth rate was considerably affected by KTC treatment (data not shown).

Furthermore, ergosterol content was assessed, revealing that UDA led to a marked reduction in ergosterol when compared to that of the control samples. A similar ergosterol reduction was observed under KTC treatment. In contrast, TRB treatment led to a complete inhibition of ergosterol (Table 2).

**Table 2:** Ergosterol levels assessed in *T. rubrum* exposed to antifungals. (*) Values followed by different superscript letters differ significantly (*P* < 0.05) between antifungal treatments.

Antifungals	Ergosterol reduction (%)
Terbinafine	100^a^
Ketoconazole	65 ± 5.2^b^
Undecanic acid	62.6 ± 11.1^b^

### Keratinolytic protease activity

To better understand the impact of UDA on keratinase activity, a keratinolytic assay was conducted and revealed a significant increase in the activity of keratinolytic proteases in *T. rubrum* grown on keratin supplemented with UDA (400 ± 15.48 units/g) compared to the activity in *T. rubrum* grown on keratin alone (99 ± 7.83 units/g).

### Analysis of *T. rubrum* in response to UDA and the oxidizing agent Menadione

**Figure 5:**
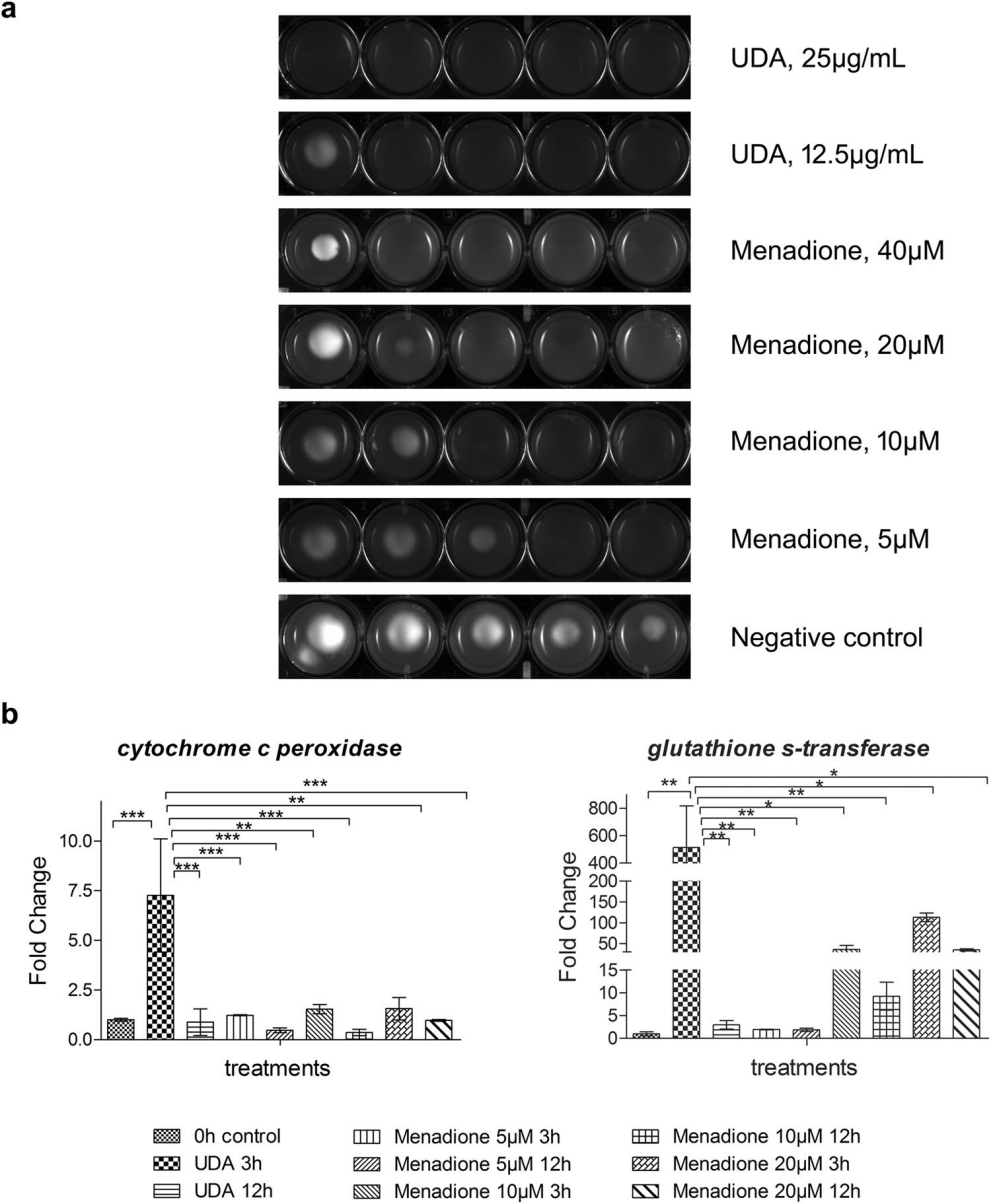
Effect of the oxidizing agent menadione and the fatty acid UDA on *T. rubrum*. (**a**) Susceptibility of *T. rubrum* to menadione (5 µM, 10 µM, 20 µM, 40 µM) and UDA (12.5 µg/mL and 25 µg/mL). Plates were inoculated, from left to right in each panel, with 10^6^, 10^5^, 10^4^, 10^3^, and 10^2^ cells/mL of conidial suspension and incubated at 28 °C for 96 h. (**b**) qRT-PCR for *T. rubrum* genes encoding the oxidizing enzymes glutathione transferase, and cytochrome *c* peroxidase. Relative expression was assessed using 0 h as the reference sample after normalization with the *rpb2* endogenous gene. Significantly different values are indicated by asterisks, as determined using ANOVA followed by Tukey’s *post hoc* test (*P* < 0.05).

The transcriptome data suggested that exposure to UDA leads to an oxidative stress status, with the up-regulation of antioxidant enzymes in *T. rubrum*. Further, we carried out a sensitive phenotype assay and the gene expression analysis of oxidating enzymes. After that, we compared the *T. rubrum* response to UDA with the superoxide-generating compound menadione. For the phenotype assay, we inoculated drops of conidia suspension of *T. rubrum* on Sabouraud medium plates containing oxidizing agent menadione (in ranges of 5 µM to 40 µM), or UDA (11.25 µg/mL or 25 µg/mL). The colony growth was markedly reduced by UDA in both concentrations tested. The colony growth inhibition was closely similar to the highest menadione concentration assayed (Fig. 5). The gene expression analysis of the oxidizing enzymes, glutathione transferase (TERG_04960) and cytochrome c peroxidase (TERG_01463), highlighted their up-regulation after 3 h of UDA exposure. Alike, the menadione exposure promoted the up-regulation of the genes assessed mainly after 3 h of exposure (Fig. 5).

### AS analysis

**Figure 6:**
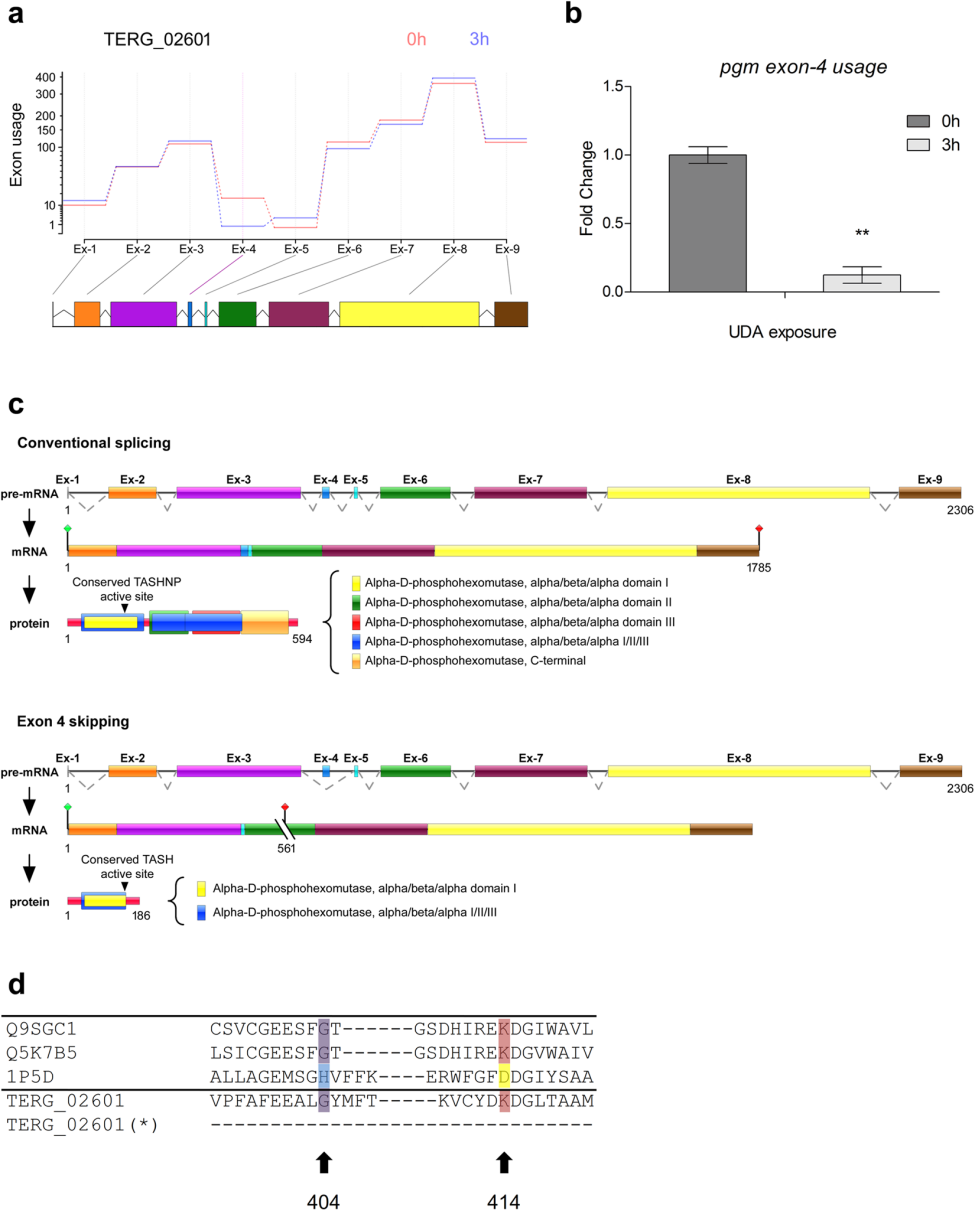
Schematic representation of differential exon 4 usage in *pgm* gene in *T. rubrum*. (**a**) Estimated effects of UDA exposure to 3 h compared to control (0 h) on differential exon usage in the *pgm* gene in *T*. *rubrum*. Exons are shown in coloured boxes and are linked to exon usage diagram by gray lines for no exon-level changes, and the purple line in the fourth exon indicates its differential exon usage. (**b**) qRT-PCR of *T. rubrum* full-length *pgm* gene (exon 4 usage) after UDA exposure for 0 h and 3 h. Significantly different value is shown by asterisks (*P* < 0.05). (**c**) Schematic representation of exon 4 skipping, showing the genomic DNA and the mRNA organization of the *pgm* gene in *T. rubrum* during conventional splicing event, and alternative splicing promoted by exon 4 skipping. Exons are shown as coloured boxes; introns are shown as solid lines. Dotted lines indicate the joining of exonic regions by removal of introns from the sequence. A premature stop codon is indicated by cut lines followed by a red signal. The active site is indicated in residue 148, and is related to the conserved motif TASHNP in “conventional splicing” or TASH in “alternative splicing”. (**d**) Amino acid alignment of a select region of PGM proteins from plants, fungi, bacteria, and *T. rubrum* (Q9SGC1: *Arabidopsis thaliana*, Q5K7B5: *Cryptococcus neoformans*, 1P5D: *Pseudomonas aeruginosa*, and TERG_02601: *T. rubrum*). Conserved residues corresponding to histidine (cyan), glycine (purple) and Lys/Arg (red for lysine, yellow for arginine) are shown. *T*. *rubrum* PGM possesses a glycine in the position corresponding to 404 and a lysine at 414, and these regions are lost in the PGM after alternative splicing (*).

In addition to the identification of differentially expressed genes, we aimed to determine whether exposure to UDA could generate changes in the transcriptome in terms of exon usage and intron retention. According to our analysis of the differential use of exons and based on a statistical significance threshold of FDR < 0.05, a total of 513 exons distributed among 312 genes exhibited potential for differential use at 3 h after UDA exposure (vs. 0 h), and a total of 309 exons among 214 genes were potentially differentially used at 12 h (vs. 0 h). A list of genes exhibiting exon skipping is provided in Supplementary Table S3. Notably, the gene encoding phosphoglucomutase (PGM; TERG_02601) showed marked differential exon usage at 3 h compared to usage at 0 h. The levels of *pgm* exon 4 usage was assessed by qRT-PCR, and showed a considerable decrease after 3 h of UDA exposure in comparison to 0 h (no exposure) (Fig. 6). The PGM protein is generally characterized as showing four domains of alpha D-phosphohexomutase (I-IV) in a superimposed arrangement, with catalytic enzyme cleft formed by four binding loops from each domain. As result of exon skipping events, three of these domains (II, III, and IV or C-terminal) are absent as well as three binding sites loops related to sugar, metal, and phosphate binding loops. Figure 6 presents the differentially used exons following UDA exposure and also describes features of these AS events.

**Figure 7:**
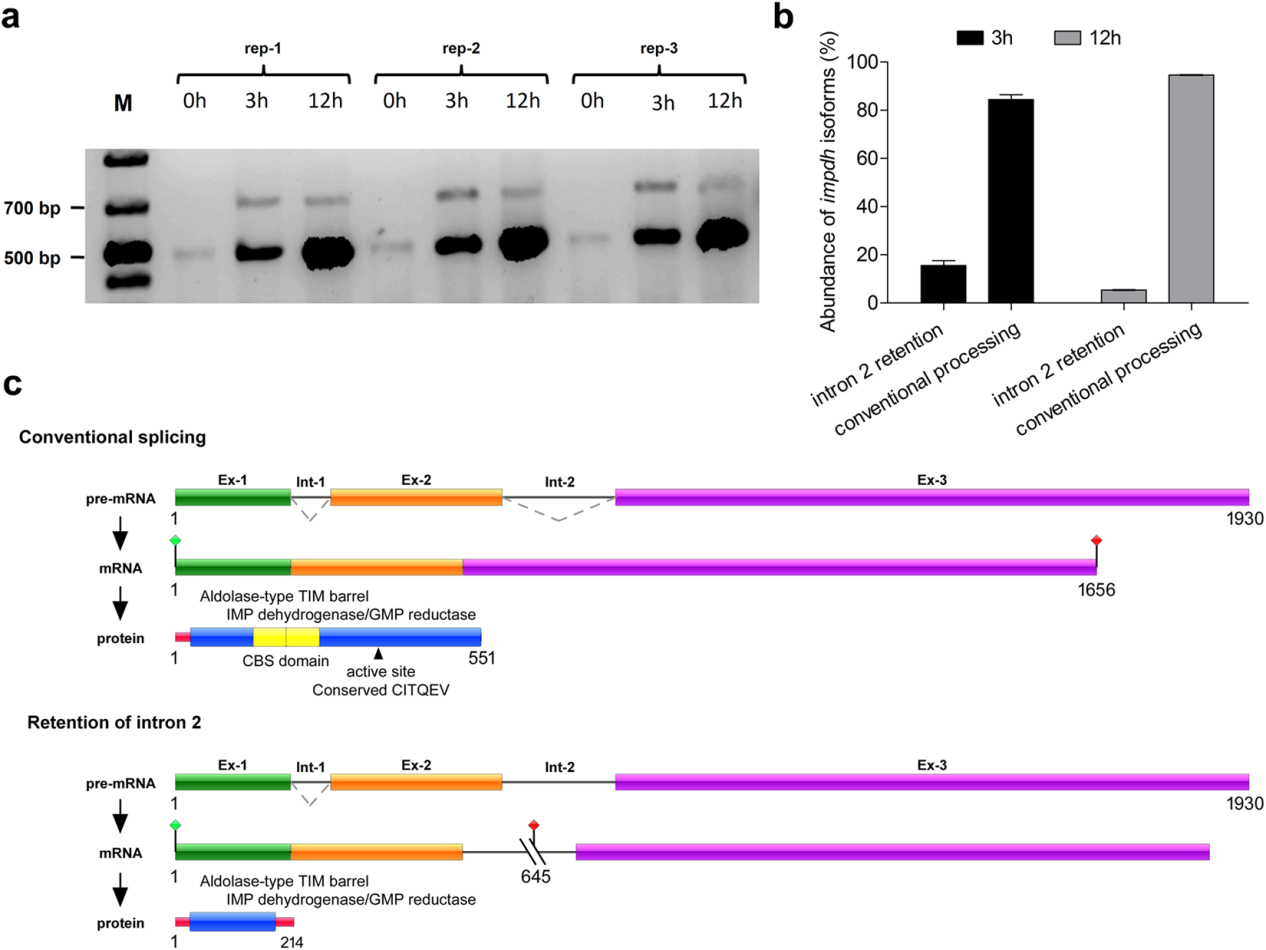
Schematic representation of intron 2 retention in *impdh* gene in *T. rubrum*. (**a**) Retention of intron 2 during the pre-mRNA processing of the *impdh* gene as visualized by RT-PCR of *T. rubrum* exposed to UDA for 0 h (control), 3 h, and 12 h, assessed in three biological replicates. (M) Molecular weight ladder. The expected size of each amplicon was 481 bp for the elimination of intron 2 and 684 bp for the retention of intron 2. The full-length gel is presented in Supplementary Figure S3 (**b**) The PCR products were visualized in ImageJ software and subjected to densitometry analysis for determining the abundance for amplicons with retention of intron 2, and amplicons for conventional processing. The ratio of intron-2 retention corresponds to approximately 15:100 and 5:100 for 3 h and 12 h of UDA exposure, respectively. (**c**) Schematic representation of intron 2 retention, showing genomic DNA and the mRNA organization of the *impdh* gene of *T. rubrum* during conventional splicing, and alternative splicing promoted by intron 2 retention. Exons are shown as coloured boxes; introns are shown as solid lines. Dotted lines indicate the joining of exonic regions by the removal of introns from the sequence. A premature stop codon is indicated by cut lines followed by a red signal. The active site is indicated in residue 355.

In terms of intron retention, the reads contained 290 introns distributed among 216 genes at 0 h, 165 introns among 127 genes at 3 h, and 879 introns among 663 genes at 12 h after UDA exposure (Supplementary Table S4). Supplementary Figure S2 shows a histogram of intron read counts for each experimental condition. At 0 h, the read count for the majority of introns was between 1,000 and 10,000 reads, which was the same as that at 3 h. At 12 h, the majority of introns exhibited read counts of between 100 and 1,000 reads. These data suggest that at 12 h, there were fewer reads per intron but a higher number of introns retained. Among the genes with retained introns, the gene encoding inosine monophosphate dehydrogenase (IMPDH; TERG_06846), which is involved in purine biosynthesis, was noteworthy due to the high levels of retention of intron 2, for which 9,285 and 3,792 reads were obtained at 3 h and 12 h, respectively. For this reason, AS in this gene was confirmed by RT-PCR (Fig. 7). In addition, a schematic representation of gene and protein under both conventional processing and retention of intron 2 retention was analysed (Fig. 7). Following intron retention, the protein coding frame was altered, generating a short protein with interrupted functional domains.

## Discussion

The antifungal and antibacterial properties of fatty acids have long been described. UDA exposure is assumed to lead to a breakdown in phospholipid synthesis, which in turn cause changes in membrane composition, promoting membrane damage and partial growth inhibition^25^. However, the fundamental aspects of the physiology of *T. rubrum* in response to exposure to UDA are not well known. To this purpose, our data revealed new insights into the mechanisms with which *T. rubrum* attempts to overcome UDA exposure, showing three main categories of genes affected by UDA: lipid metabolism and cellular membrane composition, oxidative stress, and pathogenesis with a focus on protease secretion.

To ameliorate the toxicity induced by fatty acids, the cell utilizes certain mechanisms to incorporate the exogenous fatty acids into pathways tied to phospholipid biosynthesis or even to the degradation of exogenous fatty acids^26^. As the fatty acids enter the cell, they are transported by acyl careers in their activated form, acetyl-CoA, which is in turn a substrate for phospholipid synthesis and beta-oxidation and is linked to metabolic pathways such as the glyoxylate and the tricarboxylic acid (TCA) cycles. The attempt to use UDA for phospholipid synthesis was observed in our data through the up-regulation of sterol carrier protein (TERG_07691), followed by the induction of genes related to fatty acid synthesis and the elongation of phospholipids, diacylglycerol acyltransferase (TERG_00919), long-chain-fatty-acid-CoA ligase (TERG_08952), and fatty acid elongase gig30 (TERG_08170) especially after 3 h of UDA exposure.

Additionally, the degradation of fatty acids is another defence mechanism against toxicity. Beta-oxidation is carried out inside the peroxisome through the action of enzymes such as acyl-oxidases and ketoacyl thiolase^27^ The acetyl CoA generated is a substrate for the TCA cycle and may be transported to mitochondria by acetyl CoA transporters like citrate synthase or acetyl-carnitine. Additionally, the TCA enzyme aconitase converts citrate to isocitrate to feed the glyoxylate cycle, bypassing the TCA cycle^28^. Our data showed the up-regulation of genes involved in these pathways, which are linked by the transport of acetyl CoA across distinct organelles. In general, these metabolic changes occurred after 3 h of UDA exposure. It is worth noting that most lipid metabolic changes occurred after 3 h of UDA exposure, which may be explained by the fact that toxic fatty acids rapidly induce cell growth arrest^29^, requiring a prompt fungal effort to deal with exogenous fatty acids.

Previous reports have shown that in addition to interfering with lipid metabolism, fatty acid poisoning involves the formation of hydroperoxides^30^ and changes in membrane fluidity and permeability. In this context, the increased activity of beta-oxidation, which serves to degrade UDA, may be responsible for increasing the generation of H_2_O_2_. The abrupt induction of beta-oxidation in a manner linked to the TCA cycle leads to the overproduction of toxic reactive oxygen species (ROS); this probably exceeds the cell’s ability to deal with the deleterious effects of ROS, promoting oxidative stress. Similarly, it has been shown that treatment with antifungals like amphotericin B, ciclopirox, and micafungin trigger oxidative damage. Furthermore, the fungicidal properties of these different classes of antifungals are associated with the induction of typical cascades of the oxidative damage cellular pathway^31^.

Moreover, genes of the ergosterol pathway, *erg3*, *erg4*, and *erg26*, were down-regulated after UDA exposure. Interestingly, the proteins encoded by these genes act upstream of methyl-sterols in the ergosterol pathway, and their repression, in particular for genes *erg3* and *erg 4*, is associated with oxidative stress^32^. A possible reason for this is that membrane composition affects the transmembrane diffusion of H_2_O_2_, a chemical catalyst with oxidizing properties^33^. Indeed, the assays carried out to determine the effect of UDA exposure on the ergosterol content and damage to the cell membrane demonstrated that levels of ergosterol are profoundly reduced by UDA, similar to what was observed with the positive control, KTC. UDA also impaired protoplast regeneration, which is related to cell membrane damage. It is likely that these cell membrane effects favour the scavenging of H_2_O_2_, since changes in the lipid composition of cell membranes represent one way to regulate levels of H_2_O_2_ within the cell. Therefore, we propose that UDA fungitoxic effects are mainly due to impairments in energy pathways that indirectly trigger oxidative stress, secondarily leading to cellular membrane damage.

Free radicals are produced in the mitochondria under physiological conditions, as natural by-products of the respiratory chain. Free radicals have roles in signalling, metabolic adaptation, and immunity^34^. However, an imbalance in the endogenous concentration of ROS, promoted by external stimuli, can lead to alterations in macromolecules such as proteins, lipids, and DNA, which eventually lead to cell damage and death. In this context, adaptation to oxidative stress is an essential strategy for fungal pathogenicity and survival^35^. Fungi can cope with ROS by applying enzymatic and non-enzymatic defences^36^. The present data showed that exposure to UDA leads to the up-regulation of antioxidant enzymes in *T. rubrum*, such as superoxide dismutase (TERG_08969, an orthologue of SOD5 in *Candida albicans*), catalases and peroxidases (TERG_01252 and TERG_01463), glutathione transferases (TERG_04960, TERG_02041, TERG_03390), and GSH peroxidases (TERG_01349). Corroborating these data, similar enzymatic oxidative detoxification is observed in *Aspergillus fumigatus* in response to the cellular deleterious effects of amphotericin B^37^. The SOD defence is considered especially important since this enzyme promotes the shunting of superoxides away from lipid hydroperoxides, converting superoxides to other ROS like hydrogen peroxide; this can then be metabolized by catalases and converted to water or molecular oxygen. In the same context, peroxidases and GSH transferases act as metabolizers of organic hydroperoxides^36^.

Furthermore, the sensitive phenotype assay showed a decrease in *T. rubrum* filamentous growth after exposure to UDA and menadione. In addition, the evaluation of gene modulation of glutathione transferase, and cytochrome *c* peroxidase presented their up-regulation mainly during the first hours of drugs exposure. We can associate these results with the first defence strategy employed by fungi against the oxidative stress status, as previously described^38^. Therefore, all these data suggested UDA as a member of a group of drugs defined as “oxidative stress drugs”, showing the cellular antioxidant system as also a target for antifungal action of UDA, as recenttly reported for other antifungal compounds like amphotericin B and itraconazole^39^.

Curiously, transcription factors like SKN7 and Yap1/Cap1, which are responsible for regulating the oxidative response in fungi by acting on targets related to oxidative detoxification and oxidative damage repair, did not show any changes under the conditions assayed.

Our previous knowledge regarding the toxicity of UDA toward dermatophytes also suggests there is a decrease in keratinase secretion^13^. Proteases are key dermatophyte virulence factors, assisting in fungal adherence and infection maintenance and affecting nutrient uptake^40,41^. In this study, we verified the down-regulation of endoproteases (subtilisin and metalloproteases) and exoproteases (aminopeptidase leucine and dipeptidyl peptidases) as well as carboxypeptidases and aspartic proteases. In contrast, the assays used to verify the influence of UDA on the keratinolytic activity of *T. rubrum* demonstrated an increase in keratinolytic activity of up to fourfold following UDA treatment. Although speculative, these findings suggest that an increase in the keratinase activity seems not be related to an overproduction of the secreted enzyme. It might instead be the result of posttranslational modifications, which could affect either the rate of enzymatic catalysis or the catalytic affinity of the enzyme for the substrate^9^. Also, it might correlate to the changes in metabolic events affecting the association with other proteins. Indeed, a previous report hinted that the mutation on the *A. nidulans lipA* gene (*lipA1* allele), which codes for a putative lipase, has a pleiotropic effect on the pattern of posttranslational modifications and secretion of enzymes, concomitantly with the adaptive response to UDA^9^. Moreover, the impact of posttranslational modifications on the activity of other secreted enzymes by fungi has been documented^42–44^.

In addition to assessing changes in gene expression, we also sought to assess pre-mRNA processing events following UDA exposure. According to our data, we found that the gene encoding IMPDH exhibited high retention of intron 2 at 3 h and 12 h following UDA exposure. IMPDH participates in the synthesis of purines, providing ATP and GTP for the cells, which are involved in virtually every energy metabolic pathway. Recently, this enzyme has been described as an attractive antifungal target, as it differs from the human protein^42^. Additionally, IMPDH plays roles in virulence, as previously shown for *Cryptococcus neoformans*^42^. This protein possesses an unusual structural organization, with a pair of cystathionine beta synthase (CBS) subdomains in tandem in the middle of the main dehydrogenase domain. As a consequence of intron 2 retention in the *impdh* gene after UDA exposure for 3 h and 12 h, the IMP dehydrogenase domain was interrupted, and the CBS subdomain was lost. Although the role of the CBS domain has not been fully characterized, it seems to regulate IMPDH activity and balance the levels of GTP and ATP in the cell^43^. Moreover, IMPDH can take on different structures according to the chemical reactions catalysed, participating in interactions with nucleic acids and polyribosomes, and it may also be associated with proteins involved in splicing regulation^44,45^.

Likewise, the gene encoding PGM was notable for its differential exon usage at 3 h post-UDA exposure. PGM is an enzyme involved in gluconeogenesis metabolism that uses oxaloacetate from the TCA cycle as a substrate for the production of glucose. Our data demonstrated a significant difference in exon 4 usage, with log_2_-fold decreases of 2.32 after 3 h of UDA exposure. Previous reports have shown that PGM plays roles in virulence, growth at low pH, and carbon starvation, as well as being related to cellular membrane fatty acid composition^46,47^. In this context, as previously described^48^, the suppression of *N*-acetyl phosphoglucosamine mutase (AGM), which is similar to PGM, leads to changes in cell wall composition, decreasing chitin and GlcNac and increasing mannose and glucans. The resulting shifts in cell wall composition possibly trigger the cell wall integrity pathway (CWI). Hence, PGM function is regulated by acetyl-CoA flows, which are provided by the TCA cycle and fatty acid beta-oxidation, whereas PGM activity is associated with cell wall remodelling, with changes in function possibly orchestrating the signalling cascade for cell wall repair. Here, we showed that exon 4 skipping leads to a protein length of 186 aa, removing almost one-third of the parental protein. The resulting enzyme, which originally contained four phosphohexomutase domains, maintains only the first of these domains following this AS event. Additionally, the Gly404 and Lys414 residues that are highly conserved among the PGM subfamily are lost. The Lys414 residue is located close to the active-site cleft of the enzyme, near the substrate binding site, and is highly associated with proper PGM catalytic function^49^. Moreover, the metal, sugar, and phosphate-binding loops located in domains II, III, and IV, respectively, are absent from the protein after exon skipping^50^. Overall, the AS events described here for PGM and IMPDH are associated with impairments in protein function and reflects a tight regulation between fungal energy metabolism and cellular signalling involved in the UDA response.

Thus, our interpretation of the data regarding the mechanisms involved in UDA detoxification in *T. rubrum* implicates the synthesis and degradation of fatty acids, followed by an imbalance in ROS production, which leads to changes in cellular membrane composition (ergosterol content) and keratinolytic activity. These are accompanied by strict pre-mRNA processing regulation.

Taken together all these results provide insights into UDA fungitoxicity toward dermatophytes and shed lights on new therapeutic targets. On the other hand, we are aware of the limitations concerning to RNA-seq technology, and the need to assess other cellular profiles to broaden the knowledge of the complex metabolic interactions in fungal responses against the stress promoted by antifungal compounds.

## Materials and Methods

### Strains and culture conditions

*T. rubrum* strain CBS 118892, obtained from the *Centraalbureau Voor Schimmelcultures* in the Netherlands, was cultured on malt extract agar (MEA) for 17 d at 28 °C to produces conidiation. Approximately 1 × 10^6^ conidia/mL, prepared as previously described^51^, was inoculated in Sabouraud medium and cultured at 28 °C for 96 h under constant agitation. Mycelia were aseptically transferred to RPMI 1640 media (Gibco, USA) in the absence or presence of 17.5 μg/mL UDA (Sigma, USA), which corresponds to 70% of its MIC. Mycelia were then incubated for 3 or 12 h at 28 °C under constant agitation and subsequently stored at −80 °C until RNA extraction. UDA susceptibility was evaluated in *T. rubrum* by assessing the MIC using the microdilution approach (M38-A) proposed by the Clinical and Laboratory Standards Institute (CLSI) as previously described^52^, which was found to be 25.0 μg/mL. The concentrations tested were serial dilutions of UDA in ethanol ranging from 1.56 μg/mL to 200 μg/mL. All assays were carried out in triplicate at 28 °C for 5 days.

### RNA extraction

Total RNA was extracted from frozen mycelia (approx. 100 mg) using the Illustra RNAspin Mini Isolation Kit (GE Healthcare, USA) and treated with RNAse-free DNAse I (Invitrogen, USA), according to the manufacturers’ guidelines. We used a NanoDrop ND-1000 spectrophotometer to estimate the concentration of RNA. RNA quality was verified using an Agilent 2100 Bioanalyzer (Agilent, USA). RNA samples were maintained at −80 °C until sequencing.

### cDNA library construction and high-throughput sequencing

Three independent RNA biological replicates for each condition were used for the synthesis of cDNA with the TruSeq RNA library Kit (Illumina, USA). The protocol consisted of poly A-selected RNA extraction, RNA fragmentation, and reverse transcription using random hexamer primers. The libraries were sequenced on Illumina HiSeq. 2000 system (Illumina, USA) using the PCR-synthesis methodology to generate 100 or 50 bp single-end and paired-end reads, respectively. The Eurofins Company constructed the libraries and performed the sequencing reactions.

### Data analysis

The reads were quality filtered using the software FastQC, aligned with the *T. rubrum* genome available at the Broad Institute’s Dermatophyte Comparative Database (ftp://ftp.broadinstitute.org/pub/annotation/fungi) using the Bowtie2 algorithm^22^, and inspected with the software Integrative Genomics Viewer (IGV)^53,54^. Further, the independent filter, which precedes the statistical filter was applied to identify gene by gene variances among biological replicates, followed by FDR^23^. These analyses were performed using the DESeq package and manipulated in the R statistical environment^55^. Genes with FDRs < 0.05 were considered differentially expressed and are presented in Volcano plots. Those genes showing a log_2_-fold change between −1.5 and +1.5 were functionally categorized using Gene Ontology (GO) terms assigned using the Blast2GO algorithm^53,54^. Highly represented categories under each condition were determined by enrichment analysis using the BayGO algorithm^56^.

A transcriptome-wide survey of AS in differentially expressed genes after exposure of *T*. *rubrum* to UDA was conducted using analysis of exon skipping and intron retention. The differential use of exons was analysed in libraries using the HTSeq^57^ and DEXSeq^58^ packages to assess read counts in the exonic regions of each gene and normalize the data, respectively. FDRs were calculated and significance was determined by an adjusted *P* < 0.05.

Intron retention analysis was performed through *ad hoc* Perl scripts, using only the single-end libraries. Introns were identified in the reference genome and aligned against the sequenced libraries, and read counts were analysed for each retained intron. RT-PCR was used to confirm the properties of genes of interest. Primers are listed in Supplementary Table S5.

### Data Availability

RNA-seq data are deposited in the GEO (Gene Expression Omnibus)^59^ database under accession number GSE102872, on the web site: https://www.ncbi.nlm.nih.gov/geo/query/acc.cgi?acc=GSE102872.

### qRT-PCR analysis

Gene expression was quantified by qPCR with the StepOnePlus Real-Time PCR system (Applied Biosystems, US). Independent samples from each time point were used, with biological and technical replication performed in triplicate. Specific primer pairs were designed using the Gene Runner Software, with specificity confirmed by BLAST. Primers used are listed in Supplementary Table S3. Each qPCR reaction was performed in a final volume of 12.5 μL, containing 1 μL primer, 6.25 μL SYBR Green PCR Master Mix (Applied Biosystems, UK), and 50 ng cDNA. The cycling conditions included an initial PCR step of 95 °C for 10 min, followed by 40 cycles of 95 °C for 15 s and 60 °C for 1 min. Expression was assessed based on the relative quantification of responsive genes using the 2^−∆∆Ct^ method^60^. Data were normalized with two endogenous controls, DNA-dependent RNA polymerase II (rpb II) and DNA topoisomerase II^51^. The reference sample was taken at 0 h. Statistical analysis was performed using one-way ANOVA with Tukey’s *post hoc* test using Graph Pad Prism v. 5.1 software. Significance was indicated by *P* < 0.05.

### Protoplast regeneration assay

Protoplast regeneration assays were carried out as previously described^24^. Mycelia of *T. rubrum* were grown for 6 days in Sabouraud media, harvested, and inoculated in 30 mL of a solution containing lysing enzymes from *Trichoderma harzianum* (Sigma-Aldrich, USA), pH 6.8, followed by incubation for approximately 5 h at 28 °C under gentle agitation. Then, the protoplasts were filtered using glass wool, collected by centrifugation (1556 × *g*), and washed with 10 mL of regeneration buffer (0.8 M NaCl, 10 mM CaCl_2_, 50 mM Tris-HCl, pH 7.5), and the concentration of the cells was adjusted to 1 × 10^5^ protoplasts/mL. To evaluate the impact of the drug on the cellular membrane and cell wall, regenerated protoplasts were selected on solid minimal medium^61^, which was supplemented with 1 M sucrose, 0.2% casein, and 0.07 mM NaNO_3_. UDA, KTC, and TRB at 0.5 MIC and 0.25 MIC were added, followed by incubation for 7 days at 28 °C. Culture media without drugs was used for the control. The MIC value for each drug was 25 µg/mL UDA, 0.06 µg/mL KTC, and 0.02 µg/mL TRB.

### Ergosterol dosage

*T*. *rubrum* mycelia were incubated in 50 mL liquid Sabouraud medium for 24 h at 28 °C with agitation (200 rpm). Mycelia were harvested by filtration and inoculated into 20 mL Sabouraud medium containing 2× MIC of UDA. KTC and TRB were used as controls. Mycelia were incubated for 48 h under the same conditions as described above and harvested by filtration. Ergosterol extraction was carried out as previously described^62^, and reductions in ergosterol were measured and calculated as percent differences between test and control samples.

### Keratinolytic assay

Keratinolytic activity was assayed as previously described^63^ with modifications. Keratin was used as the substrate, with 1.0 mL of culture medium as the enzyme. The test was carried out at pH 8.0. Conidia (5 × 10^5^) were inoculated into 125-mL Erlenmeyer flasks containing 25 mL of water supplemented with keratin powder (2.5 g/L) at pH 5.0 as the sole carbon and nitrogen source. These were incubated under shaking (120 rpm/min) for 7 days at 28 °C. UDA was added to flasks in sublethal doses (MIC_50_) and maintained under the same conditions described above. The culture media was collected by filtration, and keratinolytic activity was determined. Mycelium-specific activities were expressed as units per gram of mycelium dry weight. The resulting data represent the mean values of three determinations.

### Oxidative stress and gene expression analysis

A serial drop dilution assay was carried out to analyze the effect of UDA, and menadione on *T*. *rubrum* growth. Plates were innoculated with different concentrations of a conidia suspension in a range of 10^6^ to 10^2^ cell/mL. After 96 h of incubation at 28 °C, the imagens were taken using ImageJ software^64,65^. For gene analysis assay, about 1 × 10^6^ conidia were inoculated into 100 mL of Sabouraud medium and incubated at 28 °C, under shaking for 96 h. The resulting mycelia were transferred to Sabouraud medium without drugs (control) or in the presence of different menadione concentrations (5 µM, 10 µM, 20 µM), or UDA (17.5 μg/mL). Then the mycelia were incubated for 3 or 12 h at 28 °C, under constant agitation. Then, the gene expression analysis of the encoding gene of Gluthatione transferase, and Cytochrome c peroxidase were assessed using *rpb2* as normalizer, and 2^−∆∆Ct^ method. Statistical analysis was performed using one-way ANOVA with Tukey’s *post hoc* test (*P* < 0.05).

## Electronic supplementary material

Supplementary Figures and Tables

## References

[1] Peres NTA, Maranhão FC, Rossi A, Martinez-Rossi NM (2010). Dermatophytes: host-pathogen interaction and antifungal resistance. An Bras Dermatol.

[2] Martinez-Rossi NM, Peres NT, Rossi A (2016). Pathogenesis of Dermatophytosis: Sensing the Host Tissue. Mycopathologia.

[3] Nenoff, P., Kruger, C., Ginter-Hanselmayer, G. & Tietz, H. J. Mycology - an update. Part 1: Dermatomycoses: causative agents, epidemiology and pathogenesis. *J Dtsch Dermatol Ges* **12**, 188–209; quiz 210, 188–211; quiz 212 (2014). 10.1111/ddg.12245 24533779

[4] Ameen M (2010). Epidemiology of superficial fungal infections. Clin Dermatol.

[5] Nir-Paz R (2003). Deep infection by *Trichophyton rubrum* in an immunocompromised patient. J Clin Microbiol.

[6] Iwaki T (2008). Multiple functions of ergosterol in the fission yeast Schizosaccharomyces pombe. Microbiology.

[7] Martinez-Rossi NM, Peres NTA, Rossi A (2008). Antifungal resistance mechanisms in dermatophytes. Mycopathologia.

[8] Das SK, Banerjee AB (1982). Effect of undecanoic acid on phospholipid metabolism in *Trichophyton rubrum*. Sabouraudia.

[9] Brito-Madurro AG (2008). A single amino acid substitution in one of the lipases of *Aspergillus nidulans* confers resistance to the antimycotic drug undecanoic acid. Biochemical Genetics.

[10] Vicher EE, Lyon I, White EL (1959). Studies on the respiration of *Trichophyton rubrum*. Mycopathologia.

[11] Brock M, Buckel W (2004). On the mechanism of action of the antifungal agent propionate. Eur J Biochem.

[12] Samson FE, Katz AM, Harris DL (1955). Effects of acetate and other short-chain fatty acids on yeast metabolism. Arch Biochem Biophys.

[13] Das SK, Banerjee AB (1982). Effect of undecanoic acid on the production of exocellular lipolytic and keratinolytic enzymes by undecanoic acid-sensitive and -resistant strains of *Trichophyton rubrum*. Sabouraudia.

[14] Das SK, Banerjee AB (1981). Effect of undecanoic acid on cell permeability and respiration of *Trichophyton rubrum*. Acta Microbiol Pol.

[15] Monod M (2008). Secreted proteases from dermatophytes. Mycopathologia.

[16] Zaugg C (2009). Gene expression profiling in the human pathogenic dermatophyte Trichophyton rubrum during growth on proteins. Eukaryot Cell.

[17] Bitencourt TA (2016). Transcription profile of Trichophyton rubrum conidia grown on keratin reveals the induction of an adhesin-like protein gene with a tandem repeat pattern. BMC Genomics.

[18] Mendes NS, Silva PM, Silva-Rocha R, Martinez-Rossi NM, Rossi A (2016). Pre-mRNA splicing is modulated by antifungal drugs in the filamentous fungus Neurospora crassa. FEBS Open Bio.

[19] Leal J (2009). A splice variant of the *Neurospora crassa hex-1* transcript, which encodes the major protein of the Woronin body, is modulated by extracellular phosphate and pH changes. FEBS Lett.

[20] Burmester A (2011). Comparative and functional genomics provide insights into the pathogenicity of dermatophytic fungi. Genome Biol.

[21] Martinez DA (2012). Comparative genome analysis of Trichophyton rubrum and related dermatophytes reveals candidate genes involved in infection. MBio.

[22] Langmead B, Salzberg SL (2012). Fast gapped-read alignment with Bowtie 2. Nat Methods.

[23] Bourgon R, Gentleman R, Huber W (2010). Independent filtering increases detection power for high-throughput experiments. Proc Natl Acad Sci USA.

[24] Bitencourt TA (2013). Trans-chalcone and quercetin down-regulate fatty acid synthase gene expression and reduce ergosterol content in the human pathogenic dermatophyte Trichophyton rubrum. BMC Complement Altern Med.

[25] Das SK, Banerjee AB (1983). Effect of undecanoic acid on lipid composition of *Trichophyton rubrum*. Mycopathologia.

[26] Parsons JB, Frank MW, Subramanian C, Saenkham P, Rock CO (2011). Metabolic basis for the differential susceptibility of Gram-positive pathogens to fatty acid synthesis inhibitors. Proc Natl Acad Sci USA.

[27] Poirier Y, Antonenkov VD, Glumoff T, Hiltunen JK (2006). Peroxisomal beta-oxidation–a metabolic pathway with multiple functions. Biochim Biophys Acta.

[28] Strijbis K, Distel B (2010). Intracellular acetyl unit transport in fungal carbon metabolism. Eukaryot Cell.

[29] Parsons JB, Yao J, Frank MW, Jackson P, Rock CO (2012). Membrane disruption by antimicrobial fatty acids releases low-molecular-weight proteins from Staphylococcus aureus. J Bacteriol.

[30] Knapp HR, Melly MA (1986). Bactericidal effects of polyunsaturated fatty acids. J Infect Dis.

[31] Belenky P, Camacho D, Collins JJ (2013). Fungicidal drugs induce a common oxidative-damage cellular death pathway. Cell Rep.

[32] Montanes FM, Pascual-Ahuir A, Proft M (2011). Repression of ergosterol biosynthesis is essential for stress resistance and is mediated by the Hog1 MAP kinase and the Mot3 and Rox1 transcription factors. Mol Microbiol.

[33] Sousa-Lopes A, Antunes F, Cyrne L, Marinho HS (2004). Decreased cellular permeability to H2O2 protects Saccharomyces cerevisiae cells in stationary phase against oxidative stress. FEBS Lett.

[34] Bienert GP, Schjoerring JK, Jahn TP (2006). Membrane transport of hydrogen peroxide. Biochim Biophys Acta.

[35] Brown AJ (2014). Stress adaptation in a pathogenic fungus. J Exp Biol.

[36] Missall TA, Lodge JK, McEwen JE (2004). Mechanisms of resistance to oxidative and nitrosative stress: implications for fungal survival in mammalian hosts. Eukaryot Cell.

[37] Gautam P (2008). Proteomic and transcriptomic analysis of Aspergillus fumigatus on exposure to amphotericin B. Antimicrob Agents Chemother.

[38] Li Q, McNeil B, Harvey LM (2008). Adaptive response to oxidative stress in the filamentous fungus Aspergillus niger B1-D. Free Radic Biol Med.

[39] Kim JH, Chan KL, Faria NC, Martins Mde L, Campbell BC (2012). Targeting the oxidative stress response system of fungi with redox-potent chemosensitizing agents. Front Microbiol.

[40] Tainwala R, Sharma Y (2011). Pathogenesis of dermatophytoses. Indian J Dermatol.

[41] Monod M (2002). Secreted proteases from pathogenic fungi. Int J Med Microbiol.

[42] Morrow CA (2012). De novo GTP biosynthesis is critical for virulence of the fungal pathogen Cryptococcus neoformans. PLoS Pathog.

[43] Pimkin M, Markham GD (2008). The CBS subdomain of inosine 5’-monophosphate dehydrogenase regulates purine nucleotide turnover. Mol Microbiol.

[44] Hedstrom L (2009). IMP dehydrogenase: structure, mechanism, and inhibition. Chem Rev.

[45] Butland G (2005). Interaction network containing conserved and essential protein complexes in Escherichia coli. Nature.

[46] Buckley AA, Faustoferri RC, Quivey RG (2014). beta-Phosphoglucomutase contributes to aciduricity in Streptococcus mutans. Microbiology.

[47] Lima Pde S (2014). Transcriptional and proteomic responses to carbon starvation in Paracoccidioides. PLoS Negl Trop Dis.

[48] Fang, W. *et al*. Genetic and structural validation of Aspergillus fumigatus N-acetylphosphoglucosamine mutase as an antifungal target. *Biosci Rep* **33** (2013). 10.1042/BSR20130053 PMC3763426 23844980

[49] Lee Y, Mehra-Chaudhary R, Furdui C, Beamer LJ (2013). Identification of an essential active-site residue in the alpha-D-phosphohexomutase enzyme superfamily. FEBS J.

[50] Nishitani Y (2006). Crystal structures of N-acetylglucosamine-phosphate mutase, a member of the alpha-D-phosphohexomutase superfamily, and its substrate and product complexes. J Biol Chem.

[51] Jacob TR (2012). *rpb2* is a reliable reference gene for quantitative gene expression analysis in the dermatophyte *Trichophyton rubrum*. Med Mycol.

[52] CLSI. Reference method for broth dilution antifungal susceptibility testing of filamentous fungi. *Clinical Laboratory Standards Institute (CLSI)* approved standard-2nd Edition (2008).

[53] Thorvaldsdottir H, Robinson JT, Mesirov JP (2012). Integrative Genomics Viewer (IGV): high-performance genomics data visualization and exploration. Brief Bioinform.

[54] Robinson JT (2011). Integrative genomics viewer. Nat Biotechnol.

[55] Anders S, Huber W (2010). Differential expression analysis for sequence count data. Genome Biol.

[56] Vencio RZ, Koide T, Gomes SL, Pereira CA (2006). BayGO: Bayesian analysis of ontology term enrichment in microarray data. BMC Bioinformatics.

[57] Anders S, Pyl PT, Huber W (2015). HTSeq–a Python framework to work with high-throughput sequencing data. Bioinformatics.

[58] Anders S, Reyes A, Huber W (2012). Detecting differential usage of exons from RNA-seq data. Genome Res.

[59] Edgar R, Domrachev M, Lash AE (2002). Gene Expression Omnibus: NCBI gene expression and hybridization array data repository. Nucleic Acids Res.

[60] Schefe JH, Lehmann KE, Buschmann IR, Unger T, Funke-Kaiser H (2006). Quantitative real-time RT-PCR data analysis: current concepts and the novel “gene expression’s CT difference” formula. J Mol Med (Berl).

[61] Cove DJ (1966). The induction and repression of nitrate reductase in the fungus *Aspergillus nidulans*. Biochim Biophys Acta.

[62] Arthington-Skaggs BA, Jradi H, Desai T, Morrison CJ (1999). Quantitation of ergosterol content: novel method for determination of fluconazole susceptibility of *Candida albicans*. J Clin Microbiol.

[63] Ferreira-Nozawa MS (2006). The pH signaling transcription factor PacC mediates the growth of *Trichophyton rubrum* on human nail *in vitro*. Med Mycol.

[64] Martins MP, Franceschini AC, Jacob TR, Rossi A, Martinez-Rossi NM (2016). Compensatory expression of multidrug-resistance genes encoding ABC transporters in dermatophytes. J Med Microbiol.

[65] Schneider CA, Rasband WS, Eliceiri KW (2012). NIH Image to ImageJ: 25 years of image analysis. Nat Methods.

