# Supplementary material for: Transcriptome-wide survey of gene expression changes and alternative splicing in Trichophyton rubrum in response to undecanoic acid
Source: Sci Rep. 2018 Feb 6;8:2520. doi: 10.1038/s41598-018-20738-x (PMC5802734; doi:10.1038/s41598-018-20738-x)
Supplement: Supplementary file 1 — Supplementary Figures and Tables [file 41598_2018_20738_MOESM1_ESM.pdf]

Transcriptome-wide survey of gene expression changes and alternative splicing in *Trichophyton rubrum* in response to undecanoic acid

Niege S. Mendes[1], Tamires A. Bitencourt[1], Pablo R. Sanches[1], Rafael Silva-Rocha[2], Nilce M. Martinez-Rossi[1]* & Antonio Rossi[1]

[1]Department of Genetics, Ribeirão Preto Medical School, University of São Paulo, 14049-900 Ribeirão Preto, SP, Brazil

[2]Department of Molecular and Cellular Biology, Ribeirão Preto Medical School, University of São Paulo, 14049-900 Ribeirão Preto, SP, Brazil

* Correspondence and requests for materials should be addressed to N.M.M.-R. (email: nmmrossi@usp.br)

Tel: +55 16 33153150; Fax: +55 16 33150222;

Niege S. Mendes, Tamires A. Bitencourt, and Pablo R. Sanches contributed equally to this work.

Supplementary Figures

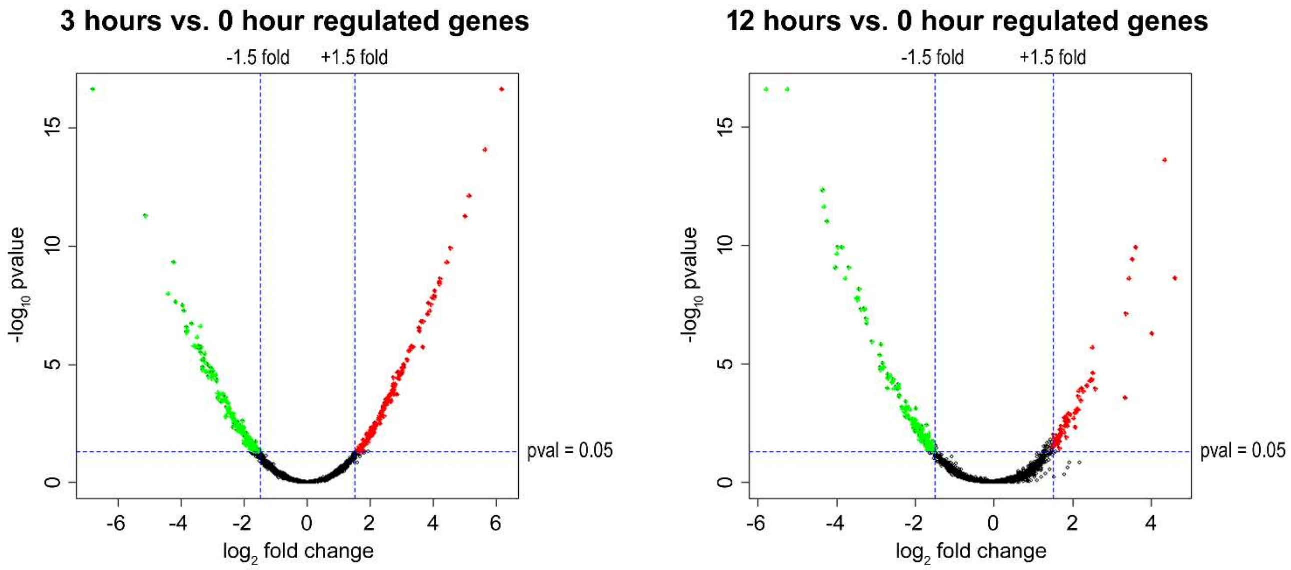

Supplementary Figure S1. Volcano plot of the different time points analysed. The $\log_2$ fold changes for modulated genes are plotted against the $-\log_{10}$ *P*-values under each condition.

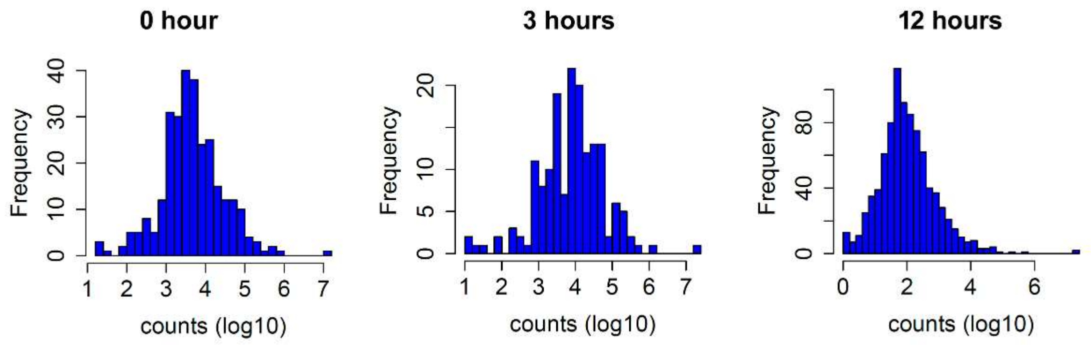

Supplementary Figure S2. Intron retention at each time point analysed. The distribution frequency is plotted against $-\log_{10}$ *P*-values.

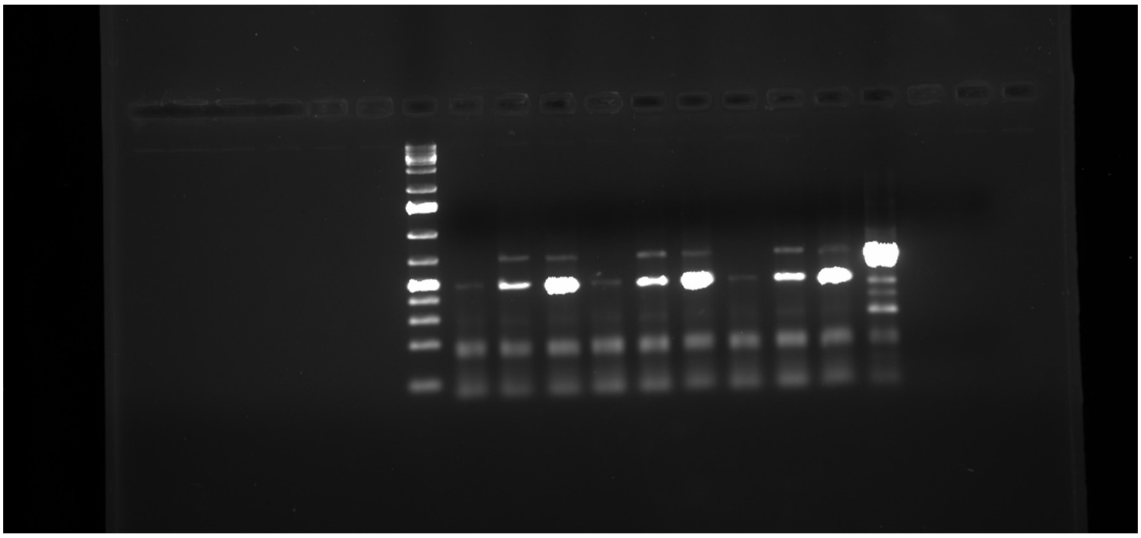

Supplementary Figure S3. RT-PCR showing the intron 2 retention in *impdh* gene in *T. rubrum*. The PCR amplicons were generated after electrophoresis in a 2% agarose gel containing ethidium bromide. The samples presented in this gel, starting from left side border: Molecular weight ladder (1kb plus, Thermo Scientific), 0h (control), 3h, and 12h time points of UDA exposure, assessed in first biological replicate, as well as for second, and third biological replicates, respectively. The last well is related to genomic DNA amplicon (PCR control). The expected size of each amplicon was 481 bp for the elimination of intron 2 and 684 bp for the retention of intron 2 (Full-length version agarose gel related to Fig. 7 agarose gel).

Supplementary Tables

Supplementary Table S1. General features of RNA-seq reads mapped to the *T. rubrum* reference genome.

Sample	Raw reads	High-quality reads	Mapped reads Bowtie2	Total mapped reads (%)
0 hour I (SR)	40,489,531	40,149,007	27,473,437	68.43
0 hour II (PE)	28,987,947	27,213,240	23,039,089	84.66
0 hour III (PE)	28,893,017	27,136,542	22,525,587	83.01
3 hours I (SR)	60,724,079	60,235,207	30,221,364	50.17
3 hours II (PE)	67,859,806	63,440,961	37,801,840	59.59
3 hours III (PE)	28,182,638	26,438,636	20,611,097	77.96
12 hours I (SR)	45,617,478	44,174,487	25,165,161	56.97
12 hours II (PE)	30,746,489	28,524,813	16,251,516	56.98
12 hours III (PE)	12,463,078	10,635,512	5,600,236	52.66

SR=Single-read; PE=Paired-end

Supplementary Table S2. Genes of *T. rubrum* modulated in response to UDA exposure at each time point.

3 hours vs. 0 hour

ID	3-0 hours	12-0 hours	Gene Product Name
TERG_01405	6.16		glutathione S-transferase (*T. equinum*)
TERG_04960	6.15		glutathione S-transferase Ure2-like, putative (*A. benhamiae*)
TERG_03078	5.63		cytochrome P450 oxidoreductase OrdA-like, putative (*T. verrucosum*)
TERG_08077	5.12		hypothetical protein
TERG_07830	4.99		hypothetical protein
TERG_00254	4.54		peroxisomal dehydratase (*T. equinum*)
TERG_01937	4.42		short chain dehydrogenase (*T. tonsurans*)
TERG_05621	4.02		short chain dehydrogenase/reductase family oxidoreductase, putative (*A. benhamiae*)
TERG_05484	4.02		acyl-CoA dehydrogenase (*T. tonsurans*)
TERG_04232	3.95		2-nitropropane dioxygenase (*T. equinum*)
TERG_00162	3.88		MFS multidrug transporter, putative (*A. benhamiae*)
TERG_11928	3.83		hypothetical protein
TERG_02909	3.81		acyl-CoA oxidase, putative (*T. verrucosum*)
TERG_01512	3.68		mitochondrial tricarboxylate transporter (Ctp), putative (*T. verrucosum*)
TERG_08182	3.65		hypothetical protein
TERG_03008	3.60		zinc-containing alcohol dehydrogenase, putative (*A. benhamiae*)
TERG_03483	3.54		carnitine acetyl transferase (*T. tonsurans*)
TERG_01233	3.34		3,2-trans-enoyl-CoA isomerase (*T. equinum*)
TERG_02747	3.34		cytochrome P450 alkane hydroxylase (*A. benhamiae*)
TERG_01742	3.33		hypothetical protein
TERG_04500	3.31		peroxisomal membrane protein Pmp47 (*T. tonsurans*)
TERG_02423	3.24		hypothetical protein
TERG_04714	3.23		mitochondrial phosphate carrier protein (*T. tonsurans*)
TERG_02041	3.21		glutathione S-transferase (*T. equinum*)
TERG_12351	3.15		hypothetical protein
TERG_02918	3.05		alkaline phytoceramidase (*T. equinum*)
TERG_03832	3.04		enoyl-CoA hydratase/isomerase (*T. equinum*)
TERG_07659	3.01		peroxisomal 3-ketoacyl-coA thiolase (Kat1), putative (*A. benhamiae*)
TERG_03390	3.00		glutation S-transferase (*T. equinum*)
TERG_07919	2.97		thioesterase family protein (*T. verrucosum*)
TERG_01784	2.91		oxidoreductase, short-chain dehydrogenase/reductase family, putative (*T. verrucosum*)
TERG_00216	2.88		endochitinase (*T. equinum*)
TERG_11539	2.86		3-oxoacyl-(acyl-carrier-protein) reductase (*T. tonsurans*)
TERG_07544	2.85		lipase (*T. tonsurans*)
TERG_02845	2.83		cercosporin toxin biosynthesis protein (*T. equinum*)
TERG_04389	2.78		carnitinyl-CoA dehydratase (*T. tonsurans*)
TERG_11538	2.77		3-oxoacyl-(acyl-carrier-protein) reductase (*T. tonsurans*)

TERG_02488	2.75	hypothetical protein
TERG_07691	2.73	sterol carrier protein (*T. tonsurans*)
TERG_08050	2.73	acetamidase (*T. equinum*)
TERG_01936	2.73	COP9 signalosome complex subunit 1 (*T. equinum*)
TERG_01077	2.72	metallo-beta-lactamase domain-containing protein (*T. equinum*)
TERG_06106	2.72	sulfate permease 2 (*T. tonsurans*)
TERG_00009	2.71	peroxisomal biogenesis factor (*T. tonsurans*)
TERG_11946	2.71	hypothetical protein
TERG_04620	2.68	peroxin 20 (*T. equinum*)
TERG_05575	2.65	MFS multidrug transporter (*T. tonsurans*)
TERG_11639	2.64	isocitrate lyase
TERG_04310	2.63	alcohol dehydrogenase (*T. tonsurans*)
TERG_00013	2.63	ThiJ/PfpI family protein (*A. benhamiae*)
TERG_07060	2.62	acyl-CoA thioesterase II
TERG_04010	2.60	sporulation protein SPS19 (*T. equinum*)
TERG_01833	2.59	peroxisomal membrane protein (*T. tonsurans*)
TERG_00950	2.56	hypothetical protein
TERG_06686	2.54	hypothetical protein
TERG_11638	2.54	isocitrate lyase
TERG_11637	2.54	isocitrate lyase
TERG_01270	2.53	AMP dependent ligase (*T. tonsurans*)
TERG_04837	2.53	thioesterase (*T. equinum*)
TERG_00284	2.52	peroxin 14 (*T. tonsurans*)
TERG_03305	2.52	C-4 methylsterol oxidase (*T. tonsurans*)
TERG_08336	2.51	MFS multidrug transporter, putative (*A. benhamiae*)
TERG_03907	2.48	neutral amino acid permease (*T. tonsurans*)
TERG_08051	2.46	3-oxoacyl-(acyl-carrier-protein) reductase (*T. tonsurans*)
TERG_11702	2.46	hypothetical protein
TERG_04817	2.44	hypothetical protein
TERG_12131	2.43	alcohol dehydrogenase (*T. tonsurans*)
TERG_01281	2.42	malate synthase, glyoxysomal
TERG_04224	2.41	ABC transporter
TERG_01641	2.35	FK506 suppressor Sfk1 (*T. equinum*)
TERG_03231	2.34	cytochrome P450 52A12 (*T. tonsurans*)
TERG_01986	2.34	Fe-S protein assembly co-chaperone HscB
TERG_00575	2.34	F-box domain protein (*T. verrucosum*)
TERG_03983	2.32	HPP family protein (*T. verrucosum*)
TERG_12497	2.31	hypothetical protein
TERG_08321	2.28	hypothetical protein
TERG_02533	2.26	aldose 1-epimerase (*T. equinum*)
TERG_03510	2.26	peroxisomal membrane anchor protein, putative (*A. benhamiae*)
TERG_03331	2.20	F-box protein (*T. equinum*)
TERG_06064	2.18	quinone oxidoreductase (*T. equinum*)

TERG_04092	2.16	peroxisomal matrix protein importer Pex3 (*T. tonsurans*)
TERG_12282	2.16	glycosyl hydrolase (*T. tonsurans*)
TERG_06622	2.16	hydrolase (*T. tonsurans*)
TERG_05551	2.16	hypothetical protein
TERG_05044	2.13	epoxide hydrolase, putative (*A. benhamiae*)
TERG_05562	2.13	2-hydroxychromene-2-carboxylate isomerase, putative (*A. benhamiae*)
TERG_06361	2.13	ATP-dependent protease La
TERG_00163	2.12	siderochrome-iron transporter, putative (*A. benhamiae*)
TERG_06177	2.12	mitochondrial carrier protein, putative (*T. verrucosum*)
TERG_02283	2.11	MFS transporter, putative (*T. verrucosum*)
TERG_05411	2.09	hypothetical protein
TERG_07331	2.08	integral membrane protein (*T. tonsurans*)
TERG_07224	2.06	hypothetical protein
TERG_07242	2.06	di-trans,poly-cis-decaprenylcistransferase
TERG_05199	2.06	MFS gliotoxin efflux transporter GliA (*T. verrucosum*)
TERG_01754	2.05	oxidoreductase, 2-nitropropane dioxygenase family, putative (*A. benhamiae*)
TERG_06494	2.05	hypothetical protein
TERG_08069	2.05	glutathione-disulfide reductase
TERG_00818	2.03	aldehyde dehydrogenase 3B1 (*T. equinum*)
TERG_02842	2.03	6-hydroxy-D-nicotine oxidase (*T. equinum*)
TERG_04422	2.02	NmrA family transcriptional regulator, putative (*T. verrucosum*)
TERG_05556	2.02	hypothetical protein
TERG_05023	2.01	calcium/proton exchanger
TERG_04598	2.01	NRPS-like enzyme, putative (*A. benhamiae*)
TERG_05244	2.00	hypothetical protein
TERG_04951	2.00	U-box domain-containing protein (*T. equinum*)
TERG_05622	1.98	uridine/cytidine kinase (*T. equinum*)
TERG_08519	1.98	mitochondrial enoyl reductase (*T. equinum*)
TERG_02403	1.97	glycolate oxidase, subunit GlcD
TERG_07957	1.96	hypothetical protein
TERG_01625	1.95	FMP32 (*M. gypseum*)
TERG_03780	1.95	pyruvate dehydrogenase dihydrolipoamide acetyltransferase component (*M. gypseum*)
TERG_12281	1.95	glycosyl hydrolase (*T. tonsurans*)
TERG_01759	1.94	peroxisome assembly protein 10 (*T. equinum*)
TERG_04766	1.94	calcium/proton exchanger
TERG_00701	1.93	cytochrome b5, putative (*T. verrucosum*)
TERG_05918	1.93	glutamyl-tRNA(Gln) amidotransferase subunit A (*T. equinum*)
TERG_12491	1.93	carboxylesterase, putative (*A. benhamiae*)
TERG_07533	1.91	hypothetical protein
TERG_11757	1.91	hypothetical protein
TERG_08066	1.91	STE/STE7 protein kinase
TERG_02508	1.91	ABC multidrug transporter, putative (*A. benhamiae*)

TERG_00616	1.90	potassium/sodium efflux P-type ATPase, fungal-type
TERG_00631	1.89	urate oxidase
TERG_08908	1.89	hypothetical protein
TERG_08116	1.88	hypothetical protein
TERG_03274	1.88	ammonium transporter MepA (*A. benhamiae*)
TERG_08909	1.84	hypothetical protein
TERG_06234	1.82	2,5-diketo-D-gluconic acid reductase A (*T. equinum*)
TERG_01621	1.81	DnaJ chaperone (Caj1), putative (*T. verrucosum*)
TERG_06016	1.81	glycosyl hydrolase (*T. tonsurans*)
TERG_06354	1.79	SVP1-like protein (*T. tonsurans*)
TERG_03177	1.78	RING finger domain protein, putative (*A. benhamiae*)
TERG_00439	1.77	hypothetical protein
TERG_08100	1.77	CDP-diacylglycerol-inositol 3-phosphatidyltransferase PIS (*T. equinum*)
TERG_11780	1.77	pre-mRNA splicing factor (*T. tonsurans*)
TERG_07872	1.76	homoserine O-acetyltransferase
TERG_01225	1.74	GTPase-activating protein GYP7 (*T. equinum*)
TERG_06594	1.73	DUF1183 domain-containing protein (*T. equinum*)
TERG_08104	1.73	potassium/sodium efflux P-type ATPase, fungal-type
TERG_12290	1.73	mitochondrial cytochrome b2 (*T. tonsurans*)
TERG_02039	1.72	2-hydroxyphytanoyl-CoA lyase (*T. tonsurans*)
TERG_02059	1.71	AbpA protein (*T. equinum*)
TERG_04332	1.71	phosphatidylinositol transporter (*T. tonsurans*)
TERG_05624	1.71	C6 transcription factor, putative (*T. verrucosum*)
TERG_03141	1.70	hypothetical protein
TERG_00577	1.69	BAR domain-containing protein (*T. tonsurans*)
TERG_08070	1.69	enoyl-CoA hydratase/isomerase (*T. tonsurans*)
TERG_02249	1.68	phenylacetyl-CoA ligase (*T. tonsurans*)
TERG_01676	1.67	6,7-dimethyl-8-ribityllumazine synthase
TERG_02038	1.67	peroxisomal targeting signal receptor (*T. equinum*)
TERG_05747	1.67	alcohol dehydrogenase (*T. tonsurans*)
TERG_07991	1.67	proline-rich protein (*T. tonsurans*)
TERG_01184	1.66	fatty acid-binding protein (*T. equinum*)
TERG_02087	1.66	hypothetical protein
TERG_04721	1.65	glutamate carboxypeptidase (*T. equinum*)
TERG_08952	1.65	long-chain-fatty-acid-CoA ligase (*T. equinum*)
TERG_01825	1.64	DNA repair protein Ntg1 (*T. tonsurans*)
TERG_02744	1.64	NAD-dependent methanol dehydrogenase (*T. equinum*)
TERG_04392	1.64	dephospho-CoA kinase
TERG_06732	1.63	peroxisomal membrane protein receptor Pex19, putative (*A. benhamiae*)
TERG_05359	1.62	peroxin-26 (*T. tonsurans*)
TERG_05373	1.62	peroxin 11C (*T. equinum*)
TERG_00919	1.61	Phospholipid:diacylglycerol acyltransferase (*T. equinum*)
TERG_05037	1.61	hypothetical protein

TERG_06240	1.60	peroxisomal biogenesis factor 2 (*T. tonsurans*)
TERG_08107	1.60	phosphoglycerate mutase (*T. tonsurans*)
TERG_04174	1.59	citrinin biosynthesis oxydoreductase CtnB (*T. equinum*)
TERG_06015	-1.52	nuclear import and export protein Msn5 (*T. tonsurans*)
TERG_00783	-1.55	guanine nucleotide-binding protein subunit beta-like protein
TERG_04478	-1.58	40S ribosomal protein S1
TERG_01550	-1.59	60S ribosomal protein L38 (*T. tonsurans*)
TERG_07641	-1.60	GPI-anchored cell wall protein Pst1, putative (*A. benhamiae*)
TERG_04323	-1.61	ATP-dependent bile acid permease (*T. equinum*)
TERG_08869	-1.62	hypothetical protein
TERG_02604	-1.64	40S ribosomal protein S20
TERG_04324	-1.64	extracellular metalloproteinase 4
TERG_04834	-1.64	rab GDP-dissociation inhibitor (*T. tonsurans*)
TERG_02242	-1.64	yeast phase specific protein (*M. gypseum*)
TERG_07065	-1.65	UMTA (*T. equinum*)
TERG_07865	-1.65	septin-1 (*T. equinum*)
TERG_01953	-1.67	60S acidic ribosomal protein P2
TERG_11551	-1.67	telomere and ribosome associated protein Stm1 (*T. tonsurans*)
TERG_12231	-1.68	DUF221 domain-containing protein (*T. equinum*)
TERG_00657	-1.69	hypothetical protein
TERG_06646	-1.70	arrestin (*T. equinum*)
TERG_05971	-1.72	ribonucleoside-diphosphate reductase small chain
TERG_01330	-1.72	GPI anchored protein, putative (*A. benhamiae*)
TERG_00511	-1.73	hypothetical protein
TERG_02630	-1.74	histone H2A
TERG_06481	-1.74	cell polarity protein (*T. tonsurans*)
TERG_12232	-1.74	DUF221 domain-containing protein (*T. equinum*)
TERG_05576	-1.75	cell wall glucanase (Scw11), putative (*A. benhamiae*)
TERG_07283	-1.75	GABA permease (*T. equinum*)
TERG_07269	-1.75	mannose-1-phosphate guanyltransferase
TERG_01165	-1.75	40S ribosomal protein S10b (*T. equinum*)
TERG_04658	-1.76	imidazole glycerol phosphate synthase hisHF
TERG_12294	-1.76	histidine acid phosphatase, putative (*T. verrucosum*)
TERG_02263	-1.76	hypothetical protein
TERG_06854	-1.76	glutamine-serine-proline rich protein, putative (*A. benhamiae*)
TERG_01397	-1.77	C2H2 finger domain-containing protein (*T. equinum*)
TERG_06584	-1.77	hypothetical protein
TERG_07770	-1.77	importin 13, putative (*T. verrucosum*)
TERG_05374	-1.78	hypothetical protein
TERG_08226	-1.79	hypothetical protein
TERG_01443	-1.80	ABC multidrug transporter (*T. tonsurans*)
TERG_04862	-1.80	C6 transcription factor RosA (*A. benhamiae*)
TERG_05644	-1.80	mucin family signaling protein Msb2, putative (*T. verrucosum*)

TERG_02197	-1.80	nitrilase, putative (*T. verrucosum*)
TERG_06118	-1.80	hypothetical protein
TERG_11550	-1.82	telomere and ribosome associated protein Stm1, putative (*T. verrucosum*)
TERG_00707	-1.82	GPI anchored serine-threonine rich protein (*T. verrucosum*)
TERG_02496	-1.84	histone H1 (*T. tonsurans*)
TERG_02795	-1.85	thiazole biosynthetic enzyme, mitochondrial
TERG_07543	-1.85	hypothetical protein
TERG_04514	-1.86	cell division control protein 12 (*T. tonsurans*)
TERG_00414	-1.86	3-beta hydroxysteroid dehydrogenase/isomerase family protein (*A. benhamiae*)
TERG_01012	-1.87	hypothetical protein
TERG_08234	-1.87	TCTP family protein (*T. verrucosum*)
TERG_07059	-1.91	hypothetical protein
TERG_02081	-1.94	RNA binding protein
TERG_03352	-1.94	tyrosine decarboxylase (*T. equinum*)
TERG_01444	-1.95	nonribosomal peptide synthase Pes1 (*A. benhamiae*)
TERG_03183	-1.95	hypothetical protein
TERG_05626	-1.96	chitinase (*T. equinum*)
TERG_03621	-1.96	hypothetical protein
TERG_00543	-1.98	A-agglutinin anchorage subunit (*M. canis*)
TERG_03382	-1.98	ThiJ/PfpI family protein (*A. benhamiae*)
TERG_08225	-2.01	hypothetical protein
TERG_01623	-2.02	MFS transporter (*T. equinum*)
TERG_00520	-2.04	hypothetical protein
TERG_05409	-2.04	FAD dependent oxidoreductase (*T. equinum*)
TERG_03702	-2.05	hypothetical protein
TERG_04558	-2.05	nucleoside diphosphate kinase
TERG_02735	-2.06	fatty acid oxygenase PpoC, putative (*A. benhamiae*)
TERG_01744	-2.07	hypothetical protein
TERG_01786	-2.11	DENN domain-containing protein (*T. tonsurans*)
TERG_08987	-2.11	hypothetical protein
TERG_02367	-2.12	hypothetical protein
TERG_05651	-2.15	hypothetical protein
TERG_05735	-2.20	dipeptidyl peptidase 4
TERG_01638	-2.22	hypothetical protein
TERG_00830	-2.22	cytochrome P450 monooxygenase, putative (*A. benhamiae*)
TERG_03758	-2.26	hypothetical protein
TERG_08622	-2.29	transferase (*M. canis*)
TERG_08129	-2.35	general amino-acid permease GAP1 (*T. equinum*)
TERG_08620	-2.35	siderophore iron transporter (*T. equinum*)
TERG_06047	-2.36	hypothetical protein
TERG_08557	-2.37	carboxypeptidase S1, putative (*A. benhamiae*)
TERG_05524	-2.44	3-deoxy-7-phosphoheptulonate synthase

TERG_08240	-2.48	methyltransferase, putative (*T. verrucosum*)
TERG_02368	-2.49	extracellular developmental signal biosynthesis protein FluG (*A. benhamiae*)
TERG_02189	-2.50	tubulin alpha-2 chain
TERG_06230	-2.52	hypothetical protein
TERG_04308	-2.54	MFS sugar transporter (*T. tonsurans*)
TERG_00481	-2.56	beta-glucosidase, putative (*T. verrucosum*)
TERG_01685	-2.57	glutathione S-transferase, putative (*A. benhamiae*)
TERG_07904	-2.59	tubulin beta chain
TERG_04227	-2.60	ABC transporter (*T. tonsurans*)
TERG_04964	-2.60	RNA-binding protein (*A. benhamiae*)
TERG_02118	-2.60	GPI anchored protein, putative (*A. benhamiae*)
TERG_02610	-2.61	WD repeat protein (*T. verrucosum*)
TERG_11951	-2.67	prp 6 CRoW domain-containing protein (*M. canis*)
TERG_03985	-2.68	hypothetical protein
TERG_12722	-2.71	transferase (*M. canis*)
TERG_01401	-2.80	high affinity copper transporter (*T. tonsurans*)
TERG_03624	-2.88	SUN domain protein (Uth1), putative (*T. verrucosum*)
TERG_07456	-2.90	cell wall protein PhiA (*T. equinum*)
TERG_05816	-2.91	hypothetical protein
TERG_07469	-2.94	SUN domain-containing protein (*T. equinum*)
TERG_12721	-2.99	transferase (*M. canis*)
TERG_06934	-3.03	hypothetical protein
TERG_01869	-3.06	TOS1 (*T. equinum*)
TERG_04855	-3.15	hypothetical protein
TERG_00598	-3.22	hypothetical protein
TERG_01731	-3.39	hypothetical protein
TERG_05424	-3.47	hypothetical protein
TERG_02057	-3.51	hypothetical protein
TERG_08619	-3.56	siderophore iron transporter mirB (*T. equinum*)
TERG_06144	-3.68	cell wall serine-threonine-rich galactomannoprotein Mp1 (*A. benhamiae*)
TERG_02023	-3.96	extracellular matrix protein, putative (*T. verrucosum*)
TERG_02278	-4.24	hypothetical protein

Gene expression values are expressed in log_2 fold change between each time point.

12 hours vs. 0 hour

ID	3-0 hours	12-0 hours	Gene Product Name
TERG_05426		4.59	choline-sulfatase
TERG_00092		4.01	ubiquinone biosynthesis methyltransferase coq5 (*T. equinum*)
TERG_00508		3.35	hypothetical protein
TERG_05288		3.33	aprataxin-like protein (*T. equinum*)
TERG_11963		2.50	hypothetical protein

TERG_08264	2.50	L-xylulose reductase
TERG_06727	2.48	homoserine dehydrogenase
TERG_08969	2.46	cytosolic Cu/Zn superoxide dismutase, putative (*A. benhamiae*)
TERG_00865	2.43	hypothetical protein
TERG_01252	2.37	catalase A
TERG_07071	2.35	2-isopropylmalate synthase
TERG_07802	2.27	siderophore biosynthesis acetylase AceI, putative (*T. verrucosum*)
TERG_08436	2.22	hypothetical protein
TERG_03826	2.14	hypothetical protein
TERG_08261	2.12	glutamate decarboxylase
TERG_01741	2.06	hypothetical protein
TERG_01347	2.05	TIGR01456 family HAD hydrolase
TERG_04504	1.93	extracellular protein (*T. equinum*)
TERG_08139	1.89	NAD dependent epimerase/dehydratase (*M. gypseum*)
TERG_05737	1.88	hypothetical protein
TERG_07631	1.88	hypothetical protein
TERG_06735	1.86	oxysterol binding protein (*T. tonsurans*)
TERG_04315	1.84	conserved glutamic acid rich protein (*T. verrucosum*)
TERG_03212	1.83	hypothetical protein
TERG_05466	1.80	MFS transporter, putative (*T. verrucosum*)
TERG_01979	1.79	pirin domain protein, putative (*A. benhamiae*)
TERG_07291	1.78	hypothetical protein
TERG_08771	1.78	extracellular serine-threonine rich protein (*T. tonsurans*)
TERG_00060	1.72	GPI anchored cell wall protein, putative (*A. benhamiae*)
TERG_03193	1.70	hypothetical protein
TERG_02641	1.70	cystathionine gamma-synthase (*T. equinum*)
TERG_03896	1.68	mannose-6-phosphate isomerase
TERG_05003	1.66	NmrA family protein (*T. equinum*)
TERG_00942	1.64	hypothetical protein
TERG_05841	1.63	AmmeMemoRadiSam system protein B
TERG_06927	1.61	hypothetical protein
TERG_12156	1.61	hypothetical protein
TERG_08353	1.59	cytochrome P450 55A3 (*T. tonsurans*)
TERG_00573	1.52	hypothetical protein
TERG_03834	1.52	ketol-acid reductoisomerase, mitochondrial
TERG_03875	1.51	glycogen phosphorylase (*T. equinum*)
TERG_07427	-1.51	homeobox and C2H2 transcription factor, putative (*A. benhamiae*)
TERG_01904	-1.53	hypothetical protein
TERG_04184	-1.54	CorA family metal ion transporter, putative (*A. benhamiae*)
TERG_05450	-1.55	acetyltransferase (*T. tonsurans*)
TERG_02787	-1.56	polyketide synthase (*T. tonsurans*)
TERG_00956	-1.60	extracellular thaumatin domain-containing protein (*T. equinum*)
TERG_03383	-1.60	hypothetical protein

TERG_02303	-1.60	ankyrin repeat protein (*A. benhamiae*)
TERG_00671	-1.61	methyltransferase (*T. equinum*)
TERG_03935	-1.62	hypothetical protein
TERG_12580	-1.62	dihydrodipicolinate synthetase family protein (*T. verrucosum*)
TERG_07139	-1.63	hypothetical protein
TERG_01430	-1.64	hypothetical protein
TERG_12085	-1.66	hypothetical protein
TERG_03157	-1.67	NDT80 / PhoG like DNA-binding family protein (*A. benhamiae*)
TERG_12093	-1.67	poly(ADP)-ribose polymerase PARP, putative (*T. verrucosum*)
TERG_00553	-1.69	amino acid permease (*T. equinum*)
TERG_03777	-1.69	hypothetical protein
TERG_05604	-1.69	C6 finger domain-containing protein (*T. equinum*)
TERG_08195	-1.70	peptidase S41 family protein (*M. gypseum*)
TERG_02541	-1.71	FAD-dependent monooxygenase, putative (*T. verrucosum*)
TERG_02837	-1.73	3-beta hydroxysteroid dehydrogenase/isomerase family protein, putative (*T. verrucosum*)
TERG_03963	-1.73	mannosyl phosphorylinositol ceramide synthase SUR1 (*T. equinum*)
TERG_08029	-1.74	HMG box protein, putative (*T. verrucosum*)
TERG_00539	-1.75	F-box and WD40 domain-containing protein (*T. tonsurans*)
TERG_02902	-1.76	hypothetical protein
TERG_07282	-1.77	cytochrome P450 monooxygenase, putative (*T. verrucosum*)
TERG_02593	-1.81	C2H2 finger domain protein, putative (*T. verrucosum*)
TERG_07346	-1.81	tRNA ligase (*M. gypseum*)
TERG_01609	-1.82	Na/K ATPase alpha 1 subunit (*T. equinum*)
TERG_05680	-1.82	IBR domain containing protein (*T. equinum*)
TERG_08278	-1.83	serine/threonine protein kinase (*T. tonsurans*)
TERG_00257	-1.84	conserved predicted protein (*A. benhamiae*)
TERG_03095	-1.84	methyltransferase, putative (*A. benhamiae*)
TERG_04359	-1.84	hypothetical protein
TERG_08045	-1.84	DUF455 domain-containing protein (*T. equinum*)
TERG_03984	-1.86	major facilitator superfamily transporter MFS-1 (*M. canis*)
TERG_05987	-1.86	GTP-binding protein 1 (*T. equinum*)
TERG_08961	-1.87	hypothetical protein
TERG_05617	-1.89	hypothetical protein
TERG_02664	-1.90	hypothetical protein
TERG_03681	-1.90	leucine aminopeptidase (*T. equinum*)
TERG_07597	-1.92	SOK1 (*T. equinum*)
TERG_06552	-1.94	aspartic-type endopeptidase (OpsB), putative (*T. verrucosum*)
TERG_05616	-1.95	hypothetical protein
TERG_01728	-1.96	hypothetical protein
TERG_03929	-1.98	hypothetical protein
TERG_02350	-1.99	endochitinase (*T. equinum*)
TERG_00199	-2.00	serine/threonine protein kinase

TERG_07037	-2.01	hypothetical protein
TERG_01511	-2.03	hypothetical protein
TERG_05469	-2.03	hypothetical protein
TERG_08613	-2.04	ABC multidrug transporter, putative (*A. benhamiae*)
TERG_00638	-2.06	cellobiose dehydrogenase, putative (*A. benhamiae*)
TERG_02958	-2.06	sulfite reductase flavoprotein component (*T. equinum*)
TERG_03707	-2.07	geranylgeranyl diphosphate synthase, putative (*A. benhamiae*)
TERG_05492	-2.07	hypothetical protein
TERG_03384	-2.10	aspartate aminotransferase, putative (*A. benhamiae*)
TERG_06679	-2.14	MFS transporter, putative (*A. benhamiae*)
TERG_05456	-2.16	hypothetical protein
TERG_08298	-2.18	RTA1 domain-containing protein (*T. equinum*)
TERG_00709	-2.20	MOSC domain-containing protein (*T. equinum*)
TERG_06347	-2.25	hypothetical protein
TERG_00734	-2.26	dipeptidase (*T. tonsurans*)
TERG_08022	-2.27	bZIP transcription factor CpcA (*T. verrucosum*)
TERG_12606	-2.33	protease DPPV, putative (*A. benhamiae*)
TERG_02400	-2.35	amino acid transporter, putative (*A. benhamiae*)
TERG_02517	-2.41	N-acetyltransferase, GNAT family, putative (*A. benhamiae*)
TERG_02183	-2.43	C2H2 finger domain-containing protein (*T. equinum*)
TERG_07008	-2.46	diaminopropionate ammonia-lyase (*T. equinum*)
TERG_07673	-2.56	hypothetical protein
TERG_00066	-2.59	4-hydroxyphenylpyruvate dioxygenase
TERG_05451	-2.59	integral membrane protein (*T. equinum*)
TERG_00068	-2.61	homogentisate 1,2-dioxygenase
TERG_12438	-2.63	gamma-glutamyltranspeptidase (*T. tonsurans*)
TERG_06701	-2.69	gamma-glutamyltransferase
TERG_01782	-2.70	hypothetical protein
TERG_02534	-2.70	hypothetical protein
TERG_05650	-2.71	blasticidin-resistance protein (*T. tonsurans*)
TERG_11997	-2.88	potassium uptake transporter (*T. equinum*)
TERG_00736	-3.25	alpha/beta hydrolase, putative (*A. benhamiae*)
TERG_12029	-3.29	hypothetical protein
TERG_08980	-3.47	hypothetical protein
TERG_07516	-5.80	hypothetical protein

3 hours vs. 0 hour and 12 hours vs. 0 hour

ID	3-0 hours	12-0 hours	Gene Product Name
TERG_00823	4.22	2.08	rhamnolipids biosynthesis 3-oxoacyl-[acyl-carrier-protein] reductase (*T. equinum*)
TERG_05363	4.19	1.67	hypothetical protein

TERG_12530	4.19	1.50	3-ketoacyl-CoA thiolase peroxisomal A (*T. tonsurans*)
TERG_06509	4.04	1.63	glutamate synthase (*T. tonsurans*)
TERG_07143	3.92	3.51	potassium/sodium efflux P-type ATPase, fungal-type
TERG_04038	3.67	2.08	acyl-CoA dehydrogenase (*T. tonsurans*)
TERG_03154	3.55	1.53	2-nitropropane dioxygenase (*M. gypseum*)
TERG_07721	3.34	2.20	isopenicillin N-CoA epimerase (*T. equinum*)
TERG_03881	3.29	1.66	epoxide hydrolase (*T. tonsurans*)
TERG_08102	3.28	4.34	3-isopropylmalate dehydrogenase A
TERG_01463	3.17	2.15	cytochrome c peroxidase (*T. tonsurans*)
TERG_01122	2.99	2.14	heat shock protein Awh11, putative (*A. benhamiae*)
TERG_06155	2.90	1.54	phosphotransferase (*T. tonsurans*)
TERG_04765	2.72	3.42	MFS transporter, putative (*A. benhamiae*)
TERG_04753	2.49	3.60	3-isopropylmalate dehydratase, large subunit
TERG_01349	2.41	1.60	glutathione peroxidase (*T. tonsurans*)
TERG_03464	2.35	1.81	stress response protein (*T. equinum*)
TERG_08170	2.27	1.89	fatty acid elongase gig30 (*T. equinum*)
TERG_08825	2.00	-1.62	bZIP transcription factor JlbA/IDI-4 (*T. verrucosum*)
TERG_05769	1.98	2.02	NAD(P)H-dependent D-xylose reductase (*T. equinum*)
TERG_08245	1.95	1.70	branched-chain amino acid aminotransferase (*T. tonsurans*)
TERG_01190	1.94	1.61	RNA binding protein (*T. equinum*)
TERG_03278	1.93	2.58	hypothetical protein
TERG_02767	1.91	1.51	pyroglutamyl peptidase type I (*T. equinum*)
TERG_08347	1.76	-1.55	cytochrome P450 monooxygenase, putative (*A. benhamiae*)
TERG_00523	1.63	-2.57	hypothetical protein
TERG_05615	-1.67	-2.24	hsp70-like protein (*T. equinum*)
TERG_11968	-1.71	-1.98	hypothetical protein
TERG_11814	-1.75	-2.43	FAD dependent oxidoreductase, putative (*A. benhamiae*)
TERG_12682	-1.77	-1.87	hypothetical protein
TERG_07410	-1.83	-1.63	hypothetical protein
TERG_07796	-1.88	-2.90	oxidoreductase, short-chain dehydrogenase/reductase family (*A. benhamiae*)
TERG_00069	-1.90	-1.59	fumarylacetoacetase
TERG_04917	-1.91	-1.51	thiamine pyrophosphate enzyme, putative (*A. benhamiae*)
TERG_05274	-1.95	-1.92	hypothetical protein
TERG_00281	-1.95	-2.05	AGC/RSK protein kinase
TERG_07408	-1.98	-1.63	acetyltransferase, GNAT family (*A. benhamiae*)
TERG_06022	-2.01	-1.92	hypothetical protein
TERG_05055	-2.02	-1.64	MFS multidrug transporter (*T. tonsurans*)
TERG_02406	-2.02	-2.55	alpha-box mating type protein (*T. tonsurans*)
TERG_08130	-2.03	-2.28	ABC ATPase (*T. equinum*)
TERG_04240	-2.09	1.85	cupin 2 domain-containing protein (*T. equinum*)
TERG_04543	-2.11	-1.84	aminotransferase GliI (*T. equinum*)
TERG_11998	-2.12	-2.50	potassium uptake transporter (*T. equinum*)
TERG_07892	-2.13	-1.83	SCP-like extracellular protein, putative (*A. benhamiae*)

TERG_07409	-2.17	-2.04	amino acid permease (*T. equinum*)
TERG_07795	-2.25	-1.72	WSC domain protein, putative (*A. benhamiae*)
TERG_05746	-2.25	-1.75	hypothetical protein
TERG_12298	-2.31	-1.57	hypothetical protein
TERG_05625	-2.35	-2.57	class V chitinase, putative (*T. verrucosum*)
TERG_06055	-2.36	-1.66	NmrA-like family protein (*A. benhamiae*)
TERG_06358	-2.36	-1.68	dicer (*T. tonsurans*)
TERG_02214	-2.37	-2.02	carboxypeptidase 2
TERG_06625	-2.42	-2.06	serine protease, putative (*A. benhamiae*)
TERG_03851	-2.43	-2.07	serine/threonine protein kinase
TERG_01116	-2.43	-2.24	hypothetical protein
TERG_02746	-2.51	-4.25	hypothetical protein
TERG_07665	-2.52	-2.26	hypothetical protein
TERG_08614	-2.58	-2.02	hypothetical protein
TERG_08046	-2.60	-2.42	beta-lactamase (*M. gypseum*)
TERG_00791	-2.65	-2.22	developmental regulatory protein WetA (*T. verrucosum*)
TERG_12578	-2.72	-2.83	PEP phosphonomutase (*T. equinum*)
TERG_00967	-2.74	-1.86	hypothetical protein
TERG_05799	-2.81	-2.89	chlorophyll synthesis pathway protein BchC
TERG_05545	-2.82	-1.75	GNAT family acetyltransferase, putative (*A. benhamiae*)
TERG_03719	-2.84	-2.87	MFS sugar transporter (*T. tonsurans*)
TERG_03955	-2.85	-2.42	RNA 3'-terminal phosphate cyclase, putative (*T. verrucosum*)
TERG_08363	-3.01	-1.61	tyrosinase (*T. verrucosum*)
TERG_01599	-3.03	-3.30	hypothetical protein
TERG_05854	-3.12	-3.47	beta-lactamase (*T. tonsurans*)
TERG_07184	-3.13	-3.25	HMG box transcriptional regulator, putative (*A. benhamiae*)
TERG_03706	-3.21	-1.69	trichodiene oxygenase (*T. equinum*)
TERG_06883	-3.25	-3.69	NAD dependent epimerase/dehydratase family protein (*T. verrucosum*)
TERG_01981	-3.26	-4.36	hypothetical protein
TERG_11747	-3.31	-3.10	hypothetical protein
TERG_05627	-3.33	-3.50	LysM domain-containing protein (*M. canis*)
TERG_06291	-3.33	-4.33	malate dehydrogenase (*T. verrucosum*)
TERG_07734	-3.34	-3.38	O-methyltransferase, putative (*T. verrucosum*)
TERG_00911	-3.37	-3.96	hypothetical protein
TERG_03343	-3.38	-1.83	fatty acid desaturase (*T. equinum*)
TERG_00806	-3.43	-3.78	hypothetical protein
TERG_03981	-3.54	-3.24	hypothetical protein
TERG_12627	-3.60	-4.00	C-5 sterol desaturase (*T. equinum*)
TERG_00487	-3.82	-2.18	hypothetical protein
TERG_07769	-3.83	-5.25	hypothetical protein
TERG_12626	-3.84	-4.04	C-5 sterol desaturase (*T. equinum*)
TERG_01338	-3.92	-2.79	hydantoinase (*T. equinum*)
TERG_08004	-4.18	-3.86	hypothetical protein

TERG_04547	-4.42	-3.43	aquaporin (*T. tonsurans*)
TERG_04234	-5.14	-1.93	hydrophobin, putative (*T. verrucosum*)
TERG_01480	-6.81	-1.93	MFS multidrug transporter, putative (*A. benhamiae*)

Supplementary Table S3. Differential exon usage obtained from RNA-seq data analysis using DEXSeq.

3 hours vs. 0 hour

ID	Exon	padj	3-0 hours	Gene Product Name
TERG_00069	E001	5.62E-04	0.28	fumarylacetoacetase
TERG_00069	E002	2.82E-04	-0.06	fumarylacetoacetase
TERG_00074	E001	1.43E-02	-0.07	biotin synthase
TERG_00074	E002	1.37E-02	0.05	biotin synthase
TERG_00098	E001	2.64E-02	0.21	high affinity methionine permease (*A. benhamiae*)
TERG_00102	E001	3.48E-02	-0.07	fumarate hydratase, class II
TERG_00138	E002	1.51E-02	6.49	tetraspanin (*T. equinum*)
TERG_00216	E004	2.49E-03	0.59	endochitinase (*T. equinum*)
TERG_00216	E007	8.72E-05	-0.13	endochitinase (*T. equinum*)
TERG_00242	E005	1.75E-04	0.52	60S ribosomal protein L27-A (*T. tonsurans*)
TERG_00284	E002	1.01E-06	1.37	peroxin 14 (*T. tonsurans*)
TERG_00332	E001	1.12E-10	0.10	nuclear transport factor 2
TERG_00332	E002	1.82E-02	0.05	nuclear transport factor 2
TERG_00332	E004	1.57E-02	-0.05	nuclear transport factor 2
TERG_00332	E005	4.10E-72	-0.75	nuclear transport factor 2
TERG_00332	E006	1.54E-17	-0.41	nuclear transport factor 2
TERG_00346	E002	4.67E-02	-6.35	hypothetical protein
TERG_00384	E005	4.94E-02	0.08	Ser/Thr protein phosphatase family protein (*A. benhamiae*)
TERG_00438	E003	3.44E-05	-0.30	transcription factor SipA3 (*T. tonsurans*)
TERG_00438	E005	1.93E-02	-0.47	transcription factor SipA3 (*T. tonsurans*)
TERG_00438	E006	3.45E-02	-0.20	transcription factor SipA3 (*T. tonsurans*)
TERG_00438	E008	4.39E-06	0.48	transcription factor SipA3 (*T. tonsurans*)
TERG_00461	E013	3.20E-02	-0.08	phenylalanine-tRNA ligase, beta subunit
TERG_00481	E008	4.41E-02	-0.35	beta-glucosidase, putative (*T. verrucosum*)
TERG_00580	E003	2.17E-02	0.67	7-dehydrocholesterol reductase (*T. equinum*)
TERG_00597	E001	9.32E-05	0.33	methyltransferase (*T. equinum*)
TERG_00597	E003	3.18E-02	-0.37	methyltransferase (*T. equinum*)
TERG_00616	E007	6.28E-03	-0.67	potassium/sodium efflux P-type ATPase, fungal-type
TERG_00624	E007	6.06E-03	0.09	actin polymerization protein Bzz1 (*T. tonsurans*)
TERG_00659	E002	1.46E-02	-0.61	3-deoxy-7-phosphoheptulonate synthase
TERG_00686	E001	8.71E-03	0.10	xaa-Pro dipeptidase (*T. equinum*)
TERG_00686	E003	2.11E-02	-0.24	xaa-Pro dipeptidase (*T. equinum*)
TERG_00748	E003	2.34E-02	-0.22	argininosuccinate synthase
TERG_00798	E001	3.94E-03	0.29	zinc knuckle domain protein (*T. verrucosum*)
TERG_00820	E001	9.95E-06	0.18	MFS multidrug transporter, putative (*A. benhamiae*)
TERG_00820	E002	6.35E-03	0.35	MFS multidrug transporter, putative (*A. benhamiae*)
TERG_00829	E002	1.75E-02	-0.09	hypothetical protein
TERG_00829	E003	4.25E-02	0.39	hypothetical protein
TERG_00830	E002	5.10E-03	-0.18	cytochrome P450 monooxygenase, putative (*A. benhamiae*)
TERG_00831	E005	1.72E-02	0.06	gamma-glutamyltransferase
TERG_00835	E002	2.37E-08	1.67	hypothetical protein
TERG_00865	E002	2.59E-02	-0.37	hypothetical protein

TERG_00865	E007	1.29E-12	-0.28	hypothetical protein
TERG_00865	E008	9.51E-06	-0.68	hypothetical protein
TERG_00877	E001	5.67E-04	0.29	CBS domain-containing protein (*T. equinum*)
TERG_00877	E003	6.24E-03	-0.22	CBS domain-containing protein (*T. equinum*)
TERG_00877	E004	1.89E-04	-0.28	CBS domain-containing protein (*T. equinum*)
TERG_00908	E001	2.45E-02	-0.31	extensin (*M. canis*)
TERG_00978	E002	5.72E-03	-0.30	RNA processing protein Grc3, putative (*T. verrucosum*)
TERG_00978	E008	2.73E-03	0.07	RNA processing protein Grc3, putative (*T. verrucosum*)
TERG_01012	E001	3.40E-08	-0.14	hypothetical protein
TERG_01012	E002	4.96E-02	0.48	hypothetical protein
TERG_01012	E003	1.09E-03	0.87	hypothetical protein
TERG_01012	E004	7.94E-07	0.45	hypothetical protein
TERG_01046	E001	2.82E-12	-0.23	H/ACA ribonucleoprotein complex subunit 1
TERG_01077	E001	8.62E-03	0.40	metallo-beta-lactamase domain-containing protein (*T. equinum*)
TERG_01077	E002	9.08E-04	0.83	metallo-beta-lactamase domain-containing protein (*T. equinum*)
TERG_01118	E007	3.60E-04	0.51	AhpC/TSA family protein (*T. equinum*)
TERG_01151	E003	2.99E-02	0.05	26S proteasome non-ATPase regulatory subunit 13 (*T. equinum*)
TERG_01163	E002	6.31E-33	0.64	Hnt1 cyclin dependent kinase Kin28 interacting protein (*T. tonsurans*)
TERG_01163	E004	1.56E-03	-0.09	Hnt1 cyclin dependent kinase Kin28 interacting protein (*T. tonsurans*)
TERG_01163	E006	5.21E-05	-0.17	Hnt1 cyclin dependent kinase Kin28 interacting protein (*T. tonsurans*)
TERG_01198	E002	1.51E-03	-0.13	pre-mRNA-splicing factor rse1
TERG_01198	E003	1.23E-04	-0.20	pre-mRNA-splicing factor rse1
TERG_01198	E006	1.27E-38	0.21	pre-mRNA-splicing factor rse1
TERG_01239	E001	1.99E-11	0.20	Mis6 domain-containing protein (*T. equinum*)
TERG_01239	E002	1.79E-08	-0.85	Mis6 domain-containing protein (*T. equinum*)
TERG_01239	E003	6.46E-03	-6.18	Mis6 domain-containing protein (*T. equinum*)
TERG_01267	E002	4.58E-03	0.97	amidophosphoribosyltransferase (*T. equinum*)
TERG_01273	E001	1.23E-04	0.20	alpha-1,2-mannosidase, putative subfamily (*T. verrucosum*)
TERG_01273	E002	1.43E-04	-0.26	alpha-1,2-mannosidase, putative subfamily (*T. verrucosum*)
TERG_01279	E001	2.01E-06	-0.95	flavin-binding monooxygenase (*T. equinum*)
TERG_01279	E002	5.68E-11	-0.78	flavin-binding monooxygenase (*T. equinum*)
TERG_01279	E003	1.73E-06	-0.81	flavin-binding monooxygenase (*T. equinum*)
TERG_01279	E004	5.48E-06	-0.98	flavin-binding monooxygenase (*T. equinum*)
TERG_01279	E007	1.10E-21	0.36	flavin-binding monooxygenase (*T. equinum*)
TERG_01282	E001	1.01E-04	0.23	hypothetical protein
TERG_01282	E002	1.90E-04	-0.39	hypothetical protein
TERG_01336	E001	2.75E-02	0.49	MFS transporter (*T. equinum*)
TERG_01356	E001	5.87E-03	-0.12	small G-protein GPA2 (*T. tonsurans*)
TERG_01363	E001	1.26E-03	-0.18	SET domain-containing protein (*T. equinum*)
TERG_01363	E004	9.53E-03	0.60	SET domain-containing protein (*T. equinum*)
TERG_01401	E004	9.83E-05	0.63	high affinity copper transporter (*T. tonsurans*)
TERG_01401	E005	1.77E-03	-0.01	high affinity copper transporter (*T. tonsurans*)
TERG_01419	E001	2.37E-03	-0.02	protein pyrABCN

TERG_01419	E002	3.97E-03	0.18	protein pyrABCN
TERG_01480	E001	1.48E-83	-1.17	MFS multidrug transporter, putative (*A. benhamiae*)
TERG_01480	E002	1.28E-46	-0.97	MFS multidrug transporter, putative (*A. benhamiae*)
TERG_01480	E003	1.81E-151	0.55	MFS multidrug transporter, putative (*A. benhamiae*)
TERG_01481	E001	7.52E-84	-0.51	MFS multidrug transporter, putative (*A. benhamiae*)
TERG_01481	E002	5.84E-08	0.47	MFS multidrug transporter, putative (*A. benhamiae*)
TERG_01481	E003	3.84E-11	0.60	MFS multidrug transporter, putative (*A. benhamiae*)
TERG_01481	E004	3.13E-50	0.54	MFS multidrug transporter, putative (*A. benhamiae*)
TERG_01495	E001	3.04E-05	0.11	COP9 signalosome complex subunit 12 (*T. equinum*)
TERG_01501	E001	9.53E-03	0.11	hypothetical protein
TERG_01501	E002	1.99E-02	-0.15	hypothetical protein
TERG_01510	E013	4.18E-02	-0.28	proline-specific permease (*T. tonsurans*)
TERG_01556	E001	1.33E-02	-0.13	CaaX farnesyltransferase alpha subunit (*T. tonsurans*)
TERG_01556	E002	2.70E-03	-0.14	CaaX farnesyltransferase alpha subunit (*T. tonsurans*)
TERG_01556	E003	4.74E-03	0.32	CaaX farnesyltransferase alpha subunit (*T. tonsurans*)
TERG_01564	E005	3.77E-02	-0.29	pyruvate carboxylase
TERG_01569	E001	4.02E-03	0.11	hypothetical protein
TERG_01569	E002	4.02E-03	-0.11	hypothetical protein
TERG_01599	E001	5.86E-04	-0.22	hypothetical protein
TERG_01599	E002	1.96E-02	-0.15	hypothetical protein
TERG_01599	E003	3.44E-05	-0.21	hypothetical protein
TERG_01599	E005	2.60E-02	0.20	hypothetical protein
TERG_01599	E006	2.34E-28	1.47	hypothetical protein
TERG_01600	E005	4.12E-02	0.11	cytochrome c oxidase-assembly factor COX23, mitochondrial
TERG_01685	E001	2.93E-02	-0.12	glutathione S-transferase, putative (*A. benhamiae*)
TERG_01685	E002	2.88E-02	0.10	glutathione S-transferase, putative (*A. benhamiae*)
TERG_01718	E003	2.40E-02	-0.57	cell division control protein Cdc6 (*T. tonsurans*)
TERG_01764	E002	5.67E-04	0.86	rab6 (*T. equinum*)
TERG_01764	E004	1.27E-07	1.00	rab6 (*T. equinum*)
TERG_01764	E006	1.95E-10	0.94	rab6 (*T. equinum*)
TERG_01819	E001	3.67E-10	0.33	protein CFT1
TERG_01819	E007	5.14E-06	-0.18	protein CFT1
TERG_01877	E001	1.52E-02	0.08	FAM96B (*M. canis*)
TERG_01877	E002	3.13E-02	0.23	FAM96B (*M. canis*)
TERG_01877	E004	2.53E-02	-0.08	FAM96B (*M. canis*)
TERG_01894	E001	7.86E-03	-0.14	hypothetical protein
TERG_01894	E002	8.56E-03	0.17	hypothetical protein
TERG_01936	E001	1.61E-213	-0.41	COP9 signalosome complex subunit 1 (*T. equinum*)
TERG_01936	E002	1.80E-12	0.42	COP9 signalosome complex subunit 1 (*T. equinum*)
TERG_01936	E004	8.57E-09	0.51	COP9 signalosome complex subunit 1 (*T. equinum*)

TERG_01936	E005	2.87E-14	0.45	COP9 signalosome complex subunit 1 (*T. equinum*)
TERG_01936	E006	1.13E-12	0.63	COP9 signalosome complex subunit 1 (*T. equinum*)
TERG_01938	E001	2.63E-17	0.74	hypothetical protein
TERG_01938	E004	1.66E-04	-0.29	hypothetical protein
TERG_01938	E005	6.03E-04	-0.30	hypothetical protein
TERG_01959	E001	6.14E-05	0.19	RNA polymerase Rpb1 C-terminal repeat domain-containing protein (*T. equinum*)
TERG_01959	E002	4.92E-05	-0.12	RNA polymerase Rpb1 C-terminal repeat domain-containing protein (*T. equinum*)
TERG_01972	E001	2.92E-09	0.57	hypothetical protein
TERG_01972	E004	2.40E-02	-0.04	hypothetical protein
TERG_01972	E005	5.06E-03	-0.11	hypothetical protein
TERG_02075	E001	1.54E-17	0.24	T-complex protein 1 subunit delta
TERG_02189	E005	2.17E-05	-0.06	tubulin alpha-2 chain
TERG_02189	E010	8.76E-04	0.17	tubulin alpha-2 chain
TERG_02238	E001	4.94E-05	0.32	ATP phosphoribosyltransferase
TERG_02312	E004	5.03E-03	-0.92	sister chromatid cohesion protein Dcc1, putative (*A. benhamiae*)
TERG_02381	E001	1.65E-03	-0.17	vacuolar protein sorting protein 62 (*M. gypseum*)
TERG_02382	E001	1.23E-03	-0.26	septin (*T. tonsurans*)
TERG_02409	E001	2.75E-02	-0.06	ABcox13, cytochrome c oxidase subunit VIa, putative (*A. benhamiae*)
TERG_02409	E003	2.42E-02	-0.02	ABcox13, cytochrome c oxidase subunit VIa, putative (*A. benhamiae*)
TERG_02409	E004	1.95E-17	0.59	ABcox13, cytochrome c oxidase subunit VIa, putative (*A. benhamiae*)
TERG_02423	E001	8.82E-03	-0.22	hypothetical protein
TERG_02425	E001	8.24E-16	-0.36	RNA binding protein (*A. benhamiae*)
TERG_02425	E002	9.22E-03	0.15	RNA binding protein (*A. benhamiae*)
TERG_02425	E004	1.05E-06	0.16	RNA binding protein (*A. benhamiae*)
TERG_02425	E005	1.56E-03	0.12	RNA binding protein (*A. benhamiae*)
TERG_02443	E002	2.41E-02	0.19	hypothetical protein
TERG_02478	E003	5.73E-04	-0.13	26S proteasome non-ATPase regulatory subunit 3 (*T. equinum*)
TERG_02488	E001	2.01E-07	0.15	hypothetical protein
TERG_02488	E002	7.09E-07	-0.55	hypothetical protein
TERG_02531	E004	3.18E-02	-0.18	GTPase (*T. tonsurans*)
TERG_02545	E007	2.45E-02	0.48	MFS monocarboxylate transporter (*T. equinum*)
TERG_02545	E009	6.84E-06	0.46	MFS monocarboxylate transporter (*T. equinum*)
TERG_02581	E001	1.72E-03	0.26	transmembrane protein UsgS (*T. tonsurans*)
TERG_02594	E001	2.41E-04	-0.38	phosphotransferase (*T. tonsurans*)
TERG_02594	E002	2.39E-04	0.35	phosphotransferase (*T. tonsurans*)
TERG_02601	E006	6.13E-05	-2.32	Phosphoglucomutase (*T. tonsurans*)
TERG_02654	E006	5.37E-03	6.93	membrane transporter (*T. equinum*)
TERG_02694	E004	4.67E-03	-1.21	sulfite efflux pump SSU1
TERG_02848	E001	3.21E-04	-0.69	short chain dehydrogenase/reductase family protein (*A. benhamiae*)
TERG_02853	E001	1.37E-02	-0.05	WD repeat containing protein pop3 (*T. tonsurans*)
TERG_02853	E008	2.19E-03	0.11	WD repeat containing protein pop3 (*T. tonsurans*)
TERG_02878	E003	5.87E-03	0.51	eukaryotic translation initiation factor 3 subunit F
TERG_02922	E001	1.64E-02	-0.82	phosphoserine phosphatase (*T. tonsurans*)

TERG_02939	E005	8.20E-03	6.58	Ser/Thr protein phosphatase (*T. tonsurans*)
TERG_02977	E005	1.02E-02	0.52	hypothetical protein
TERG_02993	E001	8.77E-05	0.73	allergenic cerato-platanin Asp F13 (*T. verrucosum*)
TERG_02993	E003	2.20E-03	-0.09	allergenic cerato-platanin Asp F13 (*T. verrucosum*)
TERG_03009	E002	7.15E-03	0.81	hypothetical protein
TERG_03038	E003	4.64E-03	-0.15	translation elongation factor Tu
TERG_03065	E004	1.82E-03	-0.35	NADP-dependent malic enzyme MaeA (*T. tonsurans*)
TERG_03078	E005	5.81E-05	-1.40	cytochrome P450 oxidoreductase OrdA-like, putative (*T. verrucosum*)
TERG_03078	E007	3.70E-04	-2.07	cytochrome P450 oxidoreductase OrdA-like, putative (*T. verrucosum*)
TERG_03078	E009	6.84E-06	-1.57	cytochrome P450 oxidoreductase OrdA-like, putative (*T. verrucosum*)
TERG_03078	E013	2.02E-03	-3.01	cytochrome P450 oxidoreductase OrdA-like, putative (*T. verrucosum*)
TERG_03081	E004	3.32E-02	-0.32	intracellular protein transporter (*T. equinum*)
TERG_03094	E001	1.64E-02	-0.25	deacetylase complex subunit Sds3 (*T. tonsurans*)
TERG_03094	E002	9.63E-03	0.05	deacetylase complex subunit Sds3 (*T. tonsurans*)
TERG_03118	E001	1.85E-02	-0.15	transcriptional corepressor of histone genes (Hir3), putative (*A. benhamiae*)
TERG_03124	E003	4.53E-02	0.33	histone
TERG_03190	E001	1.22E-02	0.07	NADPH oxidase regulator NoxR (*T. equinum*)
TERG_03194	E001	4.10E-03	-0.17	nonribosomal peptide synthase, putative (*A. benhamiae*)
TERG_03194	E002	5.09E-03	0.25	nonribosomal peptide synthase, putative (*A. benhamiae*)
TERG_03211	E003	1.01E-06	0.47	actin cytoskeleton protein (*T. tonsurans*)
TERG_03286	E006	6.75E-03	-1.62	vacuolar transporter chaperone 4 (*T. tonsurans*)
TERG_03338	E005	6.25E-03	-0.14	acylphosphatase (*T. tonsurans*)
TERG_03340	E005	1.51E-03	0.42	oxidoreductase (*T. equinum*)
TERG_03340	E006	1.58E-02	0.46	oxidoreductase (*T. equinum*)
TERG_03373	E004	4.12E-02	-1.10	TAM domain methyltransferase, putative (*A. benhamiae*)
TERG_03441	E001	4.76E-02	-1.28	hypothetical protein
TERG_03441	E003	1.48E-03	0.19	hypothetical protein
TERG_03494	E008	4.61E-02	1.17	hypothetical protein
TERG_03506	E001	4.12E-02	-0.18	hypothetical protein
TERG_03506	E002	3.06E-02	0.02	hypothetical protein
TERG_03516	E002	1.72E-04	0.53	mitochondrial dicarboxylate carrier (*T. tonsurans*)
TERG_03517	E005	5.94E-07	-0.19	cytochrome c oxidase subunit VIb (*T. tonsurans*)
TERG_03528	E005	2.25E-02	-0.01	cytochrome c
TERG_03572	E003	4.72E-03	0.09	hypothetical protein
TERG_03654	E001	4.25E-02	0.28	NADH-cytochrome b5 reductase 1
TERG_03654	E003	6.43E-04	-0.02	NADH-cytochrome b5 reductase 1
TERG_03699	E001	3.62E-04	0.24	hypothetical protein
TERG_03699	E003	1.21E-09	-1.19	hypothetical protein
TERG_03706	E001	2.50E-05	0.32	trichodiene oxygenase (*T. equinum*)
TERG_03719	E001	6.96E-03	0.06	MFS sugar transporter (*T. tonsurans*)
TERG_03719	E002	7.12E-03	-0.08	MFS sugar transporter (*T. tonsurans*)
TERG_03727	E001	4.27E-02	0.11	hypothetical protein
TERG_03736	E007	2.71E-02	-0.29	transcriptional corepressor Cyc8 (*T. equinum*)

TERG_03818	E002	3.64E-03	-0.19	import receptor (*T. tonsurans*)
TERG_03821	E007	1.30E-02	-0.18	SLY1 (*T. equinum*)
TERG_03875	E002	1.19E-06	0.35	glycogen phosphorylase (*T. equinum*)
TERG_03875	E003	6.14E-28	1.57	glycogen phosphorylase (*T. equinum*)
TERG_03905	E001	2.45E-02	0.28	tubulin gamma chain
TERG_03933	E004	1.10E-03	0.97	ABC metal ion transporter (*T. equinum*)
TERG_03933	E008	1.77E-02	0.91	ABC metal ion transporter (*T. equinum*)
TERG_03981	E007	1.01E-06	0.14	hypothetical protein
TERG_04073	E006	5.35E-13	0.27	glutathione synthetase
TERG_04077	E007	1.09E-09	0.17	domain membrane protein (*M. gypseum*)
TERG_04079	E001	2.80E-02	-0.08	4-aminobutyrate aminotransferase
TERG_04079	E004	9.41E-04	0.03	4-aminobutyrate aminotransferase
TERG_04095	E005	3.31E-02	0.33	hypothetical protein
TERG_04107	E002	1.41E-04	0.38	FunK1 protein kinase (*M. canis*)
TERG_04145	E010	1.95E-17	0.71	ATP synthase subunit beta, mitochondrial
TERG_04234	E001	3.30E-07	0.02	hydrophobin, putative (*T. verrucosum*)
TERG_04269	E001	7.07E-03	0.16	hypothetical protein
TERG_04312	E001	6.70E-09	0.24	ferric-chelate reductase (*T. tonsurans*)
TERG_04312	E005	6.09E-03	-0.32	ferric-chelate reductase (*T. tonsurans*)
TERG_04312	E006	1.16E-02	-0.27	ferric-chelate reductase (*T. tonsurans*)
TERG_04411	E005	5.06E-03	0.03	dipeptidase (*T. equinum*)
TERG_04457	E002	8.56E-03	0.10	POS9-activating factor FAP7 (*T. tonsurans*)
TERG_04457	E003	3.94E-03	-0.17	POS9-activating factor FAP7 (*T. tonsurans*)
TERG_04458	E002	5.56E-03	0.14	RdgB/HAM1 family non-canonical purine NTP pyrophosphatase
TERG_04458	E004	2.00E-04	-0.14	RdgB/HAM1 family non-canonical purine NTP pyrophosphatase
TERG_04458	E005	4.25E-02	-0.13	RdgB/HAM1 family non-canonical purine NTP pyrophosphatase
TERG_04547	E001	3.43E-07	0.12	aquaporin (*T. tonsurans*)
TERG_04547	E002	2.41E-02	-0.22	aquaporin (*T. tonsurans*)
TERG_04580	E006	4.58E-03	-0.85	NADP-specific glutamate dehydrogenase
TERG_04606	E001	4.34E-03	-0.36	general amino acid permease AGP2 (*T. equinum*)
TERG_04621	E001	7.95E-31	0.90	protein transporter SEC13
TERG_04651	E001	1.71E-05	0.24	NADH-ubiquinone oxidoreductase 19.3 kDa subunit, mitochondrial
TERG_04651	E003	2.25E-02	-0.10	NADH-ubiquinone oxidoreductase 19.3 kDa subunit, mitochondrial
TERG_04651	E004	5.00E-04	-0.05	NADH-ubiquinone oxidoreductase 19.3 kDa subunit, mitochondrial
TERG_04651	E006	4.48E-16	-0.03	NADH-ubiquinone oxidoreductase 19.3 kDa subunit, mitochondrial
TERG_04655	E004	2.86E-02	-0.23	ribosomal RNA methyltransferase MRM2 (*T. equinum*)
TERG_04655	E007	2.75E-04	0.40	ribosomal RNA methyltransferase MRM2 (*T. equinum*)
TERG_04656	E002	1.72E-02	-0.59	ubiquitin-like modifier-activating enzyme ATG7
TERG_04664	E001	9.91E-03	-0.16	retinoblastoma-binding protein (*T. equinum*)
TERG_04664	E003	9.88E-03	0.18	retinoblastoma-binding protein (*T. equinum*)
TERG_04684	E002	1.99E-02	-0.15	shugoshin family protein (*A. benhamiae*)
TERG_04684	E003	4.77E-03	0.15	shugoshin family protein (*A. benhamiae*)
TERG_04743	E002	9.53E-03	-0.10	C6 finger domain protein, putative (*A. benhamiae*)
TERG_04743	E006	3.40E-08	0.12	C6 finger domain protein, putative (*A. benhamiae*)

TERG_04754	E001	3.79E-02	0.10	UDP-N-acetyl-glucosamine-1-P transferase (*T. tonsurans*)
TERG_04769	E001	2.55E-02	-0.17	extracellular serine carboxypeptidase, putative (*T. verrucosum*)
TERG_04805	E002	2.71E-02	-0.59	MFS multidrug transporter, putative (*T. verrucosum*)
TERG_04852	E001	4.34E-03	0.04	U-box domain-containing protein (*T. tonsurans*)
TERG_04852	E004	8.56E-03	-0.04	U-box domain-containing protein (*T. tonsurans*)
TERG_04931	E002	1.43E-02	-0.41	allantoate permease (*T. equinum*)
TERG_05069	E004	4.75E-08	0.33	GMF family protein (*T. equinum*)
TERG_05090	E003	2.02E-05	0.82	rho-gdp dissociation inhibitor (*T. equinum*)
TERG_05090	E004	2.45E-02	0.68	rho-gdp dissociation inhibitor (*T. equinum*)
TERG_05090	E006	8.62E-03	0.77	rho-gdp dissociation inhibitor (*T. equinum*)
TERG_05090	E007	5.91E-03	-0.04	rho-gdp dissociation inhibitor (*T. equinum*)
TERG_05090	E008	2.14E-09	0.86	rho-gdp dissociation inhibitor (*T. equinum*)
TERG_05161	E001	1.68E-04	-0.16	26S proteasome regulatory subunit rpn-1
TERG_05161	E006	1.71E-07	0.05	26S proteasome regulatory subunit rpn-1
TERG_05197	E001	5.22E-03	-0.32	protein kinase activator (*T. tonsurans*)
TERG_05208	E001	1.82E-03	0.11	transcription factor iws1
TERG_05245	E001	4.24E-05	0.35	hypothetical protein
TERG_05245	E003	2.73E-07	-0.25	hypothetical protein
TERG_05299	E001	2.77E-04	-0.13	glutathione S-transferase, putative (*A. benhamiae*)
TERG_05299	E002	1.26E-03	0.52	glutathione S-transferase, putative (*A. benhamiae*)
TERG_05333	E001	1.71E-03	0.06	DDT domain-containing protein (*T. tonsurans*)
TERG_05333	E002	1.83E-03	-0.09	DDT domain-containing protein (*T. tonsurans*)
TERG_05361	E001	2.78E-03	0.08	cript family protein (*T. tonsurans*)
TERG_05361	E003	2.19E-03	-0.65	cript family protein (*T. tonsurans*)
TERG_05382	E001	8.56E-03	0.10	sorting nexin Mvp1 (*T. tonsurans*)
TERG_05398	E001	2.33E-02	-0.13	SNF7 family protein (*T. tonsurans*)
TERG_05398	E002	1.89E-02	0.05	SNF7 family protein (*T. tonsurans*)
TERG_05424	E001	2.74E-02	0.27	hypothetical protein
TERG_05452	E001	6.06E-03	0.74	GABA permease (*T. tonsurans*)
TERG_05484	E006	1.43E-03	-1.56	acyl-CoA dehydrogenase (*T. tonsurans*)
TERG_05484	E008	1.12E-05	-0.42	acyl-CoA dehydrogenase (*T. tonsurans*)
TERG_05509	E002	4.40E-02	0.53	aldehyde dehydrogenase ALDH (*A. benhamiae*)
TERG_05540	E001	9.81E-03	0.46	cytochrome P450 monooxygenase, putative (*T. verrucosum*)
TERG_05562	E003	2.25E-02	0.19	2-hydroxychromene-2-carboxylate isomerase, putative (*A. benhamiae*)
TERG_05562	E006	5.56E-03	-0.79	2-hydroxychromene-2-carboxylate isomerase, putative (*A. benhamiae*)
TERG_05569	E005	1.34E-09	-0.61	hypothetical protein
TERG_05569	E006	1.82E-12	-1.28	hypothetical protein
TERG_05575	E001	1.67E-02	0.03	MFS multidrug transporter (*T. tonsurans*)
TERG_05575	E006	3.58E-02	-0.20	MFS multidrug transporter (*T. tonsurans*)
TERG_05576	E001	7.83E-04	-0.04	cell wall glucanase (Scw11), putative (*A. benhamiae*)
TERG_05576	E002	9.26E-04	0.10	cell wall glucanase (Scw11), putative (*A. benhamiae*)
TERG_05647	E004	3.77E-02	0.08	protein kinase activator Bem1 (*T. equinum*)
TERG_05659	E008	3.69E-02	0.33	hypothetical protein
TERG_05673	E003	2.68E-02	0.45	PaaI-thioesterase (*T. equinum*)

TERG_05679	E006	3.80E-02	-0.75	3-hydroxyacyl-CoA dehydrogenase, putative (*A. benhamiae*)
TERG_05703	E001	4.83E-02	0.24	ATP-dependent RNA helicase A (*M. gypseum*)
TERG_05703	E002	3.42E-02	-0.04	ATP-dependent RNA helicase A (*M. gypseum*)
TERG_05787	E001	6.93E-03	0.25	COP9 signalosome complex subunit 12 (*T. equinum*)
TERG_05808	E001	1.05E-04	-0.08	hypothetical protein
TERG_05808	E004	1.91E-05	0.15	hypothetical protein
TERG_05846	E008	1.61E-02	0.19	G1/S-specific cyclin CLN1 (*T. equinum*)
TERG_05885	E004	1.94E-04	0.78	DnaJ domain-containing protein (*T. equinum*)
TERG_05885	E007	3.98E-04	0.00	DnaJ domain-containing protein (*T. equinum*)
TERG_05885	E009	1.07E-02	-0.19	DnaJ domain-containing protein (*T. equinum*)
TERG_05892	E002	1.39E-02	0.07	hypothetical protein
TERG_05892	E003	3.85E-03	-0.10	hypothetical protein
TERG_05925	E008	1.35E-07	0.66	EF-hand protein (*T. tonsurans*)
TERG_05930	E003	3.42E-02	-0.70	5-formyltetrahydrofolate cyclo-ligase
TERG_05957	E001	3.42E-02	0.08	hypothetical protein
TERG_05971	E004	9.53E-03	-0.09	ribonucleoside-diphosphate reductase small chain
TERG_05988	E001	3.84E-02	-0.22	solid-state culture expressed protein (Aos23), putative (*T. verrucosum*)
TERG_06004	E001	1.38E-02	0.06	hypothetical protein
TERG_06004	E002	1.99E-02	-0.25	hypothetical protein
TERG_06005	E003	4.29E-04	-0.47	hypothetical protein
TERG_06075	E006	2.64E-02	0.21	isocitrate dehydrogenase [NADP], mitochondrial
TERG_06075	E007	2.47E-09	0.89	isocitrate dehydrogenase [NADP], mitochondrial
TERG_06075	E008	1.50E-03	0.20	isocitrate dehydrogenase [NADP], mitochondrial
TERG_06088	E002	2.29E-02	-0.10	PH domain-containing protein (*T. equinum*)
TERG_06089	E002	2.50E-02	-1.18	PH domain-containing protein (*T. equinum*)
TERG_06089	E007	4.02E-03	-0.29	PH domain-containing protein (*T. equinum*)
TERG_06095	E001	4.05E-02	-0.09	hypothetical protein
TERG_06095	E002	4.44E-02	0.12	hypothetical protein
TERG_06098	E002	1.89E-02	-0.12	selenoprotein W family protein (*T. equinum*)
TERG_06098	E003	2.66E-02	0.21	selenoprotein W family protein (*T. equinum*)
TERG_06149	E001	1.09E-06	0.10	NIMA interactive protein (*T. equinum*)
TERG_06149	E003	1.97E-04	-0.34	NIMA interactive protein (*T. equinum*)
TERG_06220	E001	1.41E-03	0.28	hypothetical protein
TERG_06220	E002	6.09E-04	-0.06	hypothetical protein
TERG_06234	E002	8.21E-04	-0.70	2,5-diketo-D-gluconic acid reductase A (*T. equinum*)
TERG_06270	E004	7.15E-03	-0.24	hypothetical protein
TERG_06288	E007	1.36E-02	1.64	FACT complex subunit pob3
TERG_06288	E009	1.00E-02	1.31	FACT complex subunit pob3
TERG_06296	E003	4.57E-02	-0.37	hypothetical protein
TERG_06327	E003	3.11E-04	-1.20	integral membrane protein (*T. equinum*)
TERG_06328	E001	4.94E-02	0.08	AdoMet-dependent rRNA methyltransferase spb1
TERG_06328	E002	4.46E-02	-0.04	AdoMet-dependent rRNA methyltransferase spb1
TERG_06330	E002	2.80E-02	0.15	C6 zinc finger domain containing protein (*T. equinum*)
TERG_06344	E007	5.70E-03	-0.34	V-type proton ATPase subunit D
TERG_06493	E005	3.94E-02	-0.23	mRNA cleavage factor complex component Pcf11 (*T. equinum*)
TERG_06505	E006	2.87E-02	0.62	hsp70-like protein

TERG_06513	E008	6.29E-03	0.26	chromosome segregation protein (*T. tonsurans*)
TERG_06522	E003	4.83E-02	-0.41	FMN dependent dehydrogenase, putative (*T. verrucosum*)
TERG_06528	E001	5.82E-05	0.20	Delta(24(24(1)))-sterol reductase (*T. equinum*)
TERG_06528	E002	8.00E-04	-0.25	Delta(24(24(1)))-sterol reductase (*T. equinum*)
TERG_06552	E007	1.26E-05	0.68	aspartic-type endopeptidase (OpsB), putative (*T. verrucosum*)
TERG_06565	E003	4.94E-03	0.19	protein kinase subdomain-containing protein (*T. equinum*)
TERG_06565	E005	4.05E-02	-0.17	protein kinase subdomain-containing protein (*T. equinum*)
TERG_06618	E001	1.51E-03	0.03	mitochondrial GTPase (*T. tonsurans*)
TERG_06618	E002	3.46E-03	-0.38	mitochondrial GTPase (*T. tonsurans*)
TERG_06622	E001	2.08E-03	-0.17	hydrolase (*T. tonsurans*)
TERG_06652	E003	8.62E-03	-0.32	Amt4 (*T. equinum*)
TERG_06668	E006	2.88E-02	0.09	DUF221 domain-containing protein (*T. equinum*)
TERG_06686	E001	1.92E-03	0.04	hypothetical protein
TERG_06686	E007	2.47E-12	-0.89	hypothetical protein
TERG_06725	E001	9.08E-04	0.44	non-histone chromosomal protein 6
TERG_06749	E001	4.38E-09	-0.53	cell wall protein, putative (*A. benhamiae*)
TERG_06749	E002	2.09E-18	-0.40	cell wall protein, putative (*A. benhamiae*)
TERG_06749	E003	4.23E-15	0.25	cell wall protein, putative (*A. benhamiae*)
TERG_06759	E003	1.40E-02	-0.36	C2H2 transcription factor (Seb1), putative (*A. benhamiae*)
TERG_06844	E002	1.42E-03	-0.66	40S ribosomal protein S21
TERG_07005	E001	6.69E-03	-0.09	PAB-dependent poly(A)-specific ribonuclease subunit PAN3 (*T. tonsurans*)
TERG_07005	E004	4.94E-03	0.09	PAB-dependent poly(A)-specific ribonuclease subunit PAN3 (*T. tonsurans*)
TERG_07058	E003	6.84E-08	-0.30	Hsp70 family chaperone, putative (*A. benhamiae*)
TERG_07058	E004	1.50E-02	0.00	Hsp70 family chaperone, putative (*A. benhamiae*)
TERG_07058	E005	8.42E-04	-0.28	Hsp70 family chaperone, putative (*A. benhamiae*)
TERG_07131	E001	3.39E-04	0.07	phosphotransmitter protein Ypd1 (*T. equinum*)
TERG_07131	E003	1.56E-02	-0.23	phosphotransmitter protein Ypd1 (*T. equinum*)
TERG_07136	E001	9.09E-12	0.19	*A. benhamiae* Rab geranylgeranyltransferase, beta subunit (1826 nt)
TERG_07136	E005	3.01E-02	-0.24	*A. benhamiae* Rab geranylgeranyltransferase, beta subunit (1826 nt)
TERG_07136	E006	9.91E-03	-0.15	*A. benhamiae* Rab geranylgeranyltransferase, beta subunit (1826 nt)
TERG_07143	E001	6.82E-12	-0.56	potassium/sodium efflux P-type ATPase, fungal-type (3762 nt)
TERG_07159	E008	1.60E-05	0.24	*T. verrucosum* geranylgeranyl transferae type II alpha subunit, putative (1295 nt)
TERG_07241	E001	1.26E-03	0.21	saccharopine dehydrogenase [NADP+, L-glutamate-forming] (2357 nt)
TERG_07274	E001	1.19E-02	0.02	hypothetical protein (3330 nt)
TERG_07406	E007	4.95E-02	0.28	*T. tonsurans* alpha-mannosidase (2057 nt)
TERG_07431	E002	1.15E-02	-0.80	*A. benhamiae* DnaJ domain protein (3397 nt)
TERG_07457	E001	3.55E-03	-0.55	histidinol-phosphate transaminase (3837 nt)
TERG_07457	E005	2.40E-07	0.23	histidinol-phosphate transaminase (3837 nt)
TERG_07468	E001	2.01E-10	0.19	hypothetical protein (2508 nt)
TERG_07468	E003	1.95E-10	-0.24	hypothetical protein (2508 nt)
TERG_07534	E004	4.28E-05	-0.31	NADH-ubiquinone oxidoreductase complex 1/LYR family protein (*M. gypseum*)

TERG_07583	E001	2.67E-02	0.35	methionyl-tRNA formyltransferase (*T. tonsurans*)
TERG_07630	E003	4.62E-02	0.57	FMN dependent dehydrogenase, putative (*T. verrucosum*)
TERG_07658	E004	1.27E-02	1.06	hsp88-like protein (*T. tonsurans*)
TERG_07658	E010	1.45E-02	0.51	hsp88-like protein (*T. tonsurans*)
TERG_07785	E002	3.42E-05	0.13	SNARE domain-containing protein (*T. tonsurans*)
TERG_07785	E003	8.57E-09	-0.32	SNARE domain-containing protein (*T. tonsurans*)
TERG_07814	E001	4.35E-02	0.09	isocitrate dehydrogenase, NAD-dependent
TERG_07834	E001	1.99E-02	-0.36	alpha-actinin (*T. equinum*)
TERG_07834	E002	8.76E-04	-0.17	alpha-actinin (*T. equinum*)
TERG_07834	E004	2.98E-07	0.09	alpha-actinin (*T. equinum*)
TERG_07868	E001	4.67E-02	-0.66	V-type proton ATPase proteolipid subunit 2
TERG_07916	E001	2.54E-02	0.43	calcium-translocating P-type ATPase, PMCA-type
TERG_07919	E001	3.57E-11	-1.01	thioesterase family protein (*T. verrucosum*)
TERG_07919	E003	3.30E-04	0.07	thioesterase family protein (*T. verrucosum*)
TERG_07937	E001	1.85E-15	-0.47	G-patch domain-containing protein (*T. equinum*)
TERG_07937	E002	2.57E-16	0.08	G-patch domain-containing protein (*T. equinum*)
TERG_07943	E001	2.38E-02	0.09	hypothetical protein
TERG_07943	E002	1.81E-02	-0.04	hypothetical protein
TERG_07997	E008	1.44E-02	-0.38	acetamidase (*T. tonsurans*)
TERG_07999	E001	2.50E-05	-0.18	hypothetical protein
TERG_07999	E002	4.94E-05	0.39	hypothetical protein
TERG_08031	E001	1.85E-04	0.13	PHD finger domain-containing protein (*T. equinum*)
TERG_08038	E001	5.59E-09	0.25	polysaccharide deacetylase family protein (*T. equinum*)
TERG_08038	E004	1.67E-12	-0.52	polysaccharide deacetylase family protein (*T. equinum*)
TERG_08041	E001	2.68E-02	0.09	aminotransferase, putative (*A. benhamiae*)
TERG_08083	E001	4.91E-02	0.16	importin subunit beta-2 (*T. equinum*)
TERG_08083	E003	9.91E-03	-0.06	importin subunit beta-2 (*T. equinum*)
TERG_08120	E001	5.94E-03	-0.19	glucose-inducible SAM-dependent methyltransferase Rrg1 (*T. equinum*)
TERG_08152	E001	3.42E-05	0.14	O-methyltransferase (*T. equinum*)
TERG_08152	E002	2.38E-03	-0.18	O-methyltransferase (*T. equinum*)
TERG_08152	E004	4.72E-10	-0.16	O-methyltransferase (*T. equinum*)
TERG_08191	E001	2.95E-02	0.28	glucooligosaccharide oxidase (*T. equinum*)
TERG_08200	E001	1.46E-02	1.36	hypothetical protein
TERG_08255	E001	2.18E-05	-0.10	carboxypeptidase S1, putative (*A. benhamiae*)
TERG_08255	E002	4.55E-07	0.17	carboxypeptidase S1, putative (*A. benhamiae*)
TERG_08288	E002	1.03E-02	-0.08	ATP-citrate synthase subunit 1
TERG_08299	E001	2.01E-06	-0.44	multidrug resistance-associated protein 1 transporter (*M. canis*)
TERG_08304	E001	1.94E-02	0.05	hypothetical protein
TERG_08304	E002	3.20E-02	-0.29	hypothetical protein
TERG_08329	E001	3.99E-06	-0.07	flavin containing amine oxidase (*T. tonsurans*)
TERG_08329	E003	1.64E-06	0.47	flavin containing amine oxidase (*T. tonsurans*)
TERG_08340	E002	5.09E-03	-0.92	F-box domain-containing protein (*T. equinum*)
TERG_08341	E002	3.83E-02	-0.21	hypothetical protein
TERG_08353	E004	5.47E-03	-1.05	cytochrome P450 55A3 (*T. tonsurans*)
TERG_08353	E011	4.08E-02	-0.74	cytochrome P450 55A3 (*T. tonsurans*)

TERG_08355	E005	8.27E-03	-0.26	zinc alcohol dehydrogenase, putative (*T. verrucosum*)
TERG_08378	E003	8.10E-03	-0.10	hypothetical protein
TERG_08405	E004	3.20E-02	-0.09	leucine aminopeptidase 2
TERG_08473	E003	4.66E-02	0.10	hypothetical protein
TERG_08481	E002	6.93E-03	0.10	ubiquitin C-terminal hydrolase (*T. tonsurans*)
TERG_08481	E003	3.14E-03	-0.09	ubiquitin C-terminal hydrolase (*T. tonsurans*)
TERG_08555	E004	3.66E-03	-0.26	nuclear pore protein (*T. equinum*)
TERG_08557	E001	2.24E-02	0.07	carboxypeptidase S1, putative (*A. benhamiae*)
TERG_08615	E004	1.86E-02	-0.30	MFS transporter, putative (*T. verrucosum*)
TERG_08771	E004	3.89E-02	-0.12	extracellular serine-threonine rich protein (*T. tonsurans*)
TERG_08884	E004	1.20E-02	-0.16	GPI-anchored cell surface glycoprotein, putative (*T. verrucosum*)
TERG_08884	E005	3.70E-02	-0.41	GPI-anchored cell surface glycoprotein, putative (*T. verrucosum*)
TERG_08884	E006	1.02E-02	-0.60	GPI-anchored cell surface glycoprotein, putative (*T. verrucosum*)
TERG_08906	E001	3.70E-02	0.34	hypothetical protein
TERG_08906	E002	2.26E-02	-0.13	hypothetical protein
TERG_08980	E001	1.81E-02	1.32	hypothetical protein
TERG_08982	E001	5.73E-04	0.24	hypothetical protein
TERG_08982	E002	4.16E-04	-0.19	hypothetical protein
TERG_11512	E001	2.42E-02	0.10	General negative regulator of transcription subunit 4 (*T. tonsurans*)
TERG_11540	E001	2.71E-04	-0.06	hypothetical protein
TERG_11540	E002	8.63E-04	0.54	hypothetical protein
TERG_11660	E001	4.07E-03	0.10	mitochondrial 40S ribosomal protein (*T. tonsurans*)
TERG_11660	E002	1.34E-07	0.11	mitochondrial 40S ribosomal protein (*T. tonsurans*)
TERG_11660	E003	2.73E-07	0.19	mitochondrial 40S ribosomal protein (*T. tonsurans*)
TERG_11660	E004	6.32E-16	-0.93	mitochondrial 40S ribosomal protein (*T. tonsurans*)
TERG_11660	E005	1.58E-09	-0.70	mitochondrial 40S ribosomal protein (*T. tonsurans*)
TERG_11660	E006	1.16E-27	-0.63	mitochondrial 40S ribosomal protein (*T. tonsurans*)
TERG_11660	E007	9.71E-18	-0.60	mitochondrial 40S ribosomal protein (*T. tonsurans*)
TERG_11688	E001	1.87E-04	-0.18	nitrilase (*T. tonsurans*)
TERG_11787	E001	1.89E-02	-0.10	oligosaccharyl transferase stt3 subunit (*T. equinum*)
TERG_11787	E003	1.73E-02	0.09	oligosaccharyl transferase stt3 subunit (*T. equinum*)
TERG_11862	E001	7.10E-07	-0.44	integral membrane protein (*T. tonsurans*)
TERG_11862	E004	8.06E-03	-0.32	integral membrane protein (*T. tonsurans*)
TERG_11862	E005	5.96E-03	-0.27	integral membrane protein (*T. tonsurans*)
TERG_11862	E007	8.71E-09	0.08	integral membrane protein (*T. tonsurans*)
TERG_11871	E001	2.14E-02	0.46	ubiquitin C-terminal hydrolase 37 (*T. verrucosum*)
TERG_11871	E002	3.64E-03	0.26	ubiquitin C-terminal hydrolase 37 (*T. verrucosum*)
TERG_11871	E005	5.47E-03	-0.36	ubiquitin C-terminal hydrolase 37 (*T. verrucosum*)
TERG_11879	E001	1.38E-02	-0.20	hypothetical protein
TERG_11879	E002	9.89E-03	0.05	hypothetical protein
TERG_11991	E003	6.59E-03	0.75	hypothetical protein

TERG_12000	E007	4.96E-02	0.24	ubiquitin C-terminal hydrolase, putative (*T. verrucosum*)
TERG_12004	E001	9.77E-26	0.18	lipase (*T. tonsurans*)
TERG_12004	E003	9.50E-05	-0.21	lipase (*T. tonsurans*)
TERG_12004	E004	1.86E-04	-0.23	lipase (*T. tonsurans*)
TERG_12012	E001	4.15E-08	-0.35	hypothetical protein
TERG_12012	E003	1.60E-12	0.31	hypothetical protein
TERG_12030	E004	8.97E-03	0.57	DUF821 domain protein (*T. verrucosum*)
TERG_12154	E003	1.75E-02	-0.18	leukotriene A4 hydrolase (*T. verrucosum*)
TERG_12154	E004	7.15E-03	-0.18	leukotriene A4 hydrolase (*T. verrucosum*)
TERG_12154	E006	5.03E-03	-0.26	leukotriene A4 hydrolase (*T. verrucosum*)
TERG_12154	E008	8.45E-08	-0.21	leukotriene A4 hydrolase (*T. verrucosum*)
TERG_12154	E009	1.27E-02	-0.18	leukotriene A4 hydrolase (*T. verrucosum*)
TERG_12154	E014	3.42E-08	0.12	leukotriene A4 hydrolase (*T. verrucosum*)
TERG_12154	E015	1.18E-09	0.16	leukotriene A4 hydrolase (*T. verrucosum*)
TERG_12154	E016	8.16E-11	0.11	leukotriene A4 hydrolase (*T. verrucosum*)
TERG_12154	E017	5.79E-05	0.13	leukotriene A4 hydrolase (*T. verrucosum*)
TERG_12164	E001	1.51E-04	0.47	hypothetical protein
TERG_12164	E003	9.08E-05	-0.12	hypothetical protein
TERG_12234	E004	1.06E-05	0.47	pantoate-beta-alanine ligase
TERG_12308	E004	1.70E-02	-0.23	transcriptional repressor Sin3p (*T. equinum*)
TERG_12335	E005	4.53E-02	0.46	proliferating cell nuclear antigen (pcna)
TERG_12335	E009	3.54E-07	-0.09	proliferating cell nuclear antigen (pcna)
TERG_12351	E001	1.11E-05	-0.90	hypothetical protein
TERG_12351	E002	1.91E-06	0.05	hypothetical protein
TERG_12367	E001	3.67E-10	0.62	60S acidic ribosomal protein P0
TERG_12367	E002	4.71E-10	0.68	60S acidic ribosomal protein P0
TERG_12367	E003	1.74E-12	0.71	60S acidic ribosomal protein P0
TERG_12367	E004	1.01E-08	0.68	60S acidic ribosomal protein P0
TERG_12367	E005	5.00E-02	-0.18	60S acidic ribosomal protein P0
TERG_12367	E006	2.41E-04	-0.21	60S acidic ribosomal protein P0
TERG_12367	E007	1.88E-07	-0.20	60S acidic ribosomal protein P0
TERG_12499	E002	4.64E-02	0.12	2-deoxy-D-gluconate 3-dehydrogenase (*T. equinum*)
TERG_12572	E001	1.53E-02	-0.55	acyl-coenzyme A thioesterase (*T. equinum*)
TERG_12572	E004	3.08E-02	-0.36	acyl-coenzyme A thioesterase (*T. equinum*)
TERG_12572	E005	7.35E-12	-0.50	acyl-coenzyme A thioesterase (*T. equinum*)
TERG_12572	E007	1.73E-02	0.11	acyl-coenzyme A thioesterase (*T. equinum*)
TERG_12572	E009	1.02E-06	0.25	acyl-coenzyme A thioesterase (*T. equinum*)
TERG_12591	E003	1.26E-03	0.14	peptidase S8 family protein (*T. equinum*)
TERG_12591	E004	4.02E-03	-0.45	peptidase S8 family protein (*T. equinum*)
TERG_12680	E001	3.24E-02	0.10	alpha-mannosidase (*T. tonsurans*)
TERG_12680	E002	2.95E-02	-0.07	alpha-mannosidase (*T. tonsurans*)

Total genes = 302, Differential exon usage = 513.
Gene expression values are expressed in log_2 fold change between each time point.

12 hours vs. 0 hour

ID	Exon	padj	12-0 hours	Gene Product Name

TERG_00049	E001	2.22E-03	0.17	hypothetical protein
TERG_00049	E005	1.27E-02	-0.27	hypothetical protein
TERG_00098	E001	1.80E-02	0.28	high affinity methionine permease (*A. benhamiae*)
TERG_00098	E006	1.99E-02	-0.45	high affinity methionine permease (*A. benhamiae*)
TERG_00122	E004	1.30E-02	0.11	ribonuclease P complex subunit Pop1 (*T. tonsurans*)
TERG_00124	E003	8.49E-08	-0.01	peroxisomal matrix protein (*T. equinum*)
TERG_00138	E002	3.80E-02	6.30	tetraspanin (*T. equinum*)
TERG_00152	E001	4.41E-02	0.24	mitochondrial inner membrane magnesium transporter mrs2 (*T. tonsurans*)
TERG_00181	E001	1.34E-03	-0.05	nucleolar protein 58
TERG_00181	E002	1.70E-04	-0.25	nucleolar protein 58
TERG_00181	E005	2.61E-08	0.14	nucleolar protein 58
TERG_00243	E001	3.91E-06	0.79	pathogenesis associated protein Cap20, putative (*T. verrucosum*)
TERG_00250	E003	1.64E-04	2.49	phosphoacetylglucosamine mutase (*T. equinum*)
TERG_00332	E005	4.48E-37	-0.43	nuclear transport factor 2
TERG_00332	E006	3.87E-02	-0.35	nuclear transport factor 2
TERG_00369	E002	4.40E-02	0.11	branchpoint-bridging protein
TERG_00536	E001	4.69E-02	0.12	D-galacturonic acid reductase (*T. equinum*)
TERG_00616	E007	9.77E-05	-0.89	potassium/sodium efflux P-type ATPase, fungal-type
TERG_00659	E002	2.37E-03	-0.87	3-deoxy-7-phosphoheptulonate synthase
TERG_00659	E004	3.89E-03	-1.11	3-deoxy-7-phosphoheptulonate synthase
TERG_00703	E001	3.12E-02	0.12	nuclear migration protein, putative (*A. benhamiae*)
TERG_00703	E002	3.01E-02	-0.09	nuclear migration protein, putative (*A. benhamiae*)
TERG_00706	E001	5.23E-03	0.06	MFS transporter (*T. verrucosum*)
TERG_00706	E002	6.28E-03	-0.18	MFS transporter (*T. verrucosum*)
TERG_00831	E001	4.35E-02	-0.32	gamma-glutamyltransferase
TERG_00831	E003	3.47E-02	-0.13	gamma-glutamyltransferase
TERG_00831	E005	2.29E-03	0.09	gamma-glutamyltransferase
TERG_00841	E004	1.95E-02	-0.14	C6 transcription factor, putative (*T. verrucosum*)
TERG_00849	E005	4.41E-02	-0.40	inner membrane protein yicO (*T. tonsurans*)
TERG_00863	E001	3.50E-03	-0.15	NsdD (*T. equinum*)
TERG_00865	E002	1.68E-02	-0.52	hypothetical protein
TERG_00865	E007	2.83E-06	-0.26	hypothetical protein
TERG_00865	E008	3.31E-02	-0.83	hypothetical protein
TERG_00961	E002	4.44E-02	-0.06	T-complex protein 1, zeta subunit
TERG_01012	E001	2.67E-04	-0.21	hypothetical protein
TERG_01012	E002	7.25E-04	0.73	hypothetical protein
TERG_01012	E003	6.11E-03	0.85	hypothetical protein
TERG_01012	E004	1.59E-02	0.28	hypothetical protein
TERG_01029	E001	2.76E-02	0.07	phosducin family protein (*T. tonsurans*)
TERG_01039	E001	3.11E-02	0.17	DNA topoisomerase III (*T. tonsurans*)
TERG_01048	E001	1.82E-02	0.11	WSC domain protein, putative (*T. verrucosum*)
TERG_01076	E001	1.41E-10	-0.47	aconitate hydratase, mitochondrial
TERG_01076	E007	8.02E-07	0.08	aconitate hydratase, mitochondrial
TERG_01198	E003	7.01E-18	-0.11	pre-mRNA-splicing factor rse1
TERG_01198	E006	8.48E-15	0.28	pre-mRNA-splicing factor rse1
TERG_01254	E003	1.71E-02	0.11	signal sequence receptor alpha subunit (*T. tonsurans*)

TERG_01274	E003	2.61E-04	1.12	mRNA export factor elf1 (*T. equinum*)
TERG_01274	E004	3.36E-02	0.07	mRNA export factor elf1 (*T. equinum*)
TERG_01279	E002	3.78E-03	-0.70	flavin-binding monooxygenase (*T. equinum*)
TERG_01279	E007	1.56E-05	0.28	flavin-binding monooxygenase (*T. equinum*)
TERG_01292	E001	5.13E-03	-0.12	glycosyl hydrolase, putative (*A. benhamiae*)
TERG_01334	E004	5.53E-03	-0.74	C6 finger domain protein, putative (*A. benhamiae*)
TERG_01360	E004	1.51E-04	-1.22	C6 transcription factor, putative (*T. verrucosum*)
TERG_01360	E006	2.01E-03	-1.22	C6 transcription factor, putative (*T. verrucosum*)
TERG_01399	E001	2.74E-02	-1.48	hypothetical protein
TERG_01399	E003	1.06E-02	0.01	hypothetical protein
TERG_01433	E006	4.21E-03	0.12	WD domain, G-beta repeat protein (*A. benhamiae*)
TERG_01443	E010	4.91E-04	-0.12	ABC multidrug transporter (*T. tonsurans*)
TERG_01444	E001	7.36E-03	0.74	nonribosomal peptide synthase Pes1 (*A. benhamiae*)
TERG_01444	E002	4.98E-03	0.91	nonribosomal peptide synthase Pes1 (*A. benhamiae*)
TERG_01444	E003	2.08E-02	1.17	nonribosomal peptide synthase Pes1 (*A. benhamiae*)
TERG_01476	E004	3.21E-02	-0.27	APSES transcription factor Xbp1 (*T. equinum*)
TERG_01481	E004	5.53E-03	0.15	MFS multidrug transporter, putative (*A. benhamiae*)
TERG_01507	E001	1.65E-05	-0.06	extracellular proline-glycine rich protein (*T. verrucosum*)
TERG_01507	E002	4.21E-03	-0.61	extracellular proline-glycine rich protein (*T. verrucosum*)
TERG_01507	E004	1.65E-02	0.11	extracellular proline-glycine rich protein (*T. verrucosum*)
TERG_01522	E001	4.02E-02	-0.05	kinesin heavy chain
TERG_01522	E002	4.64E-02	0.24	kinesin heavy chain
TERG_01599	E003	1.60E-02	-0.28	hypothetical protein
TERG_01599	E006	4.36E-43	1.54	hypothetical protein
TERG_01612	E001	6.44E-03	-0.09	enolase
TERG_01612	E007	2.99E-03	0.04	enolase
TERG_01624	E003	2.00E-03	-0.03	phosphomannomutase (*T. tonsurans*)
TERG_01749	E003	1.13E-02	0.06	hypothetical protein
TERG_01819	E001	1.24E-02	0.16	protein CFT1
TERG_01835	E005	2.98E-02	-0.33	extracellular lipase, putative (*T. verrucosum*)
TERG_01883	E002	3.97E-02	0.04	hsp75-like protein
TERG_01892	E004	2.96E-02	-0.40	alanyl-tRNA synthetase
TERG_01894	E001	6.26E-03	-0.11	hypothetical protein
TERG_01894	E002	6.44E-03	0.17	hypothetical protein
TERG_01905	E001	1.86E-02	-0.07	AMP deaminase
TERG_01905	E002	2.06E-02	0.16	AMP deaminase
TERG_01936	E001	1.79E-03	-0.28	COP9 signalosome complex subunit 1 (*T. equinum*)
TERG_01936	E002	3.45E-03	-0.04	COP9 signalosome complex subunit 1 (*T. equinum*)
TERG_01936	E004	1.80E-02	0.06	COP9 signalosome complex subunit 1 (*T. equinum*)
TERG_01936	E006	1.82E-08	0.19	COP9 signalosome complex subunit 1 (*T. equinum*)
TERG_01959	E001	1.39E-03	0.25	RNA polymerase Rpb1 C-terminal repeat domain-containing protein (*T. equinum*)
TERG_01959	E002	1.30E-03	-0.17	RNA polymerase Rpb1 C-terminal repeat domain-containing protein (*T. equinum*)

TERG_01988	E003	2.01E-03	-0.07	F-box domain protein (*A. benhamiae*)
TERG_02144	E001	3.31E-02	0.08	arsenite resistance protein Ars2 (*T. equinum*)
TERG_02186	E001	8.29E-03	-0.22	ABC multidrug transporter, putative (*A. benhamiae*)
TERG_02186	E002	2.40E-02	0.31	ABC multidrug transporter, putative (*A. benhamiae*)
TERG_02189	E006	9.77E-05	-2.27	tubulin alpha-2 chain
TERG_02210	E003	1.08E-03	0.20	hypothetical protein
TERG_02222	E001	1.51E-02	0.09	TBC domain-containing protein (*T. equinum*)
TERG_02222	E004	6.26E-03	-0.08	TBC domain-containing protein (*T. equinum*)
TERG_02237	E001	9.77E-05	-0.03	40S ribosomal protein S28
TERG_02376	E004	9.13E-10	0.02	60S ribosomal protein (*T. equinum*)
TERG_02418	E001	1.86E-02	-0.05	translation initiation factor SUI1
TERG_02418	E004	9.72E-03	0.04	translation initiation factor SUI1
TERG_02521	E001	1.88E-04	0.03	60S ribosomal protein L27a
TERG_02535	E003	6.62E-04	-0.14	nicotinate-nucleotide diphosphorylase (carboxylating)
TERG_02601	E006	3.04E-04	-7.18	Phosphoglucomutase (*T. tonsurans*)
TERG_02628	E002	3.47E-02	0.15	dihydrolipoamide succinyltransferase (*T. equinum*)
TERG_02629	E005	7.06E-03	-0.40	LYR family protein (*T. equinum*)
TERG_02654	E006	7.74E-03	6.32	membrane transporter (*T. equinum*)
TERG_02719	E005	4.44E-02	-1.85	glycosyl hydrolase (*T. equinum*)
TERG_02735	E008	4.51E-02	0.13	fatty acid oxygenase PpoC, putative (*A. benhamiae*)
TERG_02735	E009	5.23E-03	0.14	fatty acid oxygenase PpoC, putative (*A. benhamiae*)
TERG_02761	E003	1.40E-03	0.13	BUB protein kinase
TERG_02767	E001	4.41E-02	-0.08	pyroglutamyl peptidase type I (*T. equinum*)
TERG_02767	E002	4.40E-02	0.06	pyroglutamyl peptidase type I (*T. equinum*)
TERG_02832	E002	4.92E-02	-0.04	HECT domain protein (*T. verrucosum*)
TERG_02853	E001	1.67E-03	-0.32	WD repeat containing protein pop3 (*T. tonsurans*)
TERG_02853	E007	6.78E-04	0.04	WD repeat containing protein pop3 (*T. tonsurans*)
TERG_02853	E008	4.24E-04	0.07	WD repeat containing protein pop3 (*T. tonsurans*)
TERG_02885	E009	1.07E-03	-0.19	E3 ubiquitin-protein ligase pub1 (*T. tonsurans*)
TERG_02890	E001	1.16E-02	-0.55	rhomboid family protein, putative (*A. benhamiae*)
TERG_02923	E001	1.06E-02	-0.19	protein kinase subdomain-containing protein (*T. equinum*)
TERG_02923	E006	2.95E-02	0.22	protein kinase subdomain-containing protein (*T. equinum*)
TERG_02968	E003	3.37E-02	-1.26	GPI transamidase component Gpi16 (*T. tonsurans*)
TERG_03017	E002	4.05E-02	-0.22	AGC protein kinase
TERG_03038	E003	1.38E-02	-0.14	translation elongation factor Tu
TERG_03067	E001	4.00E-02	-0.43	hypothetical protein
TERG_03067	E002	2.86E-02	0.05	hypothetical protein
TERG_03070	E004	7.74E-03	-0.07	flotillin domain-containing protein (*T. equinum*)
TERG_03070	E006	4.80E-04	0.56	flotillin domain-containing protein (*T. equinum*)
TERG_03070	E007	4.26E-09	0.07	flotillin domain-containing protein (*T. equinum*)
TERG_03082	E005	2.59E-02	0.22	5'-3' exoribonuclease (*T. tonsurans*)
TERG_03083	E001	4.02E-02	-0.05	3-phosphoshikimate 1-carboxyvinyltransferase
TERG_03118	E010	3.67E-02	-0.15	transcriptional corepressor of histone genes (Hir3), putative (*A. benhamiae*)
TERG_03123	E001	1.66E-04	0.01	histone H3

TERG_03373	E001	8.42E-03	0.19	TAM domain methyltransferase, putative (*A. benhamiae*)
TERG_03401	E001	1.05E-02	0.21	high affinity nickel transporter (*T. tonsurans*)
TERG_03441	E003	3.73E-02	0.14	hypothetical protein
TERG_03494	E008	4.33E-02	0.96	hypothetical protein
TERG_03652	E001	1.90E-02	-0.07	chromosome segregation protein sudA
TERG_03652	E003	2.56E-02	0.21	chromosome segregation protein sudA
TERG_03654	E004	2.31E-03	0.08	NADH-cytochrome b5 reductase 1
TERG_03656	E002	1.08E-03	-0.04	arginine-tRNA ligase
TERG_03656	E003	1.32E-02	0.16	arginine-tRNA ligase
TERG_03700	E002	4.82E-02	0.39	hypothetical protein
TERG_03795	E001	3.63E-02	0.08	ubiquitin C-terminal hydrolase, putative (*T. verrucosum*)
TERG_03818	E002	7.27E-03	-0.26	import receptor (*T. tonsurans*)
TERG_03821	E001	4.53E-03	0.11	SLY1 (*T. equinum*)
TERG_03821	E007	1.08E-03	-0.11	SLY1 (*T. equinum*)
TERG_03850	E001	3.12E-02	-1.66	hypothetical protein
TERG_03850	E002	2.35E-02	0.51	hypothetical protein
TERG_03857	E004	1.94E-03	0.75	phosphotransferase enzyme family protein (*T. equinum*)
TERG_03857	E006	5.25E-03	0.32	phosphotransferase enzyme family protein (*T. equinum*)
TERG_03872	E012	4.35E-02	0.21	CMGC/MAPK/P38 protein kinase
TERG_03933	E001	6.26E-03	0.11	ABC metal ion transporter (*T. equinum*)
TERG_03933	E014	2.08E-02	-0.07	ABC metal ion transporter (*T. equinum*)
TERG_03946	E004	8.50E-04	0.11	SH3 domain-containing protein (*T. tonsurans*)
TERG_03967	E001	1.96E-03	0.11	THO complex protein subunit 2 (*T. equinum*)
TERG_03967	E002	7.25E-04	-0.12	THO complex protein subunit 2 (*T. equinum*)
TERG_03981	E007	1.95E-03	0.15	hypothetical protein
TERG_04087	E001	3.05E-03	-0.03	DnaJ domain-containing protein (*T. tonsurans*)
TERG_04142	E005	2.97E-22	-0.50	cofilin (*T. equinum*)
TERG_04142	E009	1.81E-02	0.51	cofilin (*T. equinum*)
TERG_04142	E010	6.44E-03	0.07	cofilin (*T. equinum*)
TERG_04281	E002	1.39E-03	-0.06	woronin body major protein
TERG_04281	E005	2.32E-02	0.04	woronin body major protein
TERG_04301	E002	6.44E-03	0.63	transcription factor RfeG (*T. tonsurans*)
TERG_04450	E001	1.71E-05	0.10	60S ribosomal protein L23 (*T. tonsurans*)
TERG_04450	E004	1.02E-02	-0.03	60S ribosomal protein L23 (*T. tonsurans*)
TERG_04461	E009	4.35E-02	-0.24	P-type ATPase (*T. equinum*)
TERG_04461	E016	2.23E-02	-0.26	P-type ATPase (*T. equinum*)
TERG_04470	E001	4.39E-04	0.15	serine-tRNA ligase
TERG_04478	E002	2.88E-03	0.03	40S ribosomal protein S1
TERG_04490	E004	1.21E-05	-1.60	NADH-ubiquinone oxidoreductase 78 kDa subunit, mitochondrial
TERG_04490	E005	1.96E-02	-0.32	NADH-ubiquinone oxidoreductase 78 kDa subunit, mitochondrial
TERG_04558	E005	4.77E-05	0.07	nucleoside diphosphate kinase
TERG_04851	E001	5.25E-03	0.02	Acyl CoA binding protein family protein (*A. benhamiae*)
TERG_04903	E004	2.74E-02	0.19	thermotolerance protein (*T. equinum*)
TERG_04904	E001	4.40E-02	-0.18	translation initiation factor aIF-2
TERG_04928	E003	2.13E-02	-0.22	elongation factor 2

TERG_04928	E004	1.32E-02	-0.05	elongation factor 2
TERG_04928	E005	2.86E-06	0.02	elongation factor 2
TERG_05050	E001	4.89E-03	0.11	acyltransferase (*T. equinum*)
TERG_05089	E001	2.88E-03	0.02	serine/threonine-protein phosphatase 2A catalytic subunit beta isoform (*T. equinum*)
TERG_05089	E002	1.06E-11	0.63	serine/threonine-protein phosphatase 2A catalytic subunit beta isoform (*T. equinum*)
TERG_05101	E001	8.50E-04	0.09	MYB DNA binding protein (*T. tonsurans*)
TERG_05101	E002	2.22E-02	-0.28	MYB DNA binding protein (*T. tonsurans*)
TERG_05141	E004	4.33E-02	1.14	NCS1 nucleoside transporter (*T. equinum*)
TERG_05161	E006	1.03E-02	0.04	26S proteasome regulatory subunit rpn-1
TERG_05186	E001	3.14E-02	-0.14	RING finger ubiquitin ligase (*T. tonsurans*)
TERG_05236	E001	2.83E-06	0.04	60S ribosomal protein L35
TERG_05261	E003	1.59E-02	-0.09	karyopherin alpha subunit (*T. tonsurans*)
TERG_05475	E003	4.77E-05	-1.88	Got1 family protein (*T. tonsurans*)
TERG_05483	E001	3.63E-02	0.18	transcription elongation factor spt5 (*M. gypseum*)
TERG_05524	E002	4.51E-02	-0.69	3-deoxy-7-phosphoheptulonate synthase
TERG_05532	E005	2.81E-02	-0.26	monosaccharide transporter (*T. tonsurans*)
TERG_05540	E001	4.77E-02	0.51	cytochrome P450 monooxygenase, putative (*T. verrucosum*)
TERG_05558	E001	2.49E-02	0.07	ornithine decarboxylase, putative (*T. verrucosum*)
TERG_05558	E003	5.01E-03	-0.09	ornithine decarboxylase, putative (*T. verrucosum*)
TERG_05579	E003	1.11E-02	-0.10	FAD dependent oxidoreductase, putative (*A. benhamiae*)
TERG_05789	E002	2.35E-02	-0.08	NADH-cytochrome b5 reductase 2
TERG_05789	E003	9.30E-04	0.11	NADH-cytochrome b5 reductase 2
TERG_05821	E013	1.16E-03	0.21	pyruvate kinase
TERG_05829	E001	3.69E-02	-0.08	WD domain, G-beta repeat containing protein (*T. tonsurans*)
TERG_05829	E002	3.90E-02	0.13	WD domain, G-beta repeat containing protein (*T. tonsurans*)
TERG_05971	E006	3.67E-02	-0.18	ribonucleoside-diphosphate reductase small chain
TERG_05992	E001	1.14E-02	-0.20	translational initiation factor 2 beta (*T. tonsurans*)
TERG_06028	E004	4.34E-02	0.08	peptidyl-prolyl cis-trans isomerase-like 4
TERG_06057	E004	4.92E-02	-0.12	C2 domain-containing protein (*T. equinum*)
TERG_06172	E002	3.87E-02	-0.01	60S ribosomal protein L12
TERG_06234	E001	3.58E-02	0.27	2,5-diketo-D-gluconic acid reductase A (*T. equinum*)
TERG_06241	E007	4.91E-04	0.14	importin 11 (*T. equinum*)
TERG_06286	E001	1.32E-02	0.37	C6 finger domain-containing protein (*T. equinum*)
TERG_06288	E009	2.72E-03	1.57	FACT complex subunit pob3
TERG_06293	E004	1.85E-02	-0.16	vacuolar sorting protein (*T. tonsurans*)
TERG_06304	E001	6.63E-03	-0.26	NEDD8-activating enzyme E1 catalytic subunit (*T. equinum*)
TERG_06309	E003	1.00E-02	0.32	oxidoreductase, 2OG-Fe(II) oxygenase family, putative (*A. benhamiae*)
TERG_06340	E003	4.03E-02	-0.20	AGC/PKC protein kinase
TERG_06347	E001	2.83E-06	0.33	hypothetical protein
TERG_06437	E004	1.26E-02	-0.02	ribosomal protein L28e (*T. equinum*)
TERG_06475	E003	3.57E-04	0.08	zinc knuckle domain-containing protein (*T. tonsurans*)
TERG_06505	E002	1.32E-02	-0.02	hsp70-like protein
TERG_06505	E006	3.63E-02	0.64	hsp70-like protein

TERG_06646	E001	4.71E-02	-0.07	arrestin (*T. equinum*)
TERG_06646	E002	4.64E-02	0.04	arrestin (*T. equinum*)
TERG_06656	E004	1.02E-02	0.18	T-complex protein 1 subunit beta
TERG_06704	E001	1.24E-04	-0.14	vacuolar protease A
TERG_06704	E004	1.05E-02	0.05	vacuolar protease A
TERG_06725	E007	6.34E-03	0.04	non-histone chromosomal protein 6
TERG_06735	E001	1.24E-04	-0.21	oxysterol binding protein (*T. tonsurans*)
TERG_06749	E001	7.92E-05	-0.33	cell wall protein, putative (*A. benhamiae*)
TERG_06749	E003	2.32E-02	0.24	cell wall protein, putative (*A. benhamiae*)
TERG_06797	E002	3.67E-02	-0.18	hypothetical protein
TERG_06844	E002	2.26E-39	-1.11	40S ribosomal protein S21
TERG_06846	E001	2.26E-06	0.05	inosine-5'-monophosphate dehydrogenase
TERG_06846	E002	8.95E-06	-0.09	inosine-5'-monophosphate dehydrogenase
TERG_06871	E005	1.10E-02	-0.12	membrane bound C2 domain-containing protein (*T. tonsurans*)
TERG_06871	E006	3.97E-02	-0.10	membrane bound C2 domain-containing protein (*T. tonsurans*)
TERG_06874	E001	2.17E-03	-0.47	UTP-glucose-1-phosphate uridylyltransferase (*T. tonsurans*)
TERG_06874	E003	6.32E-03	0.03	UTP-glucose-1-phosphate uridylyltransferase (*T. tonsurans*)
TERG_06969	E001	4.10E-02	0.09	CMGC/CDK/CDK5 protein kinase
TERG_06995	E001	4.66E-03	0.04	metalloreductase, putative (*T. verrucosum*)
TERG_06995	E003	2.45E-02	-0.30	metalloreductase, putative (*T. verrucosum*)
TERG_07005	E004	3.47E-02	-0.07	PAB-dependent poly(A)-specific ribonuclease subunit PAN3 (*T. tonsurans*)
TERG_07015	E001	3.19E-02	0.13	MFS transporter, putative (*T. verrucosum*)
TERG_07016	E007	6.44E-03	0.07	mitochondrial-processing peptidase subunit beta
TERG_07038	E001	1.20E-02	0.13	glutamine-tRNA ligase
TERG_07127	E003	1.66E-04	-0.07	homocitrate synthase, mitochondrial
TERG_07127	E008	4.97E-02	0.05	homocitrate synthase, mitochondrial
TERG_07143	E001	1.66E-04	-0.47	potassium/sodium efflux P-type ATPase, fungal-type
TERG_07159	E008	7.25E-04	-0.18	geranylgeranyl transferae type II alpha subunit, putative (*T. verrucosum*)
TERG_07187	E005	7.25E-04	-0.05	L-aminoadipate-semialdehyde dehydrogenase large subunit
TERG_07208	E005	8.07E-04	0.07	SNF7 family protein (*T. equinum*)
TERG_07276	E004	2.27E-04	0.72	oxoglutarate dehydrogenase (succinyl-transferring), E1 component
TERG_07414	E001	4.64E-02	-0.53	hypothetical protein
TERG_07461	E003	4.79E-02	0.20	AP-2 complex subunit sigma
TERG_07508	E001	4.98E-03	0.19	hypothetical protein
TERG_07588	E006	1.27E-03	-0.71	5-methyltetrahydropteroyltriglutamate-homocysteine S-methyltransferase
TERG_07630	E003	4.34E-04	0.79	FMN dependent dehydrogenase, putative (*T. verrucosum*)
TERG_07659	E003	2.38E-04	-0.16	peroxisomal 3-ketoacyl-coA thiolase (Kat1), putative (*A. benhamiae*)
TERG_07659	E005	2.01E-03	0.11	peroxisomal 3-ketoacyl-coA thiolase (Kat1), putative (*A. benhamiae*)
TERG_07662	E001	4.40E-02	0.17	mannosylphosphate transferase (*T. tonsurans*)
TERG_07684	E001	2.91E-02	-0.05	hypothetical protein
TERG_07684	E002	2.95E-02	0.06	hypothetical protein
TERG_07821	E001	7.31E-03	-0.29	hypothetical protein

TERG_07821	E002	6.28E-03	0.18	hypothetical protein
TERG_07862	E001	8.42E-03	0.21	dynein light intermediate chain (*T. tonsurans*)
TERG_07942	E005	1.62E-02	0.17	calcium channel subunit Cch1 (*T. tonsurans*)
TERG_07942	E006	4.33E-02	-0.22	calcium channel subunit Cch1 (*T. tonsurans*)
TERG_07966	E007	9.28E-17	-2.02	tryptophan-tRNA ligase
TERG_07966	E009	1.27E-02	0.12	tryptophan-tRNA ligase
TERG_08072	E007	3.62E-02	0.17	C6 transcription factor, putative (*A. benhamiae*)
TERG_08091	E004	1.02E-02	0.09	conidial pigment biosynthesis oxidase Abr1/brown (*T. verrucosum*)
TERG_08145	E001	2.76E-02	0.12	ADP-ribose 1"-phosphate phosphatase (*T. equinum*)
TERG_08250	E001	3.41E-04	-0.41	cation diffusion facilitator family metal ion transporter, putative (*T. verrucosum*)
TERG_08258	E005	1.95E-02	-0.47	hypothetical protein
TERG_08286	E002	9.88E-03	-0.02	GPI anchored CFEM domain protein (*T. verrucosum*)
TERG_08286	E003	2.14E-02	0.03	GPI anchored CFEM domain protein (*T. verrucosum*)
TERG_08312	E002	1.09E-03	0.04	mitochondrial heat shock protein Hsp10 (*A. benhamiae*)
TERG_08312	E003	2.35E-02	-0.06	mitochondrial heat shock protein Hsp10 (*A. benhamiae*)
TERG_08353	E002	6.32E-05	-1.00	cytochrome P450 55A3 (*T. tonsurans*)
TERG_08353	E011	1.07E-02	-0.95	cytochrome P450 55A3 (*T. tonsurans*)
TERG_08426	E001	3.76E-03	0.07	copper-translocating P-type ATPase
TERG_08462	E011	4.47E-02	-0.31	NAD kinase/ATP NAD kinase (*T. tonsurans*)
TERG_08491	E001	2.27E-05	0.33	Elongation factor Tu (*T. equinum*)
TERG_08491	E006	1.56E-05	-0.21	Elongation factor Tu (*T. equinum*)
TERG_08648	E003	9.96E-03	-0.44	cell division control protein (Cdc15), putative (*A. benhamiae*)
TERG_08648	E004	1.90E-04	0.12	cell division control protein (Cdc15), putative (*A. benhamiae*)
TERG_08751	E002	1.35E-02	0.13	ABC multidrug transporter, putative (*A. benhamiae*)
TERG_08980	E001	1.93E-04	1.91	hypothetical protein
TERG_08980	E002	2.14E-02	1.98	hypothetical protein
TERG_08982	E001	1.89E-04	0.23	hypothetical protein
TERG_08982	E002	1.73E-04	-0.15	hypothetical protein
TERG_08991	E004	6.16E-04	0.06	ATPase 2 nuclear control (*M. gypseum*)
TERG_11660	E002	7.62E-03	0.09	mitochondrial 40S ribosomal protein (*T. tonsurans*)
TERG_11660	E003	9.40E-06	0.24	mitochondrial 40S ribosomal protein (*T. tonsurans*)
TERG_11660	E004	4.26E-09	-1.13	mitochondrial 40S ribosomal protein (*T. tonsurans*)
TERG_11660	E005	2.08E-06	-0.79	mitochondrial 40S ribosomal protein (*T. tonsurans*)
TERG_11660	E006	2.56E-09	-0.47	mitochondrial 40S ribosomal protein (*T. tonsurans*)
TERG_11660	E007	5.39E-03	-0.37	mitochondrial 40S ribosomal protein (*T. tonsurans*)
TERG_11688	E001	5.08E-10	-0.35	nitrilase (*T. tonsurans*)
TERG_11751	E001	5.31E-07	-0.20	PCI domain-containing protein (*T. tonsurans*)
TERG_11751	E002	3.87E-08	-0.15	PCI domain-containing protein (*T. tonsurans*)
TERG_11751	E005	6.96E-06	0.06	PCI domain-containing protein (*T. tonsurans*)
TERG_11871	E002	4.33E-02	0.20	ubiquitin C-terminal hydrolase 37 (*T. verrucosum*)

TERG_11879	E001	3.31E-02	-0.23	hypothetical protein
TERG_11879	E002	2.86E-02	0.05	hypothetical protein
TERG_11991	E003	4.90E-02	0.62	hypothetical protein
TERG_12154	E016	7.92E-06	-0.09	leukotriene A4 hydrolase (*T. verrucosum*)
TERG_12303	E001	1.66E-02	0.35	myosin-1
TERG_12303	E002	2.83E-02	0.21	myosin-1
TERG_12642	E004	3.87E-02	0.19	Poly(A) polymerase PAPalpha (*T. tonsurans*)

Total genes = 214, Differential exon usage = 309.

Supplementary Table S4. Read counts in intronic regions obtained from RNA-seq data analysis using Perl script.

Transcript	Intron	Gene Product Name	0 hour	3 hours	12 hours
TERG_00014T0	Intron1	hypothetical protein	1069	0	8
TERG_00017T0	Intron2	Smr domain protein (*T. verrucosum*)	4490	10660	501
TERG_00024T0	Intron1	ADP-ribosylation factor 6	0	0	19
TERG_00024T0	Intron3	ADP-ribosylation factor 6	0	0	32
TERG_00024T1	Intron1	ADP-ribosylation factor 6	0	0	22
TERG_00029T0	Intron1	copper fist DNA binding domain-containing protein (*T. equinum*)	0	0	75
TERG_00057T0	Intron1	hypothetical protein	0	0	130
TERG_00089T0	Intron1	methylthioadenosine phosphorylase	30	0	776
TERG_00089T0	Intron2	methylthioadenosine phosphorylase	0	0	137
TERG_00090T0	Intron2	cellular morphogenesis protein (Bud22), putative (*A. benhamiae*)	0	0	36
TERG_00105T0	Intron1	40S ribosomal protein S30	0	0	68
TERG_00106T0	Intron1	60S ribosomal protein L24	0	0	13
TERG_00106T0	Intron2	60S ribosomal protein L24	0	0	259
TERG_00124T0	Intron1	peroxisomal matrix protein (*T. equinum*)	0	0	1340
TERG_00130T0	Intron1	Rab geranylgeranyl transferase escort protein, putative (*T. verrucosum*)	0	0	136
TERG_00139T0	Intron1	MADS box transcription factor (*T. tonsurans*)	0	0	212
TERG_00141T0	Intron2	DUF803 domain-containing protein (*T. equinum*)	0	0	17
TERG_00163T0	Intron6	siderochrome-iron transporter, putative (*A. benhamiae*)	0	0	478
TERG_00170T0	Intron1	hypothetical protein	0	0	17
TERG_00170T1	Intron1	hypothetical protein	0	0	24
TERG_00184T0	Intron2	ribose-phosphate pyrophosphokinase (*T. tonsurans*)	0	0	3109
TERG_00206T0	Intron1	DNA repair protein rad9 (*T. equinum*)	0	0	214
TERG_00221T0	Intron1	hypothetical protein	0	0	41
TERG_00221T0	Intron2	hypothetical protein	0	0	10
TERG_00222T0	Intron1	CP2 transcription factor, putative (*A. benhamiae*)	0	0	143
TERG_00222T0	Intron2	CP2 transcription factor, putative (*A. benhamiae*)	0	0	611
TERG_00231T0	Intron1	glutamate decarboxylase (*T. equinum*)	0	0	288
TERG_00241T1	Intron2	hypothetical protein	0	1217	78
TERG_00250T0	Intron1	phosphoacetylglucosamine mutase (*T. equinum*)	0	0	290
TERG_00266T0	Intron1	hypothetical protein	5524	0	177
TERG_00273T0	Intron1	mucin (*M. canis*)	61473	192701	167539
TERG_00276T4	Intron1	TATA-box-binding protein	8828	0	1576
TERG_00276T4	Intron2	TATA-box-binding protein	9275	4795	206
TERG_00276T4	Intron3	TATA-box-binding protein	0	3116	0
TERG_00276T4	Intron4	TATA-box-binding protein	1275	0	0
TERG_00276T3	Intron1	TATA-box-binding protein	0	0	519
TERG_00276T3	Intron2	TATA-box-binding protein	6453	9180	301
TERG_00276T3	Intron3	TATA-box-binding protein	0	3613	64
TERG_00276T3	Intron4	TATA-box-binding protein	381	0	0
TERG_00276T2	Intron1	TATA-box-binding protein	0	0	716

TERG_00276T2	Intron2	TATA-box-binding protein	0	1817	50
TERG_00276T2	Intron3	TATA-box-binding protein	1173	0	0
TERG_00276T1	Intron1	TATA-box-binding protein	0	0	639
TERG_00276T1	Intron2	TATA-box-binding protein	7306	8114	272
TERG_00276T1	Intron3	TATA-box-binding protein	0	2612	39
TERG_00276T1	Intron4	TATA-box-binding protein	529	0	24
TERG_00276T0	Intron1	TATA-box-binding protein	0	0	531
TERG_00276T0	Intron2	TATA-box-binding protein	0	1082	57
TERG_00276T0	Intron3	TATA-box-binding protein	1290	0	62
TERG_00288T0	Intron1	5'/3'-nucleotidase SurE	4559	0	0
TERG_00288T0	Intron3	5'/3'-nucleotidase SurE	0	0	13370
TERG_00302T1	Intron1	GTP-binding protein rho2	0	0	97
TERG_00302T1	Intron2	GTP-binding protein rho2	0	0	92
TERG_00302T0	Intron1	GTP-binding protein rho2	0	0	162
TERG_00302T2	Intron1	GTP-binding protein rho2	0	0	62
TERG_00302T2	Intron2	GTP-binding protein rho2	0	0	38
TERG_00328T0	Intron1	hypothetical protein	0	0	170
TERG_00332T0	Intron1	nuclear transport factor 2	0	0	115
TERG_00332T0	Intron2	nuclear transport factor 2	7908	0	0
TERG_00332T0	Intron4	nuclear transport factor 2	0	0	617
TERG_00332T0	Intron5	nuclear transport factor 2	0	0	45
TERG_00342T0	Intron1	phosphoglucomutase	0	5690	7
TERG_00369T0	Intron3	branchpoint-bridging protein	0	0	290
TERG_00373T0	Intron1	hypothetical protein	931	0	54
TERG_00383T0	Intron1	60S ribosomal protein L16	0	0	12
TERG_00383T0	Intron2	60S ribosomal protein L16	2762	19278	67
TERG_00393T0	Intron1	glutenin (*M. canis*)	9946	0	37
TERG_00410T0	Intron1	hypothetical protein	0	0	38
TERG_00415T1	Intron1	KH domain RNA-binding protein (*T. tonsurans*)	0	0	11
TERG_00441T0	Intron1	peptidyl-prolyl cis-trans isomerase pin4	0	0	29
TERG_00444T0	Intron1	3-oxo-5-alpha-steroid 4-dehydrogenase (*T. tonsurans*)	0	0	117
TERG_00453T0	Intron1	PXA domain-containing protein (*T. tonsurans*)	6891	0	3405
TERG_00462T0	Intron1	40S ribosomal protein	3834	0	28
TERG_00466T0	Intron1	SirB protein (*T. equinum*)	0	0	55
TERG_00479T0	Intron1	hypothetical protein	3622	0	69
TERG_00520T0	Intron1	hypothetical protein	0	0	46
TERG_00520T0	Intron3	hypothetical protein	0	0	399
TERG_00523T0	Intron1	hypothetical protein	0	0	27
TERG_00526T0	Intron1	vacuolar protein sorting-associated protein 21 (*T. tonsurans*)	0	0	21
TERG_00529T0	Intron5	acetate kinase	5336	9864	0
TERG_00534T0	Intron1	Arp2/3 complex subunit Arc16 (*T. tonsurans*)	10109	8473	304
TERG_00536T0	Intron3	D-galacturonic acid reductase (*T. equinum*)	1242	0	52
TERG_00536T0	Intron4	D-galacturonic acid reductase (*T. equinum*)	1640	0	750
TERG_00536T1	Intron2	D-galacturonic acid reductase (*T. equinum*)	0	0	10
TERG_00536T1	Intron3	D-galacturonic acid reductase (*T. equinum*)	1035	0	63
TERG_00548T0	Intron3	elongation factor 1-alpha	5094	7015	80

TERG_00566T0	Intron1	hypothetical protein	19292	36399	91
TERG_00570T0	Intron1	autophagy protein (*T. equinum*)	0	0	211
TERG_00574T0	Intron1	MFS multidrug transporter, putative (*T. verrucosum*)	0	0	398
TERG_00576T0	Intron1	saccharopine dehydrogenase (*T. tonsurans*)	0	0	11
TERG_00576T2	Intron1	saccharopine dehydrogenase (*T. tonsurans*)	0	0	17
TERG_00587T0	Intron1	nuclear pore complex protein sonA (*T. equinum*)	0	0	16
TERG_00590T0	Intron1	hypothetical protein	0	1828	7
TERG_00601T0	Intron2	CMGC/DYRK/DYRK2 protein kinase	0	0	112
TERG_00603T0	Intron1	cell wall integrity and stress response component 4 (*M. canis*)	0	0	21
TERG_00607T0	Intron3	mitochondrial carnitine/acylcarnitine carrier protein (*T. tonsurans*)	0	0	254
TERG_00621T0	Intron2	FAD dependent oxidoreductase (*T. tonsurans*)	0	0	19
TERG_00622T0	Intron1	yebR protein (*M. gypseum*)	3631	0	0
TERG_00626T0	Intron1	ADP-ribosylation factor-like protein 1	0	0	30
TERG_00626T0	Intron2	ADP-ribosylation factor-like protein 1	1264	118689	106
TERG_00635T0	Intron2	40S ribosomal protein S14	0	815	67
TERG_00641T0	Intron1	iron-sulfur cluster assembly accessory protein I (*T. equinum*)	89381	509250	415559
TERG_00655T0	Intron1	mitochondrial import inner membrane translocase subunit tim16	56896	153444	187
TERG_00677T0	Intron1	hypothetical protein	0	249	4515
TERG_00696T0	Intron1	WEE/WEE-UNCLASSIFIED protein kinase	0	0	15
TERG_00714T0	Intron1	cell pattern formation-associated protein (*T. tonsurans*)	0	0	37
TERG_00734T0	Intron2	dipeptidase (*T. tonsurans*)	0	0	47
TERG_00738T0	Intron1	60S ribosomal protein L44	986	0	758
TERG_00738T1	Intron1	60S ribosomal protein L44	1887	0	1453
TERG_00744T0	Intron1	CMGC/CDK protein kinase	0	0	203
TERG_00744T0	Intron3	CMGC/CDK protein kinase	5386	0	35
TERG_00757T0	Intron1	hypothetical protein	0	0	135
TERG_00765T0	Intron2	hypothetical protein	0	0	50
TERG_00783T0	Intron3	guanine nucleotide-binding protein subunit beta-like protein	0	0	8225
TERG_00785T0	Intron1	endoplasmic reticulum vesicle protein 25	0	0	34
TERG_00787T0	Intron1	endoplasmic reticulum vesicle protein 25	0	0	42
TERG_00799T0	Intron1	Haloacid dehalogenase (*T. tonsurans*)	0	0	16
TERG_00799T0	Intron3	Haloacid dehalogenase (*T. tonsurans*)	0	0	36
TERG_00818T0	Intron1	aldehyde dehydrogenase 3B1 (*T. equinum*)	0	0	392
TERG_00820T3	Intron1	MFS multidrug transporter, putative (*A. benhamiae*)	0	0	26
TERG_00820T2	Intron1	MFS multidrug transporter, putative (*A. benhamiae*)	74956	261441	4724
TERG_00820T1	Intron2	MFS multidrug transporter, putative (*A. benhamiae*)	0	0	89
TERG_00838T0	Intron1	pH-response transcription factor pacC/RIM101	0	0	48
TERG_00851T0	Intron2	ankyrin repeat-containing protein (*T. equinum*)	0	0	44
TERG_00861T0	Intron1	hypothetical protein	0	2663	83
TERG_00877T0	Intron2	CBS domain-containing protein (*T. equinum*)	93775	14069	47482
TERG_00883T0	Intron5	acyl carrier protein	0	0	108

TERG_00895T0	Intron2	hypothetical protein	0	0	78
TERG_00906T0	Intron1	cAMP-dependent protein kinase regulatory subunit	1564	3173	13306
TERG_00928T0	Intron1	hypothetical protein	0	0	104
TERG_00974T0	Intron1	2,3-bisphosphoglycerate-independent phosphoglycerate mutase	0	0	258
TERG_00984T0	Intron1	PDCD2-C domain-containing protein (*T. equinum*)	0	0	219
TERG_01031T0	Intron1	F-box domain protein (*T. verrucosum*)	0	0	139
TERG_01050T0	Intron1	metal homeostatis protein bsd2 (*T. tonsurans*)	7961	2887	0
TERG_01091T0	Intron2	magnesium-dependent phosphatase-1	0	0	5864
TERG_01109T0	Intron1	WD repeat-containing protein (*T. equinum*)	0	0	54
TERG_01111T0	Intron2	50S ribosomal protein Mrp49 (*T. tonsurans*)	0	0	39
TERG_01148T0	Intron1	hypothetical protein	0	0	81
TERG_01148T0	Intron5	hypothetical protein	0	0	136
TERG_01183T0	Intron1	RNase3 domain-containing protein (*T. equinum*)	0	0	214
TERG_01198T0	Intron1	pre-mRNA-splicing factor rse1	856	0	112
TERG_01198T0	Intron2	pre-mRNA-splicing factor rse1	0	0	1935
TERG_01198T1	Intron2	pre-mRNA-splicing factor rse1	0	0	1650
TERG_01216T0	Intron1	peptidyl-tRNA hydrolase domain protein (*T. verrucosum*)	0	0	11
TERG_01267T1	Intron1	amidophosphoribosyltransferase (*T. equinum*)	0	3263	774
TERG_01267T0	Intron1	amidophosphoribosyltransferase (*T. equinum*)	0	3294	599
TERG_01282T0	Intron1	hypothetical protein	0	0	137
TERG_01284T0	Intron2	UMTA (*T. equinum*)	3714	0	73
TERG_01305T3	Intron1	2-dehydropantoate 2-reductase (*T. verrucosum*)	6452	0	1600
TERG_01305T0	Intron1	2-dehydropantoate 2-reductase (*T. verrucosum*)	2155	0	862
TERG_01305T1	Intron1	2-dehydropantoate 2-reductase (*T. verrucosum*)	3744	196	1427
TERG_01308T0	Intron1	ATP-dependent RNA helicase SUB2	0	0	296
TERG_01321T0	Intron1	BTB/POZ domain-containing protein (*T. tonsurans*)	0	0	29
TERG_01336T0	Intron1	MFS transporter (*T. equinum*)	0	0	13
TERG_01356T0	Intron2	small G-protein GPA2 (*T. tonsurans*)	0	0	70
TERG_01356T0	Intron3	small G-protein GPA2 (*T. tonsurans*)	0	0	571
TERG_01359T0	Intron1	transketolase	4985	0	18
TERG_01359T3	Intron3	transketolase	0	0	35
TERG_01359T1	Intron1	transketolase	9801	0	7
TERG_01363T0	Intron1	SET domain-containing protein (*T. equinum*)	0	0	194
TERG_01369T0	Intron1	ubiquinol-cytochrome C reductase complex subunit UcrQ (*T. tonsurans*)	0	0	65
TERG_01369T0	Intron2	ubiquinol-cytochrome C reductase complex subunit UcrQ (*T. tonsurans*)	0	0	193
TERG_01384T0	Intron1	C6 finger domain-containing protein (*T. equinum*)	0	0	52
TERG_01443T2	Intron4	ABC multidrug transporter (*T. tonsurans*)	0	0	67
TERG_01443T0	Intron5	ABC multidrug transporter (*T. tonsurans*)	0	0	55
TERG_01466T1	Intron1	C6 transcription factor, putative (*T. verrucosum*)	0	6288	0
TERG_01467T0	Intron1	cytochrome P450 monooxygenase, putative (*A. benhamiae*)	0	0	73

TERG_01477T0	Intron1	cwl1 (*M. gypseum*)	0	0	335
TERG_01550T0	Intron1	60S ribosomal protein L38 (*T. tonsurans*)	0	0	79
TERG_01560T0	Intron1	conserved eukaryotic protein (*A. benhamiae*)	4325	0	0
TERG_01564T1	Intron1	pyruvate carboxylase	4969	0	114
TERG_01564T0	Intron1	pyruvate carboxylase	6938	0	76
TERG_01599T1	Intron1	hypothetical protein	1906	0	375
TERG_01604T0	Intron2	60S ribosomal protein	0	0	901
TERG_01633T0	Intron1	CFEM domain protein, putative (*A. benhamiae*)	0	0	32
TERG_01633T0	Intron2	CFEM domain protein, putative (*A. benhamiae*)	941	0	150
TERG_01660T0	Intron2	hypothetical protein	2782	0	12263
TERG_01670T2	Intron1	isocitrate dehydrogenase [NAD] subunit 1, mitochondrial	0	0	45
TERG_01670T0	Intron3	isocitrate dehydrogenase [NAD] subunit 1, mitochondrial	0	0	97
TERG_01683T0	Intron2	hypothetical protein	0	0	45
TERG_01694T0	Intron2	ankyrin repeat-containing protein (*T. equinum*)	0	0	147
TERG_01707T0	Intron1	hypothetical protein	0	0	265
TERG_01800T1	Intron1	isopentenyl-diphosphate delta-isomerase	0	0	13
TERG_01800T2	Intron1	isopentenyl-diphosphate delta-isomerase	0	0	59
TERG_01877T1	Intron3	FAM96B (*M. canis*)	11105	21370	1600
TERG_01883T0	Intron1	hsp75-like protein	3390	0	15
TERG_01884T0	Intron1	hypothetical protein	16581	0	30
TERG_01889T0	Intron1	RING finger domain-containing protein (*T. tonsurans*)	0	0	1313
TERG_01908T0	Intron2	hypothetical protein	0	0	61
TERG_01950T0	Intron1	serine/threonine protein kinase (*T. tonsurans*)	0	0	490
TERG_01952T0	Intron2	40S ribosomal protein S15	0	0	33
TERG_01959T0	Intron1	RNA polymerase Rpb1 C-terminal repeat domain-containing protein (*T. equinum*)	0	0	1675
TERG_01964T0	Intron3	cystathionine gamma-lyase (*T. equinum*)	0	0	48
TERG_01975T0	Intron1	hypothetical protein	0	0	89
TERG_02012T0	Intron1	adenosine deaminase family protein (*A. benhamiae*)	0	0	28
TERG_02031T0	Intron1	methylenetetrahydrofolate dehydrogenase (*T. equinum*)	0	0	105
TERG_02032T2	Intron1	ATP synthase subunit alpha, mitochondrial	1897	0	0
TERG_02032T1	Intron1	ATP synthase subunit alpha, mitochondrial	1470	0	113
TERG_02032T0	Intron1	ATP synthase subunit alpha, mitochondrial	1761	0	12
TERG_02071T0	Intron1	phosphoinositide-specific phospholipase C (*T. equinum*)	10159	0	159
TERG_02071T0	Intron2	phosphoinositide-specific phospholipase C (*T. equinum*)	0	0	3755
TERG_02071T0	Intron3	phosphoinositide-specific phospholipase C (*T. equinum*)	3084	0	820
TERG_02078T0	Intron1	thiamine biosynthesis protein Nmt1 (*T. equinum*)	0	0	213
TERG_02104T0	Intron2	translation machinery-associated protein 22	3401	0	76
TERG_02118T1	Intron3	GPI anchored protein, putative (*A. benhamiae*)	6918	0	176
TERG_02127T0	Intron1	sybindin-like family protein (*T. tonsurans*)	567810	204318	1739
TERG_02127T0	Intron2	sybindin-like family protein (*T. tonsurans*)	10645	0	0
TERG_02140T0	Intron1	bZIP transcription factor (*T. tonsurans*)	32226	21371	2540

TERG_02140T0	Intron3	bZIP transcription factor (*T. tonsurans*)	0	0	65
TERG_02179T0	Intron1	keylime pathogenicity protein (*M. canis*)	5317	0	0
TERG_02180T0	Intron1	hypothetical protein	39343	12783	30
TERG_02237T0	Intron1	40S ribosomal protein S28	0	0	36
TERG_02250T0	Intron1	cyclopropane-fatty-acyl-phospholipid synthase (*T. tonsurans*)	4392	0	0
TERG_02250T1	Intron1	cyclopropane-fatty-acyl-phospholipid synthase (*T. tonsurans*)	2572	0	0
TERG_02253T0	Intron1	protein required for cell viability Rrp17, putative (*A. benhamiae*)	1435	0	0
TERG_02262T0	Intron2	NACHT and WD domain protein (*T. verrucosum*)	0	0	234
TERG_02262T0	Intron4	NACHT and WD domain protein (*T. verrucosum*)	0	0	206
TERG_02264T0	Intron1	hypothetical protein	1453	0	18
TERG_02269T0	Intron1	hypothetical protein	4534	0	97
TERG_02275T0	Intron1	hypothetical protein	0	0	16
TERG_02287T0	Intron4	calcium dependent mitochondrial carrier protein (*T. tonsurans*)	49879	12364	891
TERG_02312T0	Intron1	sister chromatid cohesion protein Dcc1, putative (*A. benhamiae*)	6353	0	0
TERG_02376T1	Intron1	60S ribosomal protein (*T. equinum*)	0	0	45
TERG_02376T0	Intron3	60S ribosomal protein (*T. equinum*)	0	0	16
TERG_02399T0	Intron1	RNA-directed RNA polymerase (*T. tonsurans*)	0	0	38
TERG_02405T0	Intron1	mating locus protein, putative (*T. verrucosum*)	0	0	1600
TERG_02413T0	Intron1	hypothetical protein	0	0	180
TERG_02428T0	Intron1	COX1 assembly protein (*T. tonsurans*)	0	0	896
TERG_02438T1	Intron1	hypothetical protein	7363	3554	0
TERG_02438T0	Intron1	hypothetical protein	3216	3888	0
TERG_02441T0	Intron1	LYR family protein (*M. canis*)	0	0	22
TERG_02460T0	Intron1	hypothetical protein	18158	10926	1375
TERG_02488T0	Intron1	hypothetical protein	0	3095	475
TERG_02494T1	Intron1	nucleolar essential protein 1 (*T. equinum*)	3776	995	0
TERG_02494T0	Intron1	nucleolar essential protein 1 (*T. equinum*)	3585	1540	0
TERG_02519T0	Intron1	chitin synthase activator (*T. equinum*)	31447	18312	43
TERG_02524T0	Intron1	proline-specific permease (*T. equinum*)	12938	2103	7
TERG_02550T0	Intron1	ZZ type zinc finger domain protein (*T. verrucosum*)	421083	1422177	2864
TERG_02551T1	Intron1	extensin (*M. canis*)	7374	19960	150
TERG_02551T0	Intron1	extensin (*M. canis*)	5354	26418	376
TERG_02591T0	Intron1	D-tyrosyl-tRNA(Tyr) deacylase	0	0	152
TERG_02605T0	Intron2	CCCH zinc finger domain-containing protein (*T. equinum*)	0	0	1841
TERG_02624T0	Intron1	hypothetical protein	0	0	14
TERG_02634T0	Intron1	C2H2 finger domain protein, putative (*A. benhamiae*)	0	0	143
TERG_02637T2	Intron1	RING-14 protein (*T. equinum*)	4311	0	1070
TERG_02637T1	Intron1	RING-14 protein (*T. equinum*)	0	0	64
TERG_02637T0	Intron1	RING-14 protein (*T. equinum*)	0	0	85
TERG_02669T0	Intron2	pre-mRNA splicing factor (*T. equinum*)	0	0	30
TERG_02670T0	Intron2	pre-mRNA splicing factor (Prp24), putative (*T. verrucosum*)	0	0	104

TERG_02670T0	Intron4	pre-mRNA splicing factor (Prp24), putative (*T. verrucosum*)	0	0	12
TERG_02694T3	Intron1	sulfite efflux pump SSU1	0	0	226
TERG_02694T1	Intron1	sulfite efflux pump SSU1	0	0	120
TERG_02694T0	Intron1	sulfite efflux pump SSU1	0	0	263
TERG_02694T2	Intron1	sulfite efflux pump SSU1	0	0	198
TERG_02697T0	Intron4	G-patch domain-containing protein (*T. tonsurans*)	4939	0	0
TERG_02709T0	Intron2	syntaxin 5 (*T. tonsurans*)	0	0	26
TERG_02760T0	Intron1	hypothetical protein	0	0	37
TERG_02761T0	Intron1	BUB protein kinase	0	0	180
TERG_02785T0	Intron1	hypothetical protein	0	0	177
TERG_02786T0	Intron2	ADP-ribosylation factor (*T. tonsurans*)	261	0	20
TERG_02786T0	Intron3	ADP-ribosylation factor (*T. tonsurans*)	5824	0	94
TERG_02897T0	Intron3	methylene-fatty-acyl-phospholipid synthase (*T. equinum*)	0	0	23
TERG_02906T0	Intron1	PAB1 binding protein (*T. tonsurans*)	0	0	42
TERG_02937T0	Intron1	importin beta-4 subunit (*T. tonsurans*)	0	0	4543
TERG_02937T1	Intron2	importin beta-4 subunit (*T. tonsurans*)	125	0	4153
TERG_02960T0	Intron6	Beta-ketoacyl synthase (*T. tonsurans*)	0	0	55
TERG_02977T0	Intron1	hypothetical protein	6730	0	11
TERG_02977T0	Intron2	hypothetical protein	0	5176	0
TERG_03014T0	Intron2	ER membrane protein Wsc4 (*T. equinum*)	0	0	781
TERG_03038T0	Intron2	translation elongation factor Tu	4944	0	41
TERG_03039T0	Intron1	Cytochrome b5 (*T. tonsurans*)	4874	0	0
TERG_03042T0	Intron1	STE/STE20/PAKA protein kinase	0	0	56
TERG_03050T0	Intron1	tyrosine-tRNA ligase	0	0	54
TERG_03058T0	Intron1	zinc-binding oxidoreductase ToxD (*T. tonsurans*)	0	0	269
TERG_03078T0	Intron1	cytochrome P450 oxidoreductase OrdA-like, putative (*T. verrucosum*)	0	27548	169
TERG_03085T0	Intron4	ATP-dependent RNA helicase DBP2	0	0	848
TERG_03087T0	Intron3	C2H2 transcription factor (Con7), putative (*A. benhamiae*)	1269	4683	228
TERG_03097T0	Intron2	hypothetical protein	0	0	409
TERG_03151T0	Intron1	import inner membrane translocase subunit tim-21 (*T. equinum*)	0	0	11
TERG_03163T6	Intron1	eukaryotic translation initiation factor 4 gamma (*M. gypseum*)	3562	3066	235
TERG_03163T5	Intron2	eukaryotic translation initiation factor 4 gamma (*M. gypseum*)	4479	6333	142
TERG_03177T0	Intron1	RING finger domain protein, putative (*A. benhamiae*)	1020	0	48
TERG_03187T0	Intron1	aspartate aminotransferase (*T. tonsurans*)	0	0	49
TERG_03190T0	Intron1	NADPH oxidase regulator NoxR (*T. equinum*)	263	0	0
TERG_03196T0	Intron1	universal stress protein family domain protein (*A. benhamiae*)	0	0	1581
TERG_03206T0	Intron1	hsp7-like protein	2748	0	42
TERG_03240T0	Intron1	transmembrane efflux protein (*T. tonsurans*)	4064	0	0
TERG_03302T0	Intron1	hypothetical protein	0	0	395
TERG_03319T1	Intron2	small nuclear ribonucleoprotein Sm D1 (*T. tonsurans*)	0	0	57
TERG_03343T0	Intron1	fatty acid desaturase (*T. equinum*)	0	0	104

TERG_03459T0	Intron1	GDSL Lipase/Acylhydrolase family protein (*A. benhamiae*)	40167	0	979
TERG_03463T0	Intron3	hypothetical protein	0	0	310
TERG_03473T0	Intron1	hypothetical protein	2218	0	0
TERG_03497T0	Intron2	hemolysin-III family protein (*T. equinum*)	1102	0	44
TERG_03500T0	Intron1	urease accessory protein UreG	143	0	47
TERG_03517T1	Intron1	cytochrome c oxidase subunit Vib (*T. tonsurans*)	13510	35	0
TERG_03517T0	Intron2	cytochrome c oxidase subunit Vib (*T. tonsurans*)	2934	0	0
TERG_03519T0	Intron1	peptidyl-prolyl cis-trans isomerase (*T. tonsurans*)	5574	6648	481
TERG_03528T2	Intron1	cytochrome c	0	0	133
TERG_03528T4	Intron1	cytochrome c	0	0	133
TERG_03528T5	Intron1	cytochrome c	0	0	121
TERG_03528T3	Intron1	cytochrome c	0	0	109
TERG_03531T0	Intron1	hypothetical protein	0	0	16
TERG_03556T0	Intron1	transcriptional regulator Medusa (*A. benhamiae*)	14981	46258	27698
TERG_03559T0	Intron1	alcohol dehydrogenase (*T. tonsurans*)	0	0	714
TERG_03569T0	Intron1	RING finger domain-containing protein (*T. equinum*)	0	0	113
TERG_03579T0	Intron1	MFS transporter (*T. equinum*)	305	0	0
TERG_03579T1	Intron1	MFS transporter (*T. equinum*)	1390	0	0
TERG_03579T3	Intron1	MFS transporter (*T. equinum*)	1367	0	17
TERG_03579T4	Intron1	MFS transporter (*T. equinum*)	0	0	21
TERG_03631T0	Intron1	sister chromatid cohesion factor (*T. equinum*)	0	0	23
TERG_03653T0	Intron1	hypothetical protein	3356	94	658
TERG_03670T0	Intron1	hypothetical protein	0	0	112
TERG_03695T0	Intron1	pyrroline-5-carboxylate reductase	0	0	12
TERG_03706T0	Intron1	trichodiene oxygenase (*T. equinum*)	0	0	70
TERG_03706T0	Intron5	trichodiene oxygenase (*T. equinum*)	0	0	37
TERG_03706T0	Intron8	trichodiene oxygenase (*T. equinum*)	2354	0	40
TERG_03735T0	Intron1	SAGA complex component Sgf29 (*T. tonsurans*)	5486	0	29
TERG_03736T0	Intron6	transcriptional corepressor Cyc8 (*T. equinum*)	0	0	521
TERG_03783T0	Intron1	siroheme synthase Met8 (*T. tonsurans*)	0	0	299
TERG_03827T0	Intron1	hypothetical protein	0	0	96
TERG_03835T0	Intron2	hypothetical protein	0	0	136
TERG_03843T3	Intron1	chitin synthase B	2023	0	83
TERG_03843T2	Intron1	chitin synthase B	3005	0	142
TERG_03843T1	Intron1	chitin synthase B	1054	0	209
TERG_03843T0	Intron1	chitin synthase B	2274	0	0
TERG_03843T5	Intron1	chitin synthase B	2346	0	158
TERG_03843T4	Intron1	chitin synthase B	2045	0	75
TERG_03845T0	Intron2	hypothetical protein	19190	19489	337
TERG_03872T2	Intron4	CMGC/MAPK/P38 protein kinase	0	0	148
TERG_03880T0	Intron1	60S ribosomal protein L5	11433	0	10
TERG_03896T0	Intron2	mannose-6-phosphate isomerase	0	0	10
TERG_03920T0	Intron1	protein kinase subdomain-containing protein (*M. gypseum*)	0	0	14

TERG_03933T0	Intron5	ABC metal ion transporter (*T. equinum*)	838	0	0
TERG_03933T1	Intron4	ABC metal ion transporter (*T. equinum*)	23	0	0
TERG_03933T2	Intron5	ABC metal ion transporter (*T. equinum*)	236	0	0
TERG_03933T3	Intron5	ABC metal ion transporter (*T. equinum*)	875	0	0
TERG_03933T4	Intron5	ABC metal ion transporter (*T. equinum*)	209	0	0
TERG_03933T5	Intron5	ABC metal ion transporter (*T. equinum*)	225	0	0
TERG_03933T1	Intron4	ABC metal ion transporter (*T. equinum*)	23	0	0
TERG_03933T1	Intron46	ABC metal ion transporter (*T. equinum*)	23	0	0
TERG_03974T0	Intron1	glycine-rich protein (*T. tonsurans*)	0	3644	32
TERG_03995T0	Intron2	hypothetical protein	2697	0	0
TERG_04033T0	Intron1	60S ribosomal protein L2	10615	0	465
TERG_04095T1	Intron1	hypothetical protein	84061	43455	177
TERG_04095T0	Intron1	hypothetical protein	75211	37121	355
TERG_04112T0	Intron1	ADP-ribose pyrophosphatase (*T. equinum*)	0	764	383
TERG_04112T0	Intron2	ADP-ribose pyrophosphatase (*T. equinum*)	0	6918	8481
TERG_04112T0	Intron3	ADP-ribose pyrophosphatase (*T. equinum*)	0	0	356
TERG_04122T0	Intron1	mitochondrial DNA replication protein YHM2	2504	0	0
TERG_04154T0	Intron1	hypothetical protein	0	0	26
TERG_04157T0	Intron1	hypothetical protein	32263	45231	47
TERG_04208T0	Intron1	HAL protein kinase	0	0	217
TERG_04209T0	Intron2	TRAPP complex subunit Bet5 (predicted) (*A. benhamiae*)	24395	818	95
TERG_04229T2	Intron2	AP-1 complex subunit mu-1 (*T. tonsurans*)	0	0	12
TERG_04230T0	Intron1	RNase III domain-containing protein (*T. equinum*)	0	0	48
TERG_04231T1	Intron2	succinate dehydrogenase, cytochrome b556 subunit	0	0	950
TERG_04231T0	Intron1	succinate dehydrogenase, cytochrome b556 subunit	0	0	531
TERG_04234T1	Intron1	hydrophobin, putative (*T. verrucosum*)	0	0	49
TERG_04234T0	Intron1	hydrophobin, putative (*T. verrucosum*)	0	0	65
TERG_04237T0	Intron1	ceramide glucosyltransferase (*T. tonsurans*)	0	476	232
TERG_04269T1	Intron1	hypothetical protein	2064	41244	16174
TERG_04269T0	Intron1	hypothetical protein	3924	40537	16979
TERG_04271T0	Intron1	hypothetical protein	52734	3269	11796
TERG_04277T0	Intron1	epoxide hydrolase (*T. tonsurans*)	1063	0	956
TERG_04285T0	Intron1	transcriptional regulator (Cti6), putative (*T. verrucosum*)	30815	37690	86
TERG_04289T0	Intron1	hypothetical protein	0	0	13
TERG_04297T0	Intron1	ab-hydrolase associated lipase, putative (*A. benhamiae*)	0	0	30
TERG_04309T0	Intron3	multidrug resistance protein (*T. equinum*)	0	0	1092
TERG_04309T0	Intron4	multidrug resistance protein (*T. equinum*)	0	915	249
TERG_04309T0	Intron6	multidrug resistance protein (*T. equinum*)	0	0	31
TERG_04333T0	Intron1	staphylococcal nuclease domain-containing protein 1 (*T. equinum*)	0	0	212
TERG_04336T0	Intron3	transaldolase	0	0	99
TERG_04336T0	Intron6	transaldolase	0	0	45
TERG_04340T0	Intron2	alpha/beta hydrolase, putative (*T. verrucosum*)	8820	0	1411
TERG_04344T0	Intron1	serine/threonine-protein phosphatase 2B catalytic subunit	694	0	0

TERG_04344T1	Intron1	serine/threonine-protein phosphatase 2B catalytic subunit	2278	0	0
TERG_04346T0	Intron2	HSP70 family protein (*A. benhamiae*)	0	0	24
TERG_04346T0	Intron7	HSP70 family protein (*A. benhamiae*)	0	0	18
TERG_04356T0	Intron3	amino acid permease, putative (*A. benhamiae*)	0	0	147
TERG_04390T0	Intron1	NADH-ubiquinone oxidoreductase (*T. tonsurans*)	0	0	23
TERG_04395T0	Intron3	phosphotransferase enzyme family protein (*T. equinum*)	0	0	36
TERG_04399T0	Intron3	MFS transporter, putative (*A. benhamiae*)	0	0	14
TERG_04442T0	Intron3	hypothetical protein	0	10	46333
TERG_04458T0	Intron3	RdgB/HAM1 family non-canonical purine NTP pyrophosphatase	0	0	1843
TERG_04460T3	Intron1	nucleosome assembly protein Nap1 (*T. equinum*)	0	0	54
TERG_04469T0	Intron1	D-lactate dehydrogenase, putative (*T. verrucosum*)	0	0	82
TERG_04472T0	Intron1	hypothetical protein	6364	0	18
TERG_04507T0	Intron1	dual specificity phosphatase (*T. equinum*)	0	18093	292
TERG_04535T0	Intron3	EMP24-GP25L family protein (*T. equinum*)	5756	0	21
TERG_04542T1	Intron1	translation initiation factor eIF4E3 (*T. tonsurans*)	2681	0	27
TERG_04542T2	Intron1	translation initiation factor eIF4E3 (*T. tonsurans*)	1592	0	36
TERG_04542T0	Intron1	translation initiation factor eIF4E3 (*T. tonsurans*)	1111	0	7
TERG_04558T0	Intron2	nucleoside diphosphate kinase	0	0	342
TERG_04575T0	Intron2	transcription initiation factor TFIID subunit 6 (*T. tonsurans*)	0	0	19
TERG_04587T1	Intron2	GPI anchored cell wall protein, putative (*A. benhamiae*)	0	0	23
TERG_04592T0	Intron2	methionine aminopeptidase, type II	0	0	22
TERG_04594T0	Intron1	porin (*T. tonsurans*)	0	0	173
TERG_04595T4	Intron1	asparagine synthase (glutamine-hydrolyzing)	0	0	23
TERG_04601T0	Intron1	hypothetical protein	0	0	46
TERG_04612T0	Intron2	branched-chain amino acid aminotransferase	0	0	739
TERG_04612T1	Intron2	branched-chain amino acid aminotransferase	0	0	529
TERG_04612T2	Intron1	branched-chain amino acid aminotransferase	0	0	777
TERG_04612T3	Intron1	branched-chain amino acid aminotransferase	0	0	472
TERG_04640T0	Intron2	phosphoethanolamine (*T. tonsurans*)	0	0	94
TERG_04644T0	Intron1	mitochondrial carrier protein (*T. tonsurans*)	0	0	45
TERG_04663T0	Intron1	RNA polymerase II holoenzyme/mediator complex component Rgr1, putative (*T. verrucosum*)	3495	0	0
TERG_04677T0	Intron1	guanylate kinase	253	0	0
TERG_04691T0	Intron2	nuclear division Rft1 protein, putative (*T. verrucosum*)	12491	12282	5896
TERG_04697T0	Intron1	iron/copper transporter (*T. tonsurans*)	0	0	897
TERG_04707T0	Intron3	cation transporter ChaC (*T. tonsurans*)	0	0	47
TERG_04707T0	Intron5	cation transporter ChaC (*T. tonsurans*)	126	0	14
TERG_04709T2	Intron1	glucose-6-phosphate 1-dehydrogenase	799	0	59
TERG_04709T4	Intron1	glucose-6-phosphate 1-dehydrogenase	610	0	0
TERG_04713T0	Intron1	cystathionine beta-synthase (*T. tonsurans*)	0	0	35
TERG_04717T0	Intron2	DUF431 domain-containing protein (*T. equinum*)	0	0	60

TERG_04726T0	Intron2	nuclear transcription factor Y subunit B-7 (*T. equinum*)	2446	0	0
TERG_04728T0	Intron1	nuclear transcription factor Y subunit B-7 (*T. equinum*)	3392	0	0
TERG_04733T0	Intron1	CAMK/CAMK1/CAMK1-CMK protein kinase	0	0	19
TERG_04748T0	Intron3	V-type proton ATPase subunit F	0	0	11
TERG_04777T0	Intron1	Exocyst complex component Sec3, putative (*A. benhamiae*)	0	0	17
TERG_04782T0	Intron1	37S ribosomal protein Mrp10 (*T. equinum*)	0	0	69
TERG_04782T1	Intron2	37S ribosomal protein Mrp10 (*T. equinum*)	0	0	28
TERG_04804T1	Intron1	ER-derived vesicles protein ERV14	0	0	42
TERG_04828T0	Intron1	IZH family channel protein (*T. tonsurans*)	0	0	19
TERG_04835T0	Intron2	50S ribosomal protein L4 (*T. tonsurans*)	353	0	7832
TERG_04842T0	Intron1	origin recognition complex subunit Orc4, putative (*T. verrucosum*)	0	0	99
TERG_04851T0	Intron1	Acyl CoA binding protein family protein (*A. benhamiae*)	0	0	113
TERG_04852T0	Intron2	U-box domain-containing protein (*T. tonsurans*)	10424	0	537
TERG_04881T0	Intron1	hypothetical protein	1254	0	11
TERG_04906T0	Intron1	Cytochrome P450 61 (*A. benhamiae*)	0	0	178
TERG_04906T0	Intron2	Cytochrome P450 61 (*A. benhamiae*)	0	0	44
TERG_04946T1	Intron1	rho GTPase ModA (*T. tonsurans*)	0	0	216
TERG_04946T0	Intron1	rho GTPase ModA (*T. tonsurans*)	0	0	181
TERG_04985T0	Intron2	ATP synthase subunit F (*T. tonsurans*)	0	0	43
TERG_04985T0	Intron3	ATP synthase subunit F (*T. tonsurans*)	0	0	62
TERG_04992T2	Intron1	CTLH domain-containing protein (*M. gypseum*)	0	3071	2657
TERG_04992T1	Intron1	CTLH domain-containing protein (*M. gypseum*)	22355	35307	19823093
TERG_04992T1	Intron2	CTLH domain-containing protein (*M. gypseum*)	0	2666	2573
TERG_04992T0	Intron2	CTLH domain-containing protein (*M. gypseum*)	25470	37793	19800588
TERG_04992T0	Intron3	CTLH domain-containing protein (*M. gypseum*)	0	3312	2533
TERG_05046T0	Intron4	hypothetical protein	0	0	46
TERG_05049T0	Intron2	nucleoside diphosphatase (*T. tonsurans*)	0	0	15
TERG_05068T0	Intron1	hypothetical protein	4107	10023	626
TERG_05069T0	Intron1	GMF family protein (*T. equinum*)	193919	129835	562
TERG_05076T0	Intron3	COPI-coated vesicle protein (*T. equinum*)	0	0	83
TERG_05090T3	Intron1	rho-gdp dissociation inhibitor (*T. equinum*)	0	0	79
TERG_05090T0	Intron1	rho-gdp dissociation inhibitor (*T. equinum*)	0	0	139
TERG_05090T1	Intron1	rho-gdp dissociation inhibitor (*T. equinum*)	0	0	134
TERG_05090T5	Intron1	rho-gdp dissociation inhibitor (*T. equinum*)	0	0	365
TERG_05146T0	Intron3	protein methyltransferase (*T. tonsurans*)	0	0	48
TERG_05164T0	Intron1	mucin, putative (*T. verrucosum*)	0	0	45
TERG_05174T0	Intron1	acetyltransferase, GNAT family (*T. verrucosum*)	0	0	634
TERG_05174T1	Intron1	acetyltransferase, GNAT family (*T. verrucosum*)	0	0	567
TERG_05231T0	Intron2	glycerol-3-phosphate phosphatase (*T. tonsurans*)	0	0	15
TERG_05231T0	Intron3	glycerol-3-phosphate phosphatase (*T. tonsurans*)	0	0	142

TERG_05231T1	Intron1	glycerol-3-phosphate phosphatase (*T. tonsurans*)	0	0	103
TERG_05244T0	Intron1	hypothetical protein	5815	4562	50
TERG_05248T0	Intron1	MFS multidrug transporter (*T. tonsurans*)	0	0	30
TERG_05261T0	Intron1	karyopherin alpha subunit (*T. tonsurans*)	0	0	257
TERG_05263T0	Intron1	CCCH zinc finger DNA binding protein (*A. benhamiae*)	0	0	52
TERG_05283T0	Intron1	60S ribosomal protein L32	5116	0	149
TERG_05283T0	Intron2	60S ribosomal protein L32	0	0	321
TERG_05297T0	Intron1	palmitoyltransferase pfa3 (*T. equinum*)	0	0	331
TERG_05299T0	Intron1	glutathione S-transferase, putative (*A. benhamiae*)	39742	8591	1933
TERG_05303T3	Intron1	phospholipase PldA, putative (*T. verrucosum*)	0	0	10
TERG_05345T0	Intron2	60S ribosomal protein L43	0	0	144
TERG_05345T0	Intron3	60S ribosomal protein L43	0	0	288
TERG_05361T0	Intron1	cript family protein (*T. tonsurans*)	20937	184502	2319
TERG_05361T0	Intron2	cript family protein (*T. tonsurans*)	0	0	109
TERG_05393T0	Intron1	hypothetical protein	0	0	142
TERG_05420T0	Intron1	hypothetical protein	0	0	71
TERG_05428T0	Intron1	hypothetical protein	0	0	60
TERG_05430T0	Intron1	hypothetical protein	0	0	28
TERG_05443T0	Intron1	hypothetical protein	13368	0	0
TERG_05455T0	Intron1	C6 finger domain protein, putative (*T. verrucosum*)	15604	12620	264
TERG_05475T0	Intron1	Got1 family protein (*T. tonsurans*)	0	0	118
TERG_05479T0	Intron1	bZIP transcription factor, putative (*T. verrucosum*)	2847	19938	1510
TERG_05483T0	Intron1	transcription elongation factor spt5 (*M. gypseum*)	0	0	97
TERG_05495T0	Intron1	mannose-P-dolichol utilization defect 1 protein (*T. equinum*)	0	0	69
TERG_05509T0	Intron2	aldehyde dehydrogenase ALDH (*A. benhamiae*)	0	0	169
TERG_05509T1	Intron1	aldehyde dehydrogenase ALDH (*A. benhamiae*)	0	0	43
TERG_05511T0	Intron1	AT DNA binding protein (*T. equinum*)	0	0	52
TERG_05512T0	Intron2	hypothetical protein	0	0	36
TERG_05528T0	Intron1	hypothetical protein	2289	0	45
TERG_05539T0	Intron6	Methyltransferase (*T. tonsurans*)	0	6912	481
TERG_05559T0	Intron1	hypothetical protein	0	0	1423
TERG_05562T0	Intron4	2-hydroxychromene-2-carboxylate isomerase, putative (*A. benhamiae*)	0	0	23
TERG_05564T0	Intron1	hypothetical protein	0	0	77
TERG_05570T0	Intron2	WD repeat protein (*T. tonsurans*)	0	0	172
TERG_05576T0	Intron1	cell wall glucanase (Scw11), putative (*A. benhamiae*)	3674	0	19
TERG_05614T0	Intron1	nuclear protein export protein Yrb2, putative (*T. verrucosum*)	2637	0	5
TERG_05634T0	Intron1	mitochondrial protein (*T. tonsurans*)	0	0	51
TERG_05634T1	Intron1	mitochondrial protein (*T. tonsurans*)	0	0	36
TERG_05659T1	Intron2	hypothetical protein	18787	0	13
TERG_05659T1	Intron4	hypothetical protein	0	0	81
TERG_05673T0	Intron1	PaaI-thioesterase (*T. equinum*)	0	0	76
TERG_05673T0	Intron4	PaaI-thioesterase (*T. equinum*)	0	0	26

TERG_05690T4	Intron1	hypoxanthine guanine phosphoribosyltransferase (*T. tonsurans*)	0	0	60
TERG_05690T2	Intron1	hypoxanthine guanine phosphoribosyltransferase (*T. tonsurans*)	0	0	25
TERG_05690T1	Intron1	hypoxanthine guanine phosphoribosyltransferase (*T. tonsurans*)	0	0	18
TERG_05690T0	Intron1	hypoxanthine guanine phosphoribosyltransferase (*T. tonsurans*)	0	0	12
TERG_05695T0	Intron1	hypothetical protein	0	0	1179
TERG_05710T0	Intron3	DUF625 domain-containing protein (*T. equinum*)	0	0	71
TERG_05713T0	Intron1	hypothetical protein	0	7737	1800
TERG_05713T0	Intron2	hypothetical protein	11975	0	0
TERG_05722T0	Intron1	AP-2 adaptor complex subunit mu (*T. tonsurans*)	0	0	1229
TERG_05724T0	Intron1	hypothetical protein	38318	14747	378
TERG_05728T0	Intron1	hypothetical protein	0	0	38
TERG_05729T1	Intron1	ubiquitin-conjugating enzyme E2 6 (*T. equinum*)	0	0	480
TERG_05729T0	Intron2	ubiquitin-conjugating enzyme E2 6 (*T. equinum*)	0	0	354
TERG_05740T0	Intron1	hypothetical protein	0	0	30
TERG_05753T1	Intron1	small GTP-binding protein (*T. tonsurans*)	0	0	13
TERG_05756T0	Intron1	calmodulin-binding protein Sha1, putative (*T. verrucosum*)	0	0	35
TERG_05799T0	Intron5	chlorophyll synthesis pathway protein BchC	0	0	28
TERG_05801T0	Intron1	60S ribosomal protein L15	2156	0	147
TERG_05821T2	Intron4	pyruvate kinase	0	0	47
TERG_05821T0	Intron4	pyruvate kinase	0	0	19
TERG_05825T0	Intron1	40S ribosomal protein S11	829	0	230
TERG_05825T1	Intron1	40S ribosomal protein S11	1526	0	375
TERG_05827T0	Intron1	sphingolipid long chain base-responsive protein PIL1 (*T. equinum*)	0	0	211
TERG_05865T0	Intron1	integral membrane protein, Mpv17/PMP22 family, putative (*T. verrucosum*)	0	0	34
TERG_05865T1	Intron1	integral membrane protein, Mpv17/PMP22 family, putative (*T. verrucosum*)	0	0	31
TERG_05876T0	Intron1	hypothetical protein	0	0	80
TERG_05881T0	Intron1	hypothetical protein	0	0	31
TERG_05882T0	Intron1	C6 transcription factor, putative (*T. verrucosum*)	0	0	37
TERG_05882T0	Intron3	C6 transcription factor, putative (*T. verrucosum*)	0	74	11
TERG_05892T0	Intron1	hypothetical protein	0	0	37
TERG_05894T0	Intron1	peroxisomal AMP binding enzyme (*T. tonsurans*)	0	0	56
TERG_05904T0	Intron1	CobW domain-containing protein (*T. tonsurans*)	0	0	15
TERG_05925T1	Intron1	EF-hand protein (*T. tonsurans*)	0	877	655
TERG_05925T4	Intron1	EF-hand protein (*T. tonsurans*)	0	1200	366
TERG_05925T5	Intron1	EF-hand protein (*T. tonsurans*)	0	846	633
TERG_05926T0	Intron1	C2H2 finger domain protein, putative (*A. benhamiae*)	0	0	75
TERG_05930T0	Intron1	5-formyltetrahydrofolate cyclo-ligase	0	0	78
TERG_05944T2	Intron1	BAR domain-containing protein (*T. tonsurans*)	1227	0	75
TERG_05944T2	Intron2	BAR domain-containing protein (*T. tonsurans*)	13556	2688	56

TERG_05944T2	Intron3	BAR domain-containing protein (*T. tonsurans*)	0	0	72
TERG_05944T1	Intron1	BAR domain-containing protein (*T. tonsurans*)	1648	0	67
TERG_05944T1	Intron2	BAR domain-containing protein (*T. tonsurans*)	10727	1307	56
TERG_05944T0	Intron1	BAR domain-containing protein (*T. tonsurans*)	3293	0	0
TERG_05944T0	Intron2	BAR domain-containing protein (*T. tonsurans*)	10213	3034	3
TERG_05955T0	Intron2	hypothetical protein	0	0	28
TERG_05965T0	Intron3	hypothetical protein	0	0	551
TERG_05970T0	Intron1	hypothetical protein	0	0	634
TERG_05989T0	Intron1	adenylosuccinate lyase	0	0	56
TERG_05996T0	Intron2	AT DNA binding protein (*T. equinum*)	0	0	63
TERG_05998T0	Intron2	chlorophyll synthesis pathway protein BchC	0	0	121
TERG_06004T0	Intron1	hypothetical protein	13538	7885	10
TERG_06006T0	Intron2	hypothetical protein	1470	0	65
TERG_06012T0	Intron1	cytoplasm protein (*T. equinum*)	0	0	28
TERG_06023T0	Intron1	tripeptidyl peptidase SED3 (*T. equinum*)	0	0	69
TERG_06025T0	Intron1	hypothetical protein	0	0	45
TERG_06025T0	Intron2	hypothetical protein	0	0	1626
TERG_06067T0	Intron1	Oxysterol-binding protein (*T. equinum*)	5223	0	50
TERG_06067T0	Intron4	Oxysterol-binding protein (*T. equinum*)	0	0	48
TERG_06072T0	Intron1	SH3 domain-containing protein (*T. tonsurans*)	0	0	52
TERG_06076T0	Intron2	hypothetical protein	133	2265	159
TERG_06087T0	Intron1	mRNA export factor mex67 (*T. equinum*)	0	0	70
TERG_06094T1	Intron1	N-acetyltransferase (*T. tonsurans*)	0	0	24
TERG_06147T0	Intron1	pyridine nucleotide-disulfide oxidoreductase, putative (*A. benhamiae*)	0	0	19
TERG_06148T0	Intron1	protein vts1	0	0	462
TERG_06151T0	Intron1	pyridoxine kinase (*T. equinum*)	26103	10082	115
TERG_06151T0	Intron2	pyridoxine kinase (*T. equinum*)	4530	0	228
TERG_06153T0	Intron1	hypothetical protein	40236	8198	219
TERG_06155T0	Intron1	phosphotransferase (*T. tonsurans*)	609	0	0
TERG_06157T0	Intron4	ATP synthase F1 epsilon subunit (*T. equinum*)	0	0	73
TERG_06182T1	Intron3	topoisomerase I (*T. equinum*)	0	0	15
TERG_06196T0	Intron1	hypothetical protein	0	0	83
TERG_06204T0	Intron4	phosphatase methylesterase (*T. equinum*)	0	0	45
TERG_06211T0	Intron1	DUF498 domain-containing protein (*T. equinum*)	0	0	34
TERG_06236T0	Intron1	40S ribosomal protein S19	0	0	322
TERG_06239T0	Intron1	hypothetical protein	0	0	1978
TERG_06242T0	Intron1	glucanase, putative (*T. verrucosum*)	0	0	24
TERG_06255T0	Intron1	oligosaccharyl transferase subunit Dad1 (*T. tonsurans*)	0	0	104
TERG_06269T0	Intron1	hypothetical protein	0	0	202
TERG_06270T0	Intron3	hypothetical protein	0	0	12
TERG_06273T0	Intron1	hypothetical protein	0	0	40
TERG_06275T0	Intron2	40S ribosomal protein	0	0	165
TERG_06283T0	Intron1	cyclohexanone 1,2-monooxygenase (*T. tonsurans*)	0	0	405

TERG_06293T0	Intron2	vacuolar sorting protein (*T. tonsurans*)	0	0	1287
TERG_06295T0	Intron1	C2H2 finger domain-containing protein (*T. tonsurans*)	98749	50894	7
TERG_06296T1	Intron1	hypothetical protein	0	0	14
TERG_06296T1	Intron2	hypothetical protein	0	0	73
TERG_06307T0	Intron2	ubiquitin conjugating enzyme (*T. tonsurans*)	1347	0	0
TERG_06308T1	Intron1	XPG I-region protein (*M. gypseum*)	0	0	128
TERG_06331T0	Intron1	increased rDNA silencing protein IRS4 (*T. equinum*)	102043	42391	337
TERG_06352T0	Intron1	HAL protein kinase	0	0	91
TERG_06381T0	Intron1	hypothetical protein	13952	12032	151
TERG_06387T0	Intron1	PAS2 (*T. equinum*)	0	0	523
TERG_06390T0	Intron1	sulfite reductase flavoprotein alpha-component (*T. equinum*)	1446	0	0
TERG_06390T2	Intron1	sulfite reductase flavoprotein alpha-component (*T. equinum*)	880	0	33
TERG_06392T0	Intron3	calmodulin	0	0	41
TERG_06406T0	Intron1	hypothetical protein	0	0	67
TERG_06406T0	Intron2	hypothetical protein	0	0	33
TERG_06436T0	Intron1	AAL017W (*M. canis*)	0	0	51
TERG_06472T0	Intron2	ketoreductase (*T. tonsurans*)	0	0	491
TERG_06475T0	Intron1	zinc knuckle domain-containing protein (*T. tonsurans*)	1373	0	0
TERG_06475T0	Intron3	zinc knuckle domain-containing protein (*T. tonsurans*)	1351	0	928
TERG_06475T1	Intron1	zinc knuckle domain-containing protein (*T. tonsurans*)	692	0	21
TERG_06475T1	Intron3	zinc knuckle domain-containing protein (*T. tonsurans*)	982	0	955
TERG_06475T1	Intron5	zinc knuckle domain-containing protein (*T. tonsurans*)	0	0	356
TERG_06475T2	Intron1	zinc knuckle domain-containing protein (*T. tonsurans*)	341	0	0
TERG_06475T2	Intron3	zinc knuckle domain-containing protein (*T. tonsurans*)	0	0	799
TERG_06475T2	Intron5	zinc knuckle domain-containing protein (*T. tonsurans*)	0	0	251
TERG_06493T0	Intron1	mRNA cleavage factor complex component Pcf11 (*T. equinum*)	0	0	8747
TERG_06521T0	Intron1	hypothetical protein	0	0	96
TERG_06528T0	Intron1	Delta(24(24(1)))-sterol reductase (*T. equinum*)	0	0	75
TERG_06540T0	Intron1	glutathione transferase (*T. tonsurans*)	0	0	675
TERG_06568T0	Intron1	phosphoserine transaminase	0	0	224
TERG_06587T0	Intron1	Snf1 kinase complex beta-subunit Gal83 (*T. tonsurans*)	0	0	172
TERG_06601T0	Intron1	flavin-containing amine oxidasedehydrogenase (*T. equinum*)	0	0	105
TERG_06614T1	Intron1	formate dehydrogenase	0	0	47
TERG_06614T1	Intron2	formate dehydrogenase	0	2483	123
TERG_06614T2	Intron1	formate dehydrogenase	0	1150	185
TERG_06614T3	Intron2	formate dehydrogenase	0	1269	136
TERG_06614T0	Intron1	formate dehydrogenase	0	1792	38
TERG_06620T3	Intron2	NADH-ubiquinone oxidoreductase B14 subunit (*T. equinum*)	0	0	153
TERG_06620T1	Intron2	NADH-ubiquinone oxidoreductase B14 subunit (*T. equinum*)	0	0	56

TERG_06622T0	Intron5	hydrolase (*T. tonsurans*)	0	0	1220
TERG_06640T1	Intron1	phosphoribosylamine-glycine ligase	0	0	71
TERG_06644T0	Intron2	glycosyl transferase, putative (*T. verrucosum*)	0	1079	42
TERG_06650T0	Intron2	MFS monocarboxylate transporter, putative (*A. benhamiae*)	0	0	59
TERG_06655T0	Intron2	peptidyl-prolyl cis-trans isomerase cyp15 (*T. equinum*)	0	0	51
TERG_06665T0	Intron3	serine carboxypeptidase (*T. tonsurans*)	0	0	18
TERG_06667T0	Intron1	hypothetical protein	0	644	0
TERG_06681T0	Intron1	glycogen debranching enzyme	0	0	46
TERG_06686T3	Intron1	hypothetical protein	0	226	112
TERG_06686T2	Intron1	hypothetical protein	0	693	181
TERG_06686T1	Intron1	hypothetical protein	0	832	221
TERG_06701T0	Intron1	gamma-glutamyltransferase	0	0	70
TERG_06725T1	Intron1	non-histone chromosomal protein 6	45998	10334	1582
TERG_06725T1	Intron5	non-histone chromosomal protein 6	68870	49473	92048
TERG_06725T2	Intron1	non-histone chromosomal protein 6	42086	6818	1823
TERG_06725T3	Intron1	non-histone chromosomal protein 6	82526	34631	3054
TERG_06752T0	Intron2	serine/threonine protein kinase (*T. tonsurans*)	0	0	2772
TERG_06753T0	Intron2	hypothetical protein	0	0	308
TERG_06768T0	Intron1	pre-mRNA-splicing ATP-dependent RNA helicase PRP28	0	1941	18
TERG_06773T0	Intron3	mRNA splicing protein (*T. tonsurans*)	0	0	157
TERG_06779T0	Intron1	hypothetical protein	0	0	40
TERG_06788T0	Intron2	zinc/iron transporter (*T. equinum*)	0	0	29
TERG_06804T0	Intron1	cgi121 (*T. equinum*)	0	0	242
TERG_06810T0	Intron1	hypothetical protein	0	0	43
TERG_06816T0	Intron2	14-3-3 family protein (*T. tonsurans*)	0	0	330
TERG_06824T0	Intron2	60S ribosomal protein L23	3145	0	139
TERG_06837T0	Intron1	membrane transporter (*T. tonsurans*)	0	0	228
TERG_06844T1	Intron2	40S ribosomal protein S21	0	0	109
TERG_06846T0	Intron2	inosine-5'-monophosphate dehydrogenase	0	9285	3792
TERG_06865T0	Intron1	hypothetical protein	34374	47913	1765
TERG_06875T0	Intron1	hypothetical protein	0	0	51
TERG_06880T0	Intron1	MFS transporter Fmp42, putative (*T. verrucosum*)	0	0	158
TERG_06883T0	Intron4	NAD dependent epimerase/dehydratase family protein (*T. verrucosum*)	0	0	116
TERG_06884T0	Intron3	C6 transcription factor (War1), putative (*T. verrucosum*)	3544	0	41
TERG_06898T0	Intron2	ABC bile acid transporter, putative (*T. verrucosum*)	0	0	19
TERG_06908T0	Intron1	raffinose synthase protein Sip1, putative (*T. verrucosum*)	0	0	58
TERG_06926T0	Intron1	chitosanase (*T. equinum*)	0	0	83
TERG_06965T1	Intron1	hypothetical protein	0	0	100
TERG_06965T0	Intron1	hypothetical protein	361118	181279	1240
TERG_06965T0	Intron2	hypothetical protein	0	0	151
TERG_07015T0	Intron2	MFS transporter, putative (*T. verrucosum*)	0	0	80
TERG_07064T1	Intron1	cytochrome P450 pisatin demethylase, putative (*T. verrucosum*)	0	0	409
TERG_07069T0	Intron1	carbonic anhydrase (*T. tonsurans*)	0	0	65

TERG_07085T1	Intron1	peptidase (*T. tonsurans*)	0	0	42
TERG_07099T0	Intron1	hypothetical protein	95292	12258	8
TERG_07103T1	Intron3	BAP31 domain-containing protein (*T. equinum*)	0	0	43
TERG_07105T0	Intron1	transcription factor TFIIA complex subunit Toa1, putative (*T. verrucosum*)	76	0	177
TERG_07105T0	Intron2	transcription factor TFIIA complex subunit Toa1, putative (*T. verrucosum*)	195533	223651	251
TERG_07135T0	Intron1	hypothetical protein	16331	0	5140
TERG_07136T0	Intron5	Rab geranylgeranyltransferase, beta subunit (*A. benhamiae*)	0	0	254
TERG_07156T1	Intron1	transcription regulator BDF1, putative (*A. benhamiae*)	2780	29489	192
TERG_07156T3	Intron1	transcription regulator BDF1, putative (*A. benhamiae*)	4033	27532	247
TERG_07156T0	Intron1	transcription regulator BDF1, putative (*A. benhamiae*)	0	1652	91
TERG_07159T2	Intron3	geranylgeranyl transferae type II alpha subunit, putative (*T. verrucosum*)	0	0	1090
TERG_07178T0	Intron1	synaptic glycoprotein SC2 (*T. equinum*)	0	0	74
TERG_07178T0	Intron2	synaptic glycoprotein SC2 (*T. equinum*)	0	0	285
TERG_07205T0	Intron2	NADH-ubiquinone oxidoreductase 51 kDa subunit, mitochondrial	0	0	41
TERG_07233T0	Intron2	acriflavine sensitivity control protein acr-2 (*T. equinum*)	0	0	29
TERG_07250T0	Intron1	cell surface receptor/MFS transporter (*T. equinum*)	11099	0	14
TERG_07279T0	Intron1	RNA polymerase II transcription factor B subunit 1 (*T. equinum*)	0	0	32
TERG_07283T0	Intron6	GABA permease (*T. equinum*)	0	0	76
TERG_07288T0	Intron1	hypothetical protein	0	0	173
TERG_07295T0	Intron1	C6 finger domain protein, putative (*T. verrucosum*)	45952	22111	354
TERG_07334T0	Intron2	HIT domain-containing protein (*T. equinum*)	0	0	419
TERG_07339T0	Intron1	WD repeat-containing protein (*T. tonsurans*)	0	0	39
TERG_07347T1	Intron1	La domain family (*T. verrucosum*)	0	0	17
TERG_07347T0	Intron1	La domain family (*T. verrucosum*)	4080	7150	48
TERG_07351T0	Intron1	hypothetical protein	1715	0	4055
TERG_07351T0	Intron2	hypothetical protein	235	0	22679
TERG_07357T0	Intron1	hypothetical protein	0	0	35
TERG_07367T0	Intron1	CMGC/CDK/CDK8 protein kinase	1048	11412	249
TERG_07371T0	Intron2	ADP-ribosylation factor-binding protein GGA2 (*M. gypseum*)	0	0	29
TERG_07371T0	Intron7	ADP-ribosylation factor-binding protein GGA2 (*M. gypseum*)	0	0	53
TERG_07380T0	Intron1	40S ribosomal protein S26E	0	0	26
TERG_07380T0	Intron3	40S ribosomal protein S26E	0	0	13
TERG_07380T1	Intron1	40S ribosomal protein S26E	0	0	10
TERG_07384T0	Intron1	hypothetical protein	3695	0	36
TERG_07395T2	Intron3	WD repeat protein (*T. tonsurans*)	0	0	31
TERG_07402T0	Intron1	hypothetical protein	0	0	57
TERG_07417T0	Intron1	hypothetical protein	0	0	21
TERG_07418T0	Intron1	MFS multidrug transporter (*T. equinum*)	0	0	40
TERG_07447T0	Intron1	hypothetical protein	0	0	48
TERG_07456T0	Intron1	cell wall protein PhiA (*T. equinum*)	0	0	36

TERG_07458T0	Intron1	SAGA-like transcriptional regulatory complex subunit Spt3 (*T. tonsurans*)	0	0	24
TERG_07467T0	Intron1	DUF292 domian containing protein (*M. gypseum*)	6609	0	74
TERG_07482T0	Intron2	ribonuclease H1, putative (*A. benhamiae*)	0	0	260
TERG_07492T0	Intron2	sugar transporter STL1 (*T. tonsurans*)	0	0	34
TERG_07493T0	Intron1	Zinc knuckle transcription factor (CnjB) (*T. tonsurans*)	0	0	643
TERG_07493T0	Intron11	Zinc knuckle transcription factor (CnjB) (*T. tonsurans*)	0	0	643
TERG_07508T0	Intron5	hypothetical protein	0	0	40
TERG_07511T0	Intron1	hypothetical protein	121	0	50
TERG_07542T0	Intron2	hypothetical protein	0	0	410
TERG_07555T0	Intron1	CAMKK protein kinase	1838	0	7
TERG_07575T0	Intron1	CK1/CK1/CK1-G protein kinase	0	0	43
TERG_07575T1	Intron1	CK1/CK1/CK1-G protein kinase	0	0	10
TERG_07575T2	Intron1	CK1/CK1/CK1-G protein kinase	0	0	39
TERG_07576T0	Intron1	histone chaperone RTT106 (*T. tonsurans*)	3218	0	117
TERG_07599T0	Intron1	cell morphogenesis protein Sog2 (*T. tonsurans*)	0	0	31
TERG_07636T0	Intron1	dual specificity phosphatase (*T. tonsurans*)	7612	7238	1147
TERG_07636T0	Intron2	dual specificity phosphatase (*T. tonsurans*)	0	0	87
TERG_07638T0	Intron1	sphingolipid desaturase (*T. tonsurans*)	2061	0	5
TERG_07643T0	Intron1	ubiquinol-cytochrome c reductase complex protein (*T. equinum*)	0	0	240
TERG_07664T1	Intron2	rho GTPase activator Rga (*T. equinum*)	0	0	356
TERG_07678T1	Intron1	endoribonuclease L-PSP (*T. tonsurans*)	0	0	97
TERG_07678T1	Intron2	endoribonuclease L-PSP (*T. tonsurans*)	3287	8572	1186
TERG_07678T0	Intron1	endoribonuclease L-PSP (*T. tonsurans*)	0	0	61
TERG_07678T0	Intron2	endoribonuclease L-PSP (*T. tonsurans*)	2300	2197	183
TERG_07684T0	Intron1	hypothetical protein	1494	0	310
TERG_07692T0	Intron1	hypothetical protein	49238	54671	104
TERG_07719T0	Intron4	hypothetical protein	15712	65655	48
TERG_07797T0	Intron1	amino acid permease (*T. equinum*)	3432	0	0
TERG_07797T0	Intron2	amino acid permease (*T. equinum*)	9028	0	131
TERG_07810T0	Intron1	phospholipase (*T. tonsurans*)	82	0	251
TERG_07824T0	Intron1	BRCT domain-containing protein (*T. equinum*)	0	0	532
TERG_07837T0	Intron1	ribosomal protein S16	0	0	19
TERG_07837T0	Intron2	ribosomal protein S16	0	0	103
TERG_07849T0	Intron2	long-chain-fatty-acid-CoA ligase, putative (*T. verrucosum*)	10975	44896	2068
TERG_07849T4	Intron1	long-chain-fatty-acid-CoA ligase, putative (*T. verrucosum*)	12618	54021	2502
TERG_07862T0	Intron1	dynein light intermediate chain (*T. tonsurans*)	0	0	33
TERG_07875T0	Intron1	integral membrane protein (*T. equinum*)	0	392	97
TERG_07889T0	Intron3	G2/M phase checkpoint control protein Sum2 (*T. tonsurans*)	0	0	109
TERG_07931T0	Intron2	vesicle-mediated transport protein Vid24 (*T. equinum*)	0	0	117
TERG_07932T0	Intron6	vesicle-mediated transport protein Vid24, putative (*T. verrucosum*)	0	0	108
TERG_07943T0	Intron2	hypothetical protein	0	0	35846
TERG_07960T0	Intron1	hypothetical protein	0	0	219

TERG_07997T0	Intron2	acetamidase (*T. tonsurans*)	0	0	38
TERG_08001T0	Intron2	nucleoside hydrolase, putative (*T. verrucosum*)	0	0	173
TERG_08013T0	Intron1	Rox3 mediator complex subunit (*T. equinum*)	0	0	278
TERG_08015T0	Intron5	threonine synthase	0	0	27
TERG_08019T0	Intron1	hypothetical protein	308	6634	1284
TERG_08031T0	Intron1	PHD finger domain-containing protein (*T. equinum*)	0	0	255
TERG_08038T0	Intron1	polysaccharide deacetylase family protein (*T. equinum*)	0	0	82
TERG_08040T0	Intron1	prefoldin subunit 1 (*T. tonsurans*)	5411	0	22
TERG_08064T0	Intron1	choline kinase, putative (*A. benhamiae*)	65871	0	3094
TERG_08101T0	Intron1	TAM domain methyltransferase (*T. equinum*)	2072	0	378
TERG_08101T0	Intron7	TAM domain methyltransferase (*T. equinum*)	2240	0	0
TERG_08108T0	Intron1	40S ribosomal protein S22	0	19	5830
TERG_08115T0	Intron1	hypothetical protein	4225	0	2
TERG_08127T0	Intron1	molybdenum cofactor biosynthesis protein A	0	0	3073
TERG_08128T0	Intron1	hypothetical protein	0	0	224
TERG_08128T0	Intron2	hypothetical protein	0	0	32
TERG_08128T0	Intron3	hypothetical protein	0	0	650
TERG_08139T0	Intron1	NAD dependent epimerase/dehydratase (*M. gypseum*)	1556	0	182
TERG_08140T0	Intron1	methyltransferase type 11 (*M. gypseum*)	4733	0	791
TERG_08153T0	Intron2	hypothetical protein	0	0	11
TERG_08154T0	Intron1	hypothetical protein	0	0	358
TERG_08200T0	Intron3	hypothetical protein	0	0	874
TERG_08238T0	Intron1	copper-activated transcription factor GRISEA, putative (*A. benhamiae*)	1794	0	86
TERG_08250T0	Intron1	cation diffusion facilitator family metal ion transporter, putative (*T. verrucosum*)	0	0	39
TERG_08271T0	Intron1	RNA lariat debranching enzyme, putative (*A. benhamiae*)	0	0	192
TERG_08290T0	Intron2	ATP binding protein (*T. tonsurans*)	0	0	137
TERG_08314T0	Intron1	nucleoporin NUP49/NSP49, putative (*T. verrucosum*)	0	0	46
TERG_08319T0	Intron2	CMGC/SRPK protein kinase	0	0	46
TERG_08323T0	Intron1	serine/threonine-protein phosphatase PP2A catalytic subunit	0	0	166
TERG_08329T0	Intron1	flavin containing amine oxidase (*T. tonsurans*)	0	0	9136
TERG_08341T0	Intron1	hypothetical protein	43090	8892	10291
TERG_08343T0	Intron1	hypothetical protein	0	0	23
TERG_08343T0	Intron2	hypothetical protein	0	0	1650
TERG_08369T0	Intron4	serine/arginine repetitive matrix protein 1 (*M. canis*)	0	0	18
TERG_08381T0	Intron1	NUDIX domain-containing protein (*T. equinum*)	236	0	101
TERG_08383T1	Intron1	hypothetical protein	954336	291210	301
TERG_08423T0	Intron1	hypothetical protein	10780	0	88
TERG_08435T0	Intron2	3-oxoacyl-(acyl-carrier-protein) reductase (*M. canis*)	0	0	307
TERG_08462T3	Intron1	NAD kinase/ATP NAD kinase (*T. tonsurans*)	0	0	57

TERG_08462T2	Intron1	NAD kinase/ATP NAD kinase (*T. tonsurans*)	3024	0	6
TERG_08462T4	Intron1	NAD kinase/ATP NAD kinase (*T. tonsurans*)	0	0	79
TERG_08484T0	Intron6	vacuolar protein sorting vps16 (*T. tonsurans*)	0	0	1553
TERG_08516T0	Intron1	hypothetical protein	0	0	299
TERG_08542T0	Intron1	GTP-binding protein ypt2	0	0	130
TERG_08551T0	Intron2	NADH-ubiquinone oxidoreductase 21 kDa subunit (*T. tonsurans*)	0	0	50
TERG_08581T0	Intron1	F-box protein (*T. tonsurans*)	1753	0	48
TERG_08655T0	Intron1	ADP,ATP carrier protein	0	0	481
TERG_08655T0	Intron2	ADP,ATP carrier protein	2479	0	143
TERG_08656T0	Intron1	hypothetical protein	0	0	139
TERG_08672T0	Intron8	HFR-3 (*M. canis*)	0	0	45
TERG_08678T0	Intron1	RNP domain-containing protein (*T. tonsurans*)	0	0	23
TERG_08678T0	Intron2	RNP domain-containing protein (*T. tonsurans*)	2879	0	87
TERG_08678T1	Intron1	RNP domain-containing protein (*T. tonsurans*)	0	0	82
TERG_08678T1	Intron2	RNP domain-containing protein (*T. tonsurans*)	5583	0	11
TERG_08678T2	Intron1	RNP domain-containing protein (*T. tonsurans*)	0	0	23
TERG_08678T2	Intron2	RNP domain-containing protein (*T. tonsurans*)	2732	0	118
TERG_08697T0	Intron1	serine/threonine protein kinase	0	0	720
TERG_08738T0	Intron2	TBP interacting domain protein, putative (*A. benhamiae*)	0	0	79
TERG_08747T0	Intron1	hypothetical protein	0	0	17
TERG_08756T0	Intron4	cercosporin toxin biosynthesis protein (*T. equinum*)	0	1991	427
TERG_08761T0	Intron2	protein bfr2	0	0	103
TERG_08761T1	Intron1	protein bfr2	0	0	133
TERG_08825T0	Intron1	bZIP transcription factor JlbA/IDI-4 (*T. verrucosum*)	5393	0	0
TERG_08825T0	Intron2	bZIP transcription factor JlbA/IDI-4 (*T. verrucosum*)	26873	11533	318
TERG_08848T0	Intron1	thioredoxin, putative (*A. benhamiae*)	0	0	198
TERG_08855T0	Intron1	hypothetical protein	16819	51164	705
TERG_08867T0	Intron2	chitin synthase (*T. tonsurans*)	0	0	56
TERG_08884T0	Intron1	GPI-anchored cell surface glycoprotein, putative (*T. verrucosum*)	0	7009	6
TERG_08898T0	Intron1	hypothetical protein	0	0	73
TERG_08906T0	Intron1	hypothetical protein	57088	152552	470
TERG_08959T0	Intron1	hypothetical protein	1279	8322	878
TERG_08959T0	Intron2	hypothetical protein	0	308	271
TERG_08975T0	Intron1	importin beta-1 subunit (*T. tonsurans*)	0	0	73
TERG_08982T0	Intron1	hypothetical protein	0	0	123
TERG_08986T0	Intron1	hypothetical protein	0	0	200
TERG_08991T0	Intron1	ATPase 2 nuclear control (*M. gypseum*)	0	14128	57
TERG_11512T0	Intron5	General negative regulator of transcription subunit 4 (*T. tonsurans*)	3920	0	9552
TERG_11518T0	Intron1	glucokinase, putative (*T. verrucosum*)	0	0	2877
TERG_11520T0	Intron2	3' exoribonuclease (*T. tonsurans*)	0	0	282
TERG_11522T0	Intron1	hypothetical protein	0	0	47

TERG_11546T0	Intron1	hypothetical protein	8161	0	1106
TERG_11592T0	Intron1	palmitoyl-protein thioesterase (*T. tonsurans*)	2260	0	596
TERG_11600T0	Intron1	MFS transporter (*T. tonsurans*)	0	0	60
TERG_11628T0	Intron1	glutamine synthetase	11890474	16196158	51090
TERG_11628T0	Intron3	glutamine synthetase	16091	6281	2054
TERG_11642T0	Intron1	forkhead transcription factor (*T. tonsurans*)	0	0	68
TERG_11648T0	Intron1	phosphatidylserine decarboxylase (*T. tonsurans*)	0	0	24
TERG_11699T0	Intron1	hypothetical protein	0	0	311
TERG_11727T0	Intron3	phosphotransferase (*M. canis*)	0	14	12649
TERG_11733T0	Intron1	methionine-tRNA synthetase (*T. equinum*)	618	0	2
TERG_11756T0	Intron2	DNA repair protein Rad7, protein (*A. benhamiae*)	0	0	372
TERG_11841T0	Intron5	hypothetical protein	0	0	119
TERG_11879T0	Intron1	hypothetical protein	5513	0	53285
TERG_11937T0	Intron1	sister chromatid separation protein (*T. tonsurans*)	0	0	206
TERG_11980T0	Intron1	hypothetical protein	3714	0	19
TERG_11982T0	Intron5	ankyrin repeat protein (*T. verrucosum*)	197087	89281	3017
TERG_11991T0	Intron1	hypothetical protein	14673	33510	1819
TERG_12004T0	Intron2	lipase (*T. tonsurans*)	22010	119042	37518
TERG_12026T0	Intron2	nonsense-mediated mRNA decay protein 2 (*T. tonsurans*)	0	0	13
TERG_12028T0	Intron2	metallothionein family protein (*T. equinum*)	0	0	283
TERG_12030T0	Intron2	DUF821 domain protein (*T. verrucosum*)	0	0	8251
TERG_12036T0	Intron1	CMGC/SRPK protein kinase (*T. equinum*)	0	0	31
TERG_12054T0	Intron2	HEAT repeat protein (*A. benhamiae*)	0	0	28
TERG_12078T0	Intron1	hypothetical protein	112896	34542	236
TERG_12112T0	Intron1	hypothetical protein	0	0	146
TERG_12164T0	Intron1	hypothetical protein	0	0	46
TERG_12164T0	Intron2	hypothetical protein	126105	31484	13802
TERG_12186T0	Intron1	hypothetical protein	447	0	0
TERG_12201T0	Intron1	hypothetical protein	25968	17062	238
TERG_12222T0	Intron1	polyketide synthase (*T. equinum*)	0	0	281
TERG_12269T0	Intron1	curved DNA-binding protein (*T. tonsurans*)	0	0	467
TERG_12291T0	Intron1	mitochondrial cytochrome b2 (*T. tonsurans*)	4635	15557	193
TERG_12291T1	Intron1	mitochondrial cytochrome b2 (*T. tonsurans*)	9211	15246	194
TERG_12291T1	Intron2	mitochondrial cytochrome b2 (*T. tonsurans*)	0	0	13
TERG_12303T0	Intron2	myosin-1	111371	131355	15365
TERG_12349T0	Intron1	hypothetical protein	0	641	1337
TERG_12351T0	Intron1	hypothetical protein	23831	18767	4987
TERG_12427T0	Intron2	T-complex protein 1 (*T. tonsurans*)	0	0	506
TERG_12442T0	Intron1	hypothetical protein	0	0	16
TERG_12458T0	Intron1	C6 transcription factor, putative (*T. verrucosum*)	0	0	137
TERG_12477T0	Intron1	ABC bile acid transporter, putative (*T. verrucosum*)	0	0	84
TERG_12551T0	Intron1	DUF250 domain membrane protein (*T. equinum*)	17318	0	455
TERG_12568T0	Intron3	hypothetical protein	0	0	1221
TERG_12591T0	Intron1	peptidase S8 family protein (*T. equinum*)	20164	20794	1027
TERG_12616T0	Intron2	ankyrin repeat-containing protein (*T. equinum*)	6131	10869	30

TERG_12633T0	Intron1	conserved lysine-rich protein, putative (*A. benhamiae*)	0	0	114
TERG_12641T0	Intron2	Poly(A) polymerase PAPalpha (*T. tonsurans*)	0	0	72
TERG_12642T0	Intron2	Poly(A) polymerase PAPalpha (*T. tonsurans*)	0	0	14
TERG_12645T0	Intron1	hypothetical protein	8847	15472	1207
TERG_12681T0	Intron1	alpha-mannosidase (*T. tonsurans*)	0	0	49
TERG_12694T0	Intron1	hypothetical protein	0	0	22

Filter = at least one condition with more than 10 read counts.

Supplementary Table S5. List of primers used in RT-PCR and/or qRT-PCR assays.

ID[(*)]	Gene Product Name		Primers (5' - 3')	Amplicon (bp)	Efficiency (%)	Concentration (nM)
TERG_01184	fatty acid-binding protein (*T. equinum*)	FW:<br>REV:	AGATCGACGCTTTCCCTTC<br>CGTTGGTGAGGGTGAAGG	126	99.42	300
TERG_01252	catalase A	FW:<br>REV:	GGAGAGCTATGGTGTGGTTAC<br>GTTCCCTCTCGTAATTCCTGTG	143	100.45	500
TERG_01281	malate synthase, glyoxysomal	FW:<br>REV:	ACCCCTAACCAACTCTTTGTC<br>GAAGCCATCCCTCCATGTAAG	145	102.92	700
TERG_01463	cytochrome c peroxidase (*T. tonsurans*)	FW:<br>REV:	CCACATCCGACACATCTTCTAC<br>GAGAATCGGGTTGGGTTGTT	141	113	200
TERG_01475	DNA topoisomerase 2	FW:<br>REV:	TTTGGAACCCAGCGGTAACT<br>CGAGCACTGGCACTGTCAGA	63	93.69	200
TERG_02214	carboxypeptidase 2	FW:<br>REV:	ACGAAGGCGTCGTCACTACT<br>AAAGCAACAAAGGCAAGAGC	118	101.89	700
TERG_02601	Phosphoglucomutase (*T. tonsurans*)	FW:<br>REV:	CGGCAGATAATTCCGGTGTAA<br>CTAGGAGATGCGATGCCAATAG	141	114	200
TERG_02601	Phosphoglucomutase exon-4 usage	FW:<br>REV:	AGGTATACATGTGTGGTGG<br>CATTATCTTGTGCCGGGTT	125	95.17	70/100
TERG_03078	cytochrome P450 oxidoreductase OrdA-like, putative (*T. verrucosum*)	FW:<br>REV:	CCATGACAATACTCTACGGCTC<br>GGCTGATCCTTGGGTACTTTG	142	95.82	800
TERG_03719	MFS sugar transporter (*T. tonsurans*)	FW:<br>REV:	TTGAGAAGGTTGAAGGGCTG<br>ACTGTTTCGTCCATTCCCTG	145	96.84	700
TERG_04038	acyl-CoA dehydrogenase (*T. tonsurans*)	FW:<br>REV:	ACTCTAAGGATTGCTGATGGC<br>CATTCAGTAACCTATCCGCCTC	133	100.05	700
TERG_04960	glutathione transferase (*A. benhamiae*)	FW:<br>REV:	CCCAAACACCGACCTGAA<br>TCGTTGGTATCGTGTGGAAAG	105	107.35	200
TERG_05575	MFS multidrug transporter (*T. tonsurans*)	FW:<br>REV:	GAGGTTTGTCTGGATCGTCTG<br>CCCTGATTCAAGTCATACGAG	87	99.23	800
TERG_05742	DNA-dependent RNA polymerase II RPB140	FW:<br>REV:	CCTCTACACTTTCCCATCTTCG<br>TGACAAGACCACAAGCCTG	59	94.99	200
TERG_06509	glutamate synthase (*T. tonsurans*)	FW:<br>REV:	CCCACTCTACCCTTGCTATTG<br>ATCTTCACCGCCTTCGCCAGTATT	71	98.74	800
TERG_06625	serine protease, putative (*A. benhamiae*)	FW:<br>REV:	GCTCGATGTTAGGCATTACC<br>GGTGGCGAACTTATAGGACTG	119	95.60	400
TERG_06846	inosine-5'-monophosphate dehydrogenase	FW:<br>REV:	GTGGGCGGACAGAGTTT<br>CGGCAATGAGATATGGGACGAA	481 / 684[(as)]	-	-
TERG_07544	lipase (*T. tonsurans*)	FW:<br>REV:	TGCCAAAGACGGTAAAGAGG<br>TGAGAACAGAGTCGAAAACCG	113	95.40	800
TERG_07691	sterol carrier protein (*T. tonsurans*)	FW:<br>REV:	GTTTGGAAATGCTGGTGTCG<br>TGTACTCGGTTTGGAATTGGG	128	97.92	700
TERG_08130	ABC ATPase (*T. equinum*)	FW:<br>REV:	AAAGGCCGGAGTCCATTATG<br>TCGAGGTCAACTTCAACATCAG	150	98.45	800
TERG_08557	carboxypeptidase S1, putative (*A. benhamiae*)	FW:<br>REV:	GGTTTCGCTTGTTTACGGTG<br>AATTCGTGGGTGTCAGTGTAG	96	100.03	700
TERG_11637	isocitrate lyase	FW:<br>REV:	TATCCATCGTCTGGGAGAGC<br>GCTTAGCGTATGCCTTGGAA	144	108.63	700
TERG_12530	3-ketoacyl-CoA thiolase peroxisomal A (*T. tonsurans*)	FW:<br>REV:	CTCCTTATGAAGCGTTCTACCG<br>AGAGGCTTAACACCGACAAC	89	98.09	700

[(*)] TERG: gene accession number at the *T. rubrum* genome database at the Broad Institute.
[(as)] Alternative splicing intron retention (intron-2) by RT-PCR.
